# Development of a nursing follow-up checklist for adult ECMO-treated discharged patients: a Delphi consensus and feasibility study

**DOI:** 10.3389/fmed.2026.1779603

**Published:** 2026-03-25

**Authors:** Yanting Zhang, Jing Ma, Sen Li, Zhaoyang Li, Yiyi Zhou, Chen Chen, Jin Li, Xinbo Ding, Chao Tian

**Affiliations:** 1 Department of Critical Care Medicine, Zhongnan Hospital of Wuhan University, Wuhan, China; 2 Clinical Research Center of Hubei Critical Care Medicine, Wuhan, China

**Keywords:** extracorporeal membrane oxygenation, ECMO, discharge, follow-up checklist, Delphi consensus

## Abstract

**Aim:**

Develop a nursing follow-up checklist for adult patients discharged after Extracorporeal membrane oxygenation (ECMO) surgery, providing a scientific and effective assessment tool for the continuous care services of surviving adult patients post-ECMO discharge.

**Methods:**

The first draft of the nursing follow-up checklist for adult patients discharged after ECMO therapy was developed through literature review, semi-structured interviews, and group discussions. In October 2025, two rounds of Delphi expert consultations were conducted, and modifications and improvements were made based on expert opinions and clinical realities, resulting in the final version of the follow-up checklist after content revision. Using convenience sampling, six nurses and ten adult patients discharged after ECMO therapy from a tertiary Grade A hospital in Wuhan City were selected as participants in November 2025 to preliminarily test the feasibility of the follow-up checklist, further evaluating and refining its content.

**Results:**

The expert response rates for the two rounds of correspondence were 86.67 and 100.00%, respectively. The expert authority coefficients were 0.93 and 0.90, with Kendall’s concordance coefficient (W)s of 0.187 (χ^2^ = 209.598) and 0.232 (χ^2^ = 313.055) (*p* < 0.001). The developed nursing follow-up checklist for adult patients discharged after ECMO therapy includes four primary categories—physiological status, psychological status, living conditions, and social and family support—along with 20 secondary items and 83 tertiary items. Follow-up participants reported that the content of the checklist was comprehensive, and nurses expressed high satisfaction with its use.

**Conclusion:**

The checklist is comprehensive in content, demonstrating good content validity, clinical feasibility, and high acceptability.

## 1 Introduction

Extracorporeal membrane oxygenation (ECMO) is an extracorporeal life support technique used to support patients with severe cardiac or respiratory failure, thereby aiding the recovery of critically ill patients and improving survival rates (1–3). Although ECMO is an expensive and invasive intervention that has improved short-term survival, healthcare providers often remain unaware of post-discharge outcomes (4, 5). International studies indicate (6) that discharge survival rates for patients treated with veno-venous ECMO (V-VECMO), veno-arterial ECMO (V-AECMO), and extracorporeal cardiopulmonary resuscitation (ECPR) are 58, 41, and 29%, respectively. In 2015, Tramm et al. (7) systematically reviewed literature on whether ECMO treatment improves patient survival, noting that none of the four included randomized controlled trials investigated long-term quality of life. Von Bahr et al. (8) conducted a retrospective study of 168 critically ill patients treated with ECMO and found that the highest mortality period was within the first few months after illness onset, after which mortality stabilized, suggesting that 90 days could serve as a critical threshold for long-term survival. Currently, Chinese scholars predominantly focus on factors influencing patient survival outcomes during ECMO treatment (9, 10), with limited research on the status and determinants of long-term quality of life in these patients. However, long-term quality of life is closely linked to nursing interventions (11). Spangenberg et al. (12) proposed that long-term management of ECMO-treated patients should resemble chronic disease care, while Chen et al. (13) emphasized in their qualitative study that multidisciplinary evidence-based interventions should be implemented post-discharge to address physical, psychological, and social challenges faced by ECMO survivors. Additionally, research shows that standardized follow-up checklists—with well-structured designs—can regulate follow-up processes and provide effective guidance for patients (14).

Therefore, this study aims to develop and clinically apply a nursing follow-up checklist for adult patients discharged after ECMO therapy through literature review, semi-structured interviews, expert consultations, and revisions. The objective is to provide scientifically standardized follow-up support in clinical practice, establish a foundational basis for formulating targeted interventions and recommendations, thereby improving post-discharge quality of life and enhancing long-term survival rates.

## 2 Methods

### 2.1 Develop a preliminary draft of the nursing follow-up checklist for adult patients discharged after ECMO therapy

#### 2.1.1 Establish a research team

The research team consists of 8 members, including: 1 Head Nurse (overall project coordination and quality control); 2 Ward Nurse Managers (coordinating the expert panel list and revising checklist content); 2 Clinical Educators (developing literature review protocols and finalizing interview guides); 2 Key Team Members (designing consultation questionnaires and compiling results); 1 Nurse with a Master’s degree (literature development and interview guide finalization).

#### 2.1.2 Structured integrative evidence review for checklist development

Here is the translation of the text into academic English, suitable for a research paper’s “Methods” section.

##### 2.1.2.1 Study design and scope

Based on the PCC (Population, Concept, Context) framework, this study defined the scope of the literature search and evaluation questions, serving as the core basis for data extraction. The study population comprised adult patients (≥18 years) with severe cardiopulmonary failure who received ECMO therapy and were discharged from the hospital. The key focus areas included post-discharge follow-up, rehabilitation nursing, complication monitoring, psychological and social support, and treatment adherence among ECMO survivors. The intended application context was clinical nursing practice for ECMO patients, covering both in-hospital and out-of-hospital settings within domestic and international medical institutions. The extracted outcomes and dimensions encompassed five core domains of the follow-up checklist: monitoring of physiological complications, psychological status assessment, activities of daily living, social and family support, and long-term rehabilitation outcomes.

##### 2.1.2.2 Literature inclusion criteria

The literature inclusion criteria were established to strictly align with the research objectives. Specifically, eligible studies included those with a study population of adult ECMO patients (≥18 years) who possessed post-discharge follow-up data. Regarding study design, the scope encompassed clinical practice guidelines, expert consensus statements, systematic reviews, randomized controlled trials, cohort studies, cross-sectional studies, case series with a sample size of at least 10, and qualitative studies. The ECMO modalities covered included VA-ECMO, VV-ECMO, and ECPR. Furthermore, the content of the studies had to address physiological, psychological, daily living, or social support aspects relevant to ECMO patients after discharge. The language was restricted to Chinese and English, and the studies were required to clearly report a post-discharge follow-up duration of at least 1 week.

##### 2.1.2.3 Literature exclusion criteria

Conversely, literature exclusion criteria were applied through multidimensional screening. Studies were excluded if their study population focused on pediatric ECMO patients or critically ill non-ECMO patients. Regarding study design, excluded types included case reports with a sample size of less than 10, conference abstracts, and review articles or commentaries lacking original data. Studies that focused solely on in-hospital ECMO treatment without any content related to post-discharge follow-up were also excluded. Additionally, literature published in languages other than Chinese or English was excluded, as were low-quality studies characterized by incomplete data or unclear research designs.

Search terms: “extracorporeal membrane oxygenation/ECMO,” “rehabilitation/follow-up/follow-up/family/household/home/out-of-hospital/after discharge/continuing care/continuing care/transitional care/quality of life.” The search timeframe covered database inception to June 2025. Two reviewers, trained in JBI evidence-based nursing, independently conducted title/abstract screening and full-text screening. All discrepancies during the screening process were resolved by a third senior reviewer with over 10 years of experience in critical care nursing research. NoteExpress was used for literature management, including duplicate removal, maintaining screening records, and categorizing included and excluded studies.

##### 2.1.2.4 Literature quality appraisal

To ensure methodological consistency and transparency, this study strictly matched the corresponding quality assessment tools to the research types and explicitly clarified the impact of study quality on data extraction. For quantitative studies, cohort studies were evaluated using the CASP checklist from the JBI suite, cross-sectional studies were assessed using AHRQ criteria, and quasi-experimental studies and randomized controlled trials (RCTs) were evaluated using the respective JBI tools designed for these designs. Qualitative studies were assessed using the JBI critical appraisal tool for qualitative research, supplemented by the COREQ checklist to ensure reporting completeness; however, COREQ was not used for assessing the risk of methodological bias. Clinical guidelines and expert consensus were quality-assessed using the AGREE II instrument.

Furthermore, study quality was categorized into “high” and “moderate” to guide data extraction. The criteria for high-quality studies were defined as follows: guidelines achieving a standardized AGREE II score of ≥60% across all domains, with no domain scoring ≤30%; systematic reviews meeting ≥10 out of 11 appraisal criteria (with the assessment of publication bias being optional); randomized controlled trials (RCTs) and cohort studies achieving a compliance rate of ≥80%; and qualitative studies meeting all 10 JBI appraisal criteria. The criteria for moderate-quality studies were defined as: guidelines with 4–5 domains scoring ≥60% on the AGREE II; systematic reviews meeting 7–10 criteria; RCTs and cohort studies with a compliance rate of 60–79%; and qualitative studies meeting 7–9 JBI criteria. Low-quality studies failing to meet the above standards were excluded during the initial screening phase. Data extraction prioritized evidence from high-quality studies, while moderate-quality studies served only as supplementary data; their extracted items were retained solely after expert verification.

To mitigate bias arising from heterogeneous evidence, this study adopted the following strategy to integrate study quality with the construction of checklist items. Low-quality studies failing to meet the criteria for high or moderate quality were excluded during the literature screening phase and excluded from item extraction, thereby ensuring that all items were grounded in reliable evidence. Duplicate items with identical content were consolidated; complementary items of the same category were logically integrated. In cases of conflicting recommendations, priority was given to evidence from high-quality studies, and the final items were determined in conjunction with expert opinion. Individual standalone items were retained in their original wording.

#### 2.1.3 Semi-structured interviews

From August to September 2025, purposive sampling was employed to select conscious patients discharged from the ICU following Extracorporeal Membrane Oxygenation (ECMO) treatment at a tertiary Grade-A hospital in Wuhan for semi-structured interviews. The sample size was determined based on the principle of data saturation, resulting in the inclusion of 7 participants (4 patients and 3 family proxies; 3 males and 4 females; aged 32–68 years; ECMO indications included coronary heart disease, severe pneumonia, fulminant myocarditis, and myocardial infarction). All participants voluntarily signed informed consent forms, and permission to record the interviews was obtained prior to the sessions. Each interview lasted 20 to 30 min. A preliminary interview guide was developed through a literature review and refined through discussion within the research team. The content covered current recovery status and desired post-discharge medical guidance, difficulties and discomfort encountered during recovery, factors influencing the acceptance of follow-up recommendations, and psychological feelings after discharge.

Audio recordings were transcribed verbatim within 24 h post-interview. Thematic analysis was performed to extract themes, supplementing the follow-up checklist content.

#### 2.1.4 Delphi expert consultation questionnaire

##### 2.1.4.1 Development of the expert consultation questionnaire

The expert consultation questionnaire consisted of four parts: an introduction, a survey on expert demographics, an expert rating scale, and an assessment of expert authority. The introduction outlined the research objectives, significance, and instructions for completing the questionnaire. The demographic survey collected core information regarding the experts’ age, gender, educational background, professional title, years of clinical experience, research focus, and status as graduate supervisors. The expert rating scale employed a 5-point Likert scale to evaluate the importance of each item in the follow-up checklist (with 1 indicating “extremely unimportant” and 5 indicating “extremely important”), and included space for experts to provide suggestions for revision and new ideas. The authority assessment form required experts to indicate their familiarity with the research content and the basis of their judgments.

##### 2.1.4.2 Selection of experts for the Delphi consultation

Inclusion criteria for experts: Individuals with over 5 years of experience in critical care nursing, clinical/ECMO nursing, or management; holding an intermediate-level professional title or higher; possessing a bachelor’s degree or above; and willing to participate voluntarily and complete both rounds of consultation. Use purposive sampling.

##### 2.1.4.3 Conducting expert consultation

Two rounds of expert consultation were conducted in October 2025. For the first round, questionnaire items were formatted into a Delphi consultation form and distributed to experts via email, WeChat, or face-to-face delivery of paper copies. Experts were requested to provide feedback within 1 week. Their opinions were then integrated, analyzed, and discussed by the research team. Two weeks later, the second-round questionnaires were distributed using the same method. The process was repeated, with responses collected within 1 week and further group discussions held to summarize expert suggestions. Upon receipt of each questionnaire, immediate verification was performed. If any omissions or unclear entries were identified, the corresponding expert was promptly contacted for clarification.

### 2.2 Preliminary application of the nursing follow-up checklist for adult patients discharged after ECMO therapy

Using convenience sampling, six nurses from a tertiary Grade A hospital in Wuhan City were selected in November 2025 to conduct telephone follow-ups with ten adult patients discharged after ECMO therapy, with each nurse following up approximately one to two patients. This process served as a further evaluation of the follow-up checklist. Prior to the follow-ups, the research team provided standardized training to ensure that participating nurses correctly understood the content and usage of the checklist items. During the follow-up process, nurses recorded any issues encountered and the duration of each call in the remarks section, aiming to assess the practicality of the checklist contents for future optimization. Upon completion, nurses completed an evaluation form assessing the comprehensiveness, relevance, organization, simplicity, clarity, usefulness, and helpfulness of the follow-up checklist. This evaluation tool, adapted from relevant checklist assessment questionnaires (15), comprised seven items rated on a Likert 5-point scale ranging from “Strongly Disagree” (1) to “Strongly Agree” (5), with a maximum score of 35 indicating higher satisfaction. Additionally, research team members engaged in face-to-face discussions with the nurses to gather feedback on challenges or suggestions during implementation, facilitating further revisions.

Follow-up Participant Criteria: Inclusion criteria: Adult patients (≥18 years of age) who were successfully discharged after ECMO and are conscious and able to cooperate with follow-up. Exclusion criteria: Patients with severe cognitive impairment or psychiatric illness that prevents cooperation, those lost to follow-up after discharge, or those with an expected survival time of <30 days.

### 2.3 Ethical approval

This study was conducted in accordance with the Declaration of Helsinki and met ethical standards for medical research. It received approval from the Ethics Committee of Zhongnan Hospital of Wuhan University, with the ethical number [2025001K]. All participants provided their informed consent prior to involvement in the study.

### 2.4 Statistical methods

Data analysis was performed using SPSS version 26.0 software. Continuous variables following a normal distribution are described as mean ± standard deviation, while non-normally distributed continuous variables are presented as median and interquartile range. Categorical data are summarized using frequencies and proportions. Key evaluation indicators for the expert consultation included: expert response rate (response rate), expert authority level, degree of agreement among experts (Kendall’s W), and concentration of expert opinions. The effective questionnaire return rate reflected expert engagement, with a threshold of ≥70% indicating high participation. Expert authority was determined by both judgment criteria (Ca) and familiarity with the subject matter (Cs), calculated as (Ca + Cs)/2. Kendall’s W assessed consensus, where smaller *p*-values indicated greater reliability. Concentration of expert opinion was evaluated through mean score (Mj), coefficient of variation (CV = SD/mean), and full-score ratio (Kj, defined as the percentage of items rated at maximum importance). An item was retained if it met all three criteria: Mj > 3.50, CV < 0.25, and Kj > 20%. If only one or two criteria were satisfied, further expert consultation or group discussion determined retention; otherwise, the item was excluded (16, 17). Given that this study aimed to develop a “clinical documentation tool” rather than a psychometric scale, traditional validity assessments requiring theoretical constructs, measurement dimensions, criterion references, or scoring systems were not applicable. Instead, content validity (via expert review) and inter-rater reliability were prioritized to ensure comprehensiveness of recorded content and consistency in completion outcomes—sufficient for meeting clinical/research quality standards. Therefore, no additional validity tests were conducted. This checklist is a clinical documentation tool designed to standardize the collection of multi-dimensional data on post-ECMO discharge patients, rather than a psychometric scale measuring abstract concepts (e.g., quality of life, anxiety). Therefore, COSMIN-style construct validity and criterion validity are not the focus of this study at present, as these validity assessments rely on the measurement of abstract latent variables. However, clinical documentation tools still have measurable properties, and this study has systematically assessed three core measurement properties: content validity, feasibility, and acceptability.

## 3 Results

### 3.1 Literature review and semi-structured interview results

**Figure 1:**
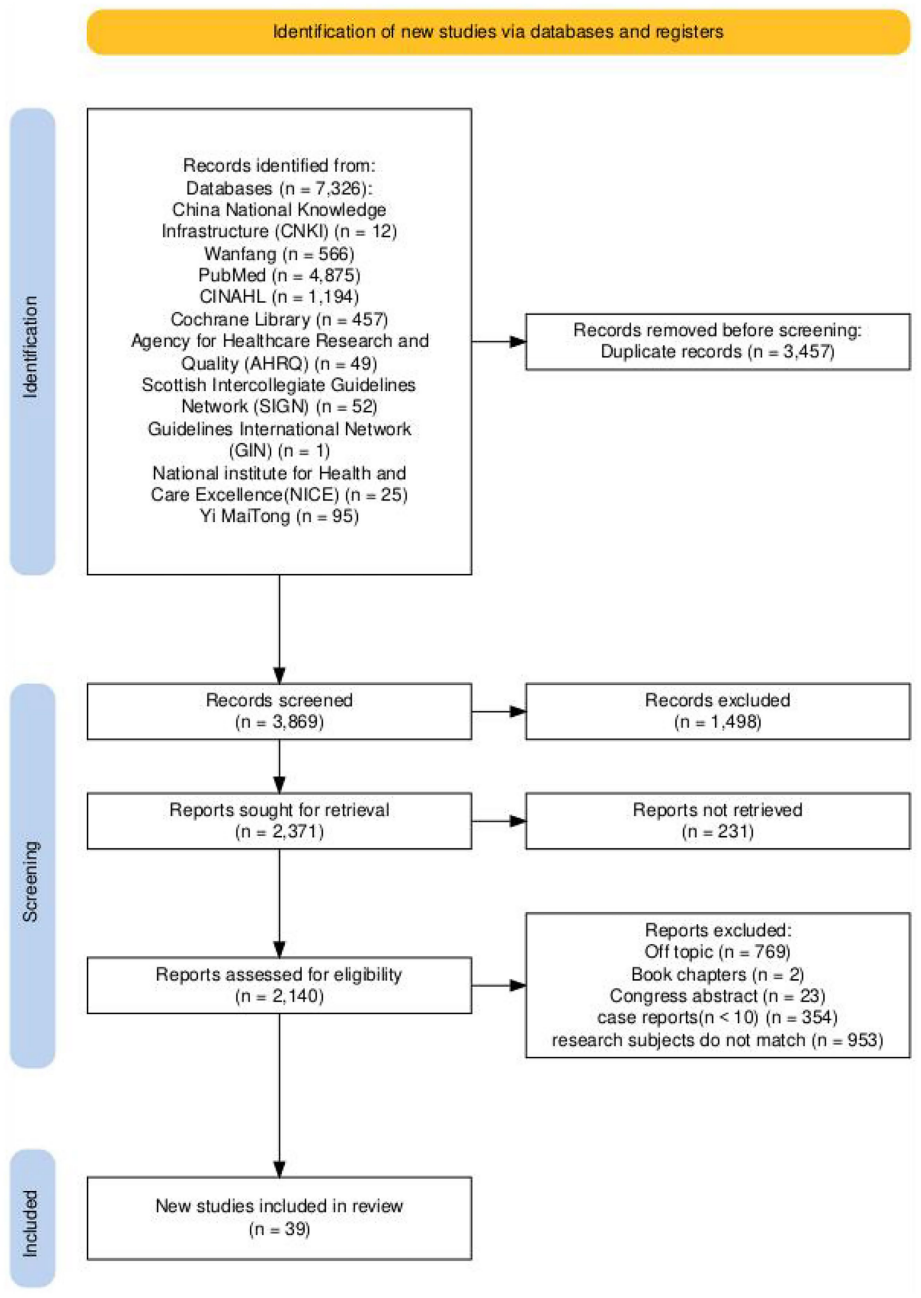
Literature screening flowchart (a total of 7,326 articles were initially retrieved, and 2,188 duplicates were removed using EndNote 20, resulting in 5138 articles for title/abstract screening. After excluding 4,892 articles that did not meet the inclusion criteria, 246 articles were included for full-text screening. Finally, 207 articles were excluded for reasons such as inconsistent study population, no post-discharge follow-up content, and small sample size, with 39 high/moderate quality studies included in the final evidence synthesis).

A total of 7,326 articles were initially identified through the search strategy (detailed in Supplementary Material 1). After duplicate checking, application of inclusion/exclusion criteria, and quality assessment (as outlined in Supplementary Material 2), 39 studies were ultimately included: 1 guideline, 1 quasi-experimental study, 3 qualitative studies, 5 systematic reviews, 1 randomized controlled trial, 23 cohort studies, and 5 cross-sectional studies. The literature screening process is illustrated in Figure 1. Content extraction and synthesis (Supplementary Material 3) yielded a preliminary draft of the nursing follow-up checklist for adult patients discharged after ECMO therapy, comprising 4 primary domains, 16 secondary items, and 62 tertiary items. Through semi-structured interviews, an additional 2 secondary items and 3 tertiary items were incorporated, resulting in a final preliminary version consisting of 4 primary domains, 18 secondary items, and 65 tertiary items (see Supplementary Tables S1, S2).

### 3.2 Delphi expert consultation results

#### 3.2.1 Basic information of the consulted experts

A total of 13 experts were included in this study, representing five regions: Hubei, Henan, Guizhou, Shandong, and Hunan. Among them, there were 7 males and 6 females (note: original text states “7 males and 6 females”, which sums to 14; assuming a typographical error, corrected here for consistency). Educational background: 8 held master’s degrees or higher, while 5 had bachelor’s degrees. The age range was from 30 ~ 41 years old, with an average of 34.38 ± 3.07 years. Their ICU with ECMO work experience varied between 5 ~ 18 years, averaging at 10.77 ± 3.94 years. Professional titles: 10 were intermediate-level, and 3 were senior-level.

#### 3.2.2 Expert response rate

For the first round of Delphi consultation, a total of 18 experts were initially invited through multiple channels. Three declined participation due to scheduling conflicts or lack of ECMO-related experience, leaving 15 who agreed to participate. A total of 13 valid questionnaires were returned (effective response rate: 86.67%). All 13 participants from the first round were invited for the second round, achieving a 100% response rate. Eleven experts (83.62%) provided 43 comments. In the second round, all 13 questionnaires sent out were returned, achieving an effective recovery rate of 100%. Ten experts (76.92%) contributed 33 suggestions. The expert response rate exceeded 70% for both rounds, indicating high engagement and strong commitment to the research among the participants.

To avoid authority bias and groupthink, and to ensure the independence of expert judgments, this study employed a fully anonymous rating process throughout: ① First-round consultation: Questionnaires were distributed via encrypted email or password-protected online forms. Expert names and institutional affiliations were removed from the rating sheets. Only the research team had access to participants’ identities, which were not disclosed to other experts. ② Second-round consultation: Aggregated feedback (e.g., item mean scores, coefficient of variation, revision suggestions) was provided in an integrated format without linking individual opinions to specific experts. Each expert independently revised their ratings based on this summary information, without knowing how others had responded.

#### 3.2.3 Expert authority coefficient and degree of expert agreement

In this study, the familiarity coefficients for the two rounds were 0.89 and 0.82, respectively, while the judgment criteria coefficients were 0.96 and 0.95. The corresponding expert authority coefficients were calculated as 0.93 and 0.90. Individual authority coefficients across both rounds ranged from 0.85 to 1 (see Table 1). Generally, an expert authority coefficient greater than 0.7 is considered acceptable, indicating that the level of expertise in this study was high.

**Table 1:** Expert authority coefficient and degree of expert agreement.

Expert	First round				Second round			
	Cs	Ca	Cr	Kendall’s W	Cs	Ca	Cr	Kendall’s W
1	0.8	1	0.9	0.187 (X^2^ = 209.598, *P*<0.001)	1.0	0.9	0.95	0.232 (X^2^ = 313.055, *P*<0.001)
2	1	1	1		1	1	1	
3	1	1	1		1	1	1	
4	1	1	1		0.8	1	0.9	
5	0.8	1	0.9		0.8	1	0.9	
6	0.8	0.9	0.85		0.8	0.9	0.85	
7	1	1	1		1	1	1	
8	0.8	0.9	0.85		0.8	0.9	0.85	
9	0.8	0.9	0.85		0.8	0.9	0.85	
10	0.8	1	0.9		0.8	1	0.9	
11	1	1	1		0.8	0.8	0.8	
12	0.8	0.8	0.8		0.8	0.9	0.85	
13	1	1	1		0.8	1	0.9	
均数	0.89	0.96	0.93		0.82	0.95	0.90	

Familiarity (Cs)|Judgment Basis (Ca)|Authority Coefficient (Cr).

Kendall’s W reflects the degree of consensus among experts regarding their evaluations across three dimensions, with a normal range of 0 to 1. The W values for the two rounds were 0.187 (χ^2^ = 209.598) and 0.232 (χ^2^ = 313.055) (Table 1), both showing statistical significance after testing (*p* < 0.05), The results indicate a trend toward consensus among experts, although at a moderate-to-low level. This may be attributed to the inclusion of critical care specialists from various subspecialties, who naturally exhibit differing perspectives on the importance of certain non-core follow-up items—such as those related to social support. Such variation is consistent with the objective nature of developing multidimensional clinical tools.

#### 3.2.4 Concentration of expert opinions and consultation results

(1) After integrating literature evidence, clinical realities, and first-round expert revisions, the research team discussed and modified items as follows (see Supplementary Tables S1, S2):

Variation coefficients for primary and secondary indicators ranged from 0.056–0.145 and 0.077–0.216, respectively, both below 0.25, with full-score ratios exceeding 20%. For tertiary indicators, coefficients varied between 0 and 0.291, where eight items exceeded 0.25: “pleural/peritoneal effusion,” “urinary symptoms (frequency, urgency, dysuria),” “hematuria/proteinuria,” “abnormal urine volume,” “psychological guidance based on causes,” “nutritional status,” “social withdrawal toward colleagues/friends,” and “status decline due to illness.”

Revisions/Deletions: Team discussions led to removal of “pleural/peritoneal effusion,” “hematuria/proteinuria,” and “nutritional status.” Language refinements included: renaming “urinary symptoms” → “manifestations like frequency, urgency, dysuria”; updating “abnormal urine volume” → “urine volume/characteristics (e.g., hematuria/proteinuria)”*; expanding psychological support descriptions to specify causes (disease, finance, social/family stress, work); modifying social interaction terms to “reduced communication/distant interactions among colleagues/friends”; retaining original phrasing for “status decline.”

Additions: Two new secondary domains—"Skin Condition” and “Support Plan and Action Steps”—were introduced, along with 15 tertiary items such as “medical adherence,” “weight changes,” “cognitive screening,” and “social avoidance/stigma.”

(2) Following the second round of the Delphi expert consultation, revisions were made to several items based on the experts’ importance ratings and qualitative feedback. The results are detailed below (Supplementary File; Supplementary Tables S3, S4):

The coefficients of variation (CV) for the first-level, second-level, and third-level indicators ranged from 0 to 0.148, 0 to 0.197, and 0 to 0.220, respectively, all below the threshold of 0.25. Furthermore, the full-score rates for all indicators exceeded 20%. Consequently, no items were eliminated based on these metrics.

Some experts identified redundancy and overlap among certain third-level indicators, proposing their consolidation for simplification. For instance, “Bleeding and local hematoma formation” and “Poor wound healing” were combined into “Poor wound healing (bleeding or local hematoma).” Likewise, “Signs of distal limb ischemia (pain, paresthesia, pallor, pulselessness, paralysis, temperature change)” and “Compartment syndrome” were consolidated into “Limb ischemia or compartment syndrome (pain, paresthesia, pallor, pulselessness, paralysis, temperature change).”

Some experts opined that certain items were inappropriate for follow-up as they could not be self-assessed by patients or their families, and thus recommended their deletion. The deleted items included “Vascular injury (dissection, pseudoaneurysm, arteriovenous fistula)” and “Thrombosis (arterial, venous).”

Four new third-level indicators were incorporated: “Impact on sexual life and intimate relationships,” “Psychological counseling outpatient services,” “Kidney/heart/lung transplantation,” and the “Psychological crisis intervention hotline 12,356.”

Linguistic revisions: Within the “Psychological Status” section, “Coping strategies” was amended to “Psychological intervention and support,” and “Blood glucose” was revised to “Blood glucose (history of diabetes).”

(3) The content validity of the follow-up checklist was assessed using expert evaluation. A 4-point Likert scale was employed to rate the relevance of each item in the checklist to its corresponding dimension, ranging from “not relevant” (1 point) to “highly relevant” (4 points). The item-level Content Validity Index (I-CVI) and the scale-level Content Validity Index/Universal Agreement (S-CVI/UA) were calculated. When there are six or more experts, an I-CVI ≥ 0.780, S-CVI/UA ≥ 0.800, and S-CVI/Ave ≥ 0.900 indicate good content validity (18). In this study, all items rated by the 13 consulted experts were scored as either “moderately relevant” (3 points) or “highly relevant” (4 points). Therefore, the I-CVI for each item was 1.0 (> 0.78), and the S-CVI/UA was also 1.0 (> 0.80). Additionally, the S-CVI/Ave was calculated to be 1.0. Overall, these results demonstrate excellent content validity for the questionnaire.
(4) The resulting Post-Discharge Care and Follow-up Checklist for Adult ECMO Patients consists of a hierarchical structure with 4 primary domains (Physiological Status, Psychological Status, Life Status, and Social and Family Support), which are further divided into 20 secondary items and 83 tertiary items. A detailed breakdown is provided in Table 2. Additionally, based on the core clinical logic of post-ECMO follow-up, we applied three key selection criteria—high urgency, strong prognostic relevance, and high specificity (i.e., complications unique to ECMO)—to re-screen the original 83 tertiary-level items. These were further aligned with clinical priorities requiring priority assessment and mandatory documentation. As a result, we identified 52 core tertiary-level items, while the remaining 31 are categorized as non-core/optional for comprehensive or situational use.
(5) Operational Context (Preliminary recommendation): This checklist is designed as an adaptive tool suitable for multiple scenarios. It adopts a hybrid follow-up model (including telephone, outpatient, and home visits) to meet diverse clinical needs. The application scenarios and recommendations for item adaptability are as follows:

**Table 2:** Nursing follow-up checklist for adult patients discharged after ECMO therapy.

Primary domains	Secondary items	Tertiary items	Data source
1. Physiological status	1. General status	1. Vital signs (body temperature, heart rate, blood pressure, blood oxygen saturation, breathing)*	Outpatient/Home visit: Nurse observation + instrumental measurement; Telephone: Patient self-report/Caregiver proxy report
		2. Blood glucose (for patients with a history of diabetes)*	Outpatient/Home visit: Instrumental measurement; Telephone: Patient self-report of recent monitoring results
		3. Weight changes	Outpatient/Home visit: Scale measurement; Telephone: Patient self-report
	2. Motor function and musculoskeletal system	1. 6-min walk test*	Outpatient: Clinical objective examination; Telephone: Patient self-report of recent test results; Home visit: Not applicable
		2. Cardiopulmonary exercise test (CPET) or 1-min stand-to-sit test*	Outpatient: Clinical objective examination (CPET); Home visit: Nurse observation + counting (1-min stand-to-sit test); Telephone: Not applicable
		3. Muscle function status (muscle strength and muscle atrophy)*	Outpatient/Home visit: Nurse observation + muscle strength grading assessment; Telephone: Patient self-report of functional feelings
		4. Balance and coordination disorders (unsteady standing, prone to falling, difficulty sitting up, abnormal gait)*	Outpatient/Home visit: Nurse observation + functional test; Telephone: Patient self-report/Caregiver proxy report
		5. Neurological functional impairment (limb pain, numbness, pins and needles sensation or crawling sensation, weakened or absent tendon reflexes)*	Outpatient: Neurological physical examination; Telephone: Patient self-report; Home visit: Nurse simple assessment
		6. Joint spasm, pain, effusion	Outpatient/Home visit: Nurse observation + physical examination; Telephone: Patient self-report
		7. Functional activity disorders (difficulty in climbing stairs, inability to stand, difficulty with squatting, foot drop, bedridden, wheelchair bound, or paralysis)*	Outpatient/Home visit: Nurse observation + functional assessment; Telephone: Patient self-report/Caregiver proxy report
	3. ECMO catheter site and vascular complications	1. Poor wound healing (bleeding or local hematoma)*	Outpatient/Home visit: Nurse observation; Telephone: Patient self-report/Caregiver proxy report
		2. Signs of infection (redness, swelling, heat, pain, purulent secretion, etc.)*	Outpatient: Nurse observation + laboratory examination; Telephone: Patient self-report/Caregiver proxy report; Home visit: Nurse observation
		3. Limb swelling (leg circumference, arm circumference)*	Outpatient/Home visit: Nurse measurement; Telephone: Patient self-report
		4. Limb ischemia or compartment syndrome (pain, paresthesia, pallor, pulselessness, motor impairment, temperature changes)*	Outpatient: Nurse observation + vascular examination; Telephone: Patient self-report; Home visit: Nurse observation
		5. Limb gangrene*	Outpatient: Nurse observation + specialist assessment; Telephone: Patient self-report/Caregiver proxy report; Home visit: Nurse observation
		6. Amputation*	Outpatient: Medical record review + nurse observation; Telephone: Patient self-report/Caregiver proxy report; Home visit: Nurse observation
	4. Sleep status	1. Sleep quality (assessment scale)*	All scenarios: Patient self-report (simplified scale)
		2. Sleep guidance (when sleep quality is poor)	All scenarios: Nurse provides guidance based on patient self-report of sleep status
		3. Psychological clinic	All scenarios: Nurse triggers referral based on patient self-report of persistent poor sleep quality
	5. Respiratory system	1. Chronic cough and expectoration*	All scenarios: Patient self-report
		2. Chest tightness, chest pain, or chest discomfort*	All scenarios: Patient self-report
		3. Difficulty breathing (wheezing, shortness of breath)*	Outpatient: Nurse observation + pulmonary function test; Telephone: Patient self-report; Home visit: Nurse observation
		4. Oxygen therapy, invasive/non-invasive respiratory support*	Outpatient/Home visit: Nurse observation + medical record review; Telephone: Patient self-report
		5. Lung transplantation	Outpatient: Medical record review + specialist assessment; Telephone: Patient self-report/Caregiver proxy report; Home visit: Not applicable
	6. Circulatory system	1. Palpitations, palpitation*	All scenarios: Patient self-report
		2. Arrhythmias and conduction disorders (tachycardia or bradycardia)*	Outpatient: Electrocardiogram/Dynamic electrocardiogram; Telephone: Patient self-report of symptoms; Home visit: Not applicable
		3. Cardiac insufficiency (limb edema, fatigue, orthopnea, exertional dyspnea)*	Outpatient/Home visit: Nurse observation + cardiac function assessment; Telephone: Patient self-report/Caregiver proxy report
		4. Use of cardiovascular drugs (antiarrhythmic, cardiac tonic, pressor, antihypertensive, anticoagulant, etc.)*	All scenarios: Patient self-report + medical record review
		5. Mechanical circulatory support	Outpatient: Medical record review + specialist assessment; Telephone: Patient self-report/Caregiver proxy report; Home visit: Not applicable
		6. Heart transplantation	Outpatient: Medical record review + specialist assessment; Telephone: Patient self-report/Caregiver proxy report; Home visit: Not applicable
	7. Nervous system	1. Dizziness, headache*	All scenarios: Patient self-report
		2. Seizures, epilepsy	Outpatient: Neurological physical examination + electroencephalogram; Telephone: Caregiver proxy report; Home visit: Nurse observation + Caregiver proxy report
		2. Consciousness and level of arousal*	Outpatient/Home visit: Nurse observation; Telephone: Caregiver proxy report
		3. Cognitive function (screening for post-ICU syndrome)*	Outpatient: MoCA scale assessment; Telephone/Home visit: Simplified cognitive questionnaire
		4. Neurological functional deficits (motor, sensory, speech, visual, swallowing)*	Outpatient: Neurological physical examination; Telephone: Patient self-report/Caregiver proxy report; Home visit: Nurse simple assessment
		5. Intracranial hemorrhage, infarction (ataxia, hemiplegia, etc.)*	Outpatient: Neuroimaging examination + specialist assessment; Telephone: Patient self-report/Caregiver proxy report; Home visit: Not applicable
		6. Neurological drug therapy/surgery/neurological rehabilitation	All scenarios: Nurse triggers referral based on assessment results; Medical record review for treatment implementation
	8. Digestive system	1. Nutritional risk assessment	Outpatient/Home visit: Nurse assessment via standardized scale; Telephone: Simplified questionnaire assessment
		2. Digestive intolerance (early satiety, nausea, vomiting, anorexia, diarrhea, etc.)*	All scenarios: Patient self-report
		3. Gastric dysmotility (delayed gastric emptying, gastric stasis)	All scenarios: Patient self-report; Outpatient: Specialist assessment + relevant examinations
		4. Intestinal dysmotility (abdominal distension, abdominal pain, constipation, intestinal obstruction, etc.)	All scenarios: Patient self-report; Outpatient: Specialist assessment + relevant examinations
		5. Gastrointestinal ulcer/bleeding (quantity, consistency, color of vomit and stool)*	All scenarios: Patient self-report/Caregiver proxy report; Outpatient: Laboratory examination + specialist assessment
	9. Urinary system	1. Frequent, urgent, and painful urination*	All scenarios: Patient self-report
		2. Urine output and characteristics (hematuria, proteinuria)*	Outpatient: Nurse observation + urine routine examination; Telephone: Patient self-report; Home visit: Patient self-report/Caregiver proxy report
		3. Use of diuretic medications*	All scenarios: Patient self-report + medical record review
		4. Receiving blood purification therapy	Outpatient: Medical record review + nurse observation; Telephone: Patient self-report; Home visit: Not applicable
		5. Kidney transplantation	Outpatient: Medical record review + specialist assessment; Telephone: Patient self-report/Caregiver proxy report; Home visit: Not applicable
	10. Skin condition	1. Skin jaundice, petechiae, ecchymoses*	Outpatient/Home visit: Nurse observation; Telephone: Patient self-report/Caregiver proxy report
		2. Skin mottling, cyanosis, ischemic necrosis of skin tissue in distal limbs	Outpatient/Home visit: Nurse observation; Telephone: Patient self-report/Caregiver proxy report
		3. Skin integrity (pressure injuries, scars at catheter sites)*	Outpatient/Home visit: Nurse observation; Telephone: Patient self-report/Caregiver proxy report
		4. Incontinence-associated dermatitis (area, grade)	Outpatient/Home visit: Nurse observation and grading; Telephone: Patient self-report/Caregiver proxy report
2. Psychological status	1. Problems encountered	1. Anxiety (Hospital Anxiety and Depression Scale, HADS)*	All scenarios: Patient self-report (simplified scale)
		2. Depression (Hospital Anxiety and Depression Scale, HADS)*	All scenarios: Patient self-report (simplified scale)
		3. Post-traumatic stress disorder (Post-Traumatic Stress Disorder Screening Scale, PC-PTSD-5)*	All scenarios: Patient self-report (simplified scale)
		4. Other mental health issues	All scenarios: Patient self-report; Outpatient: Psychiatric specialist assessment
	2. Coping strategies/interventions	1. Receive psychological counseling based on different reasons (disease, economy, family, society, work)*	All scenarios: Nurse triggers referral based on patient self-report of psychological problems
		2. Access to psychological consultation outpatient services	All scenarios: Nurse coordinates referral based on patient’s psychological problem severity
		3. Medication treatment	All scenarios: Patient self-report + medical record review; Outpatient: Psychiatrist prescription and follow-up
		4. Psychological crisis intervention hotline 12,356	All scenarios: Nurse provides contact information based on patient’s psychological status
	3. Perceived value of ECMO treatment	1. Value perception and outcome evaluation	All scenarios: Patient self-report
		2. Participation and recognition in ECMO decision-making	All scenarios: Patient self-report
		3. Trust and evaluation of the medical team	All scenarios: Patient self-report
3. Life status	1. Activities of daily living	1. Self-care ability*	Outpatient/Home visit: Barthel Index assessment; Telephone: Patient self-report/Caregiver proxy report
		2. Quality of life (Health Survey Short Form, SF-36)	Outpatient: Scale assessment by medical staff; Telephone/Home visit: Patient self-report via simplified scale
		3. Rehabilitation training*	All scenarios: Patient self-report (compliance, frequency, effect) + Nurse assessment
	2. Employment/work status	1. Changes in work ability (whether to return to the job and changes in work efficiency)*	All scenarios: Patient self-report
	3. Medical adherence	1. Lifestyle changes (dietary habits, smoking cessation, alcohol cessation, etc.)	All scenarios: Patient self-report
		2. Medication adherence*	All scenarios: Patient self-report
		3. Follow-up adherence*	All scenarios: Patient self-report + medical record review
	4. Impact on sexual life and intimate relationships	1. Physical function and body perception (changes in libido, physiological responses, physical limitations)	All scenarios: Patient self-report (voluntary)
		2. Partnership and emotional connection (the impact of the disease on interactions between partners and emotional bonds)	All scenarios: Patient self-report (voluntary)
4. Social and Family Support	1. Family support system	1. Need for fixed/rotating family caregivers*	All scenarios: Patient self-report/Caregiver proxy report
		2. Change in family economic income compared to before	All scenarios: Patient/Caregiver self-report
		3. Impact of the disease on family finances*	All scenarios: Patient/Caregiver self-report
		4. Impact on caregivers (physical, psychological, social)	All scenarios: Caregiver self-report
	2. Social interaction and networking	1. Emotional estrangement from relatives and friends	All scenarios: Patient self-report
		2. Decreased frequency of interaction/communication with colleagues (students) or friends or a distant interaction	All scenarios: Patient self-report
		3. Social avoidance and stigma	All scenarios: Patient self-report
		4. Sense of social belonging and value	All scenarios: Patient self-report
		5. Social status decline due to the disease	All scenarios: Patient self-report
	3. Support plan and action steps	1. Public welfare rehabilitation support (physical, psychological)	All scenarios: Nurse provides resource information + Patient self-report of access status
		2. Employment guidance and support	All scenarios: Nurse links resources + Patient self-report of demand and access status
		3. Caregiver support (caregiving skills, own psychological and physical health)	All scenarios: Nurse provides training/guidance + Caregiver self-report of needs
		4. Application for special medical benefits after ECMO treatment	All scenarios: Nurse provides application guidance + Patient self-report of application progress/result

* Core follow-up items.

Telephone follow-up: Primarily used for routine follow-up (e.g., 1 week, 6 months, 12 months post-discharge), focusing on core items and patient self-report/simplified assessment items. For objective examination items such as the 6-min walk test, patient self-report of recent test results (e.g., “whether the test was completed and results were normal”) is adopted as a substitute.

Outpatient follow-up: Recommended for comprehensive assessment (e.g., 1 month, 3 months post-discharge), allowing completion of all items including objective tests (e.g., neuroimaging, pulmonary function test) and physical examination components.

Home visit: Targeted at bedridden or mobility-limited patients, focusing on physiological status and activities of daily living. For physical assessment items such as limb swelling or wound healing, nurses guide patients/families to perform simple self-assessments (e.g., measuring leg circumference, observing wound redness/swelling/exudate). Items requiring objective testing (e.g., 6-min walk test, neuroimaging, cardiopulmonary exercise test) are defined as “conditional assessment items” rather than routine screening elements. They are only recommended for patients with specific clinical indications (e.g., suspected neurological complications for neuroimaging) or in applicable follow-up scenarios (e.g., outpatient follow-up for cardiopulmonary exercise test), avoiding unnecessary assessment burdens.

### 3.3 Preliminary testing results of the nursing follow-up checklist for adult patients discharged after ECMO therapy

In this study, nurses involved in the follow-up received structured training, which included theoretical instruction (on the background, item definitions, and usage guidelines of the checklist), case-based practice, and a 0.5-h practical assessment to ensure consistent understanding. An instant communication mechanism was also established to promptly address any operational questions raised by the nurses during implementation.

The six follow-up nurses had 8–20 years of clinical experience (mean: 12 ± 4.38 years) and were aged 31–44 years (mean: 35 ± 4.86). The mean age of the 10 followed-up patients was 46.2 ± 13.0 years. Other baseline characteristics of the followed-up patients are presented in Table 3. With each session lasting 30–40 min. During follow-ups, completion rate was 100%, number of missing items was 0, average completion time was 35.2 ± 4.1 min, number of items perceived as unclear by nurses was 0. All participating nurses acknowledged its professional relevance and practical utility, noting it addressed all essential needs and concerns of adult ECMO survivors. They deemed the allocated timeframe reasonable and proposed no further modifications. A validated checklist assessment questionnaire (7 dimensions, Likert 5-point scale, 1 = strongly disagree, 5 = strongly agree; total score range: 35 points) was used to evaluate nurses’ perceptions of the clarity, organization, practicality, and comprehensiveness of the checklist. The overall mean score was 30.83 ± 2.93. As shown in Table 4, the average scores for all individual dimensions exceeded 4, indicating high ratings across all aspects. These results suggest that nurses regard the checklist demonstrated good feasibility and usability in single-center telephone follow-up settings, with high acceptance among both nurses and patients, demonstrating good face validity.

**Table 3:** Baseline characteristics of followed-up patients.

Patient No.	Sex	Age (years)	ECMO indication	ECMO mode	ECMO duration (h)
1	Male	49	Infective endocarditis	VA → VA-V	138.99
2	Male	57	Cardiac and respiratory arrest	VA	216.02
3	Female	31	Postpartum hemorrhage, cardiac arrest	VA	15.98
4	Female	46	Anaphylactic shock, cardiac arrest	VA	57
5	Male	45	Myocardial infarction	VA	39.5
6	Male	35	Fulminant myocarditis	VA	93
7	Male	40	Myocardial infarction, cardiogenic shock	VA	65.5
8	Male	63	Pulmonary infection	VV	305.5
9	Female	29	Severe pneumonia, cardiac arrest	VA → VA-V	189.5
10	Male	67	Severe pneumonia	VV	187

**Table 4:** Nurses’ overall evaluation of the checklist’s usability.

Evaluation dimensions	Average score ± Standard deviation
Clarity of items	4.33 ± 0.49
Reasonableness of usage burden	4.17 ± 0.75
Content relevance	4.50 ± 0.52
Structural organization	4.42 ± 0.51
Content comprehensiveness	4.67 ± 0.49
Clinical practicality	4.33 ± 0.49
Clinical helpfulness	4.25 ± 0.62

To evaluate inter-rater reliability, two nurses with rich clinical experience in ECMO nursing were selected as raters. They received standardized training on the checklist to ensure consistent understanding. The raters independently evaluated 5 out of 10 follow-up patients at the item-level. No communication was allowed between raters during the evaluation process.

The Kappa coefficient was chosen to assess inter-rater reliability because the evaluation results of checklist items were categorical data. According to statistical standards, a Kappa value >0.75 indicates good consistency, which is suitable for evaluating the reliability of clinical assessment tools. The results showed a Kappa value of 0.82 (*p* < 0.001), indicating good inter-rater reliability. It should be noted that this inter-rater reliability analysis is a preliminary assessment based on a single-center small sample. Subsequent multicenter studies with larger sample sizes will further verify test–retest reliability and inter-rater reliability across different institutions to enhance the tool’s measurement stability.

## 4 Discussion

This study addressed the clinical challenge of lacking standardized tools for transitional care in adult patients discharged after ECMO therapy. By employing a pathway that deeply integrates evidence-based methodology with clinical practice—through literature review, semi-structured interviews, Delphi expert consultation, and preliminary clinical testing—we ultimately developed a nursing follow-up checklist encompassing four primary domains: physiological status, psychological status, living conditions, and social and family support. The entire research process followed scientific procedures for tool development, including evidence-based literature review, semi-structured interviews, two rounds of Delphi expert consultation, and preliminary clinical testing, which is consistent with the methodological framework of similar nursing tool development studies (19, 20), with methodological design and domain selection closely aligned with long-term management needs of post-ECMO patients.

### 4.1 The constructed nursing follow-up checklist for adult ECMO postoperative discharge patients is scientific and reliable

This study strictly follows the key development steps of “evidence-based, interview, expert argumentation”. Firstly, it systematically retrieves multiple databases and guideline platforms, including 39 high-quality documents (including guidelines, systematic reviews, cohort studies, etc.), to ensure that the core items of the checklist conform to the evidence-based evidence for long-term management of ECMO postoperative patients. Secondly, it supplements the core needs from the patient’s perspective through semi-structured interviews, such as psychological support and social function recovery, compensating for the limitation of literature sorting which covers insufficient “patient subjective experience”. Finally, it relies on the Delphi method to complete two rounds of expert consultation. The active coefficients of the two rounds of consultation were 86.67 and 100.00%, respectively. The authority coefficient was ≥0.90, and the Kendall’s W coefficients were 0.187 and 0.232 (*p* < 0.001), indicating that the enthusiasm, authority, and coordination of experts are at a high level, meeting the core quality requirements of the Delphi method and laying a methodological foundation for the scientific nature of the content of the checklist. Its construction process is consistent with that of Wang Ting (19) and Han (20), among others, in constructing nursing follow-up checklists, and has achieved good results.

In this study, the core screening criteria were “assigned mean > 3.50, coefficient of variation < 0.25, and full score ratio > 20%.” Based on expert opinions and clinical feasibility, item optimization was conducted: In the first round of consultation, items with a coefficient of variation exceeding the standard such as “pleural effusion, ascites,” and “nutritional status” were deleted to avoid redundancy in non-core follow-up dimensions after ECMO therapy; The expression of items such as “abnormal urination” and “social coldness” was revised to make it more consistent with the popularization and accuracy of clinical records; New secondary items such as “skin condition” and “support scheme and action plan,” and tertiary items such as “medical compliance” and “cognitive function screening” were added to supplement dimensions that are easily overlooked after ECMO therapy, such as skin complications and long-term rehabilitation support. After the second round of consultation, the coefficient of variation of all items was <0.25, and the full score ratio was >20%, indicating that the item setting has reached expert consensus and is highly consistent with the clinical needs of multisystem complication management and long-term rehabilitation of patients after ECMO therapy.

However, unlike standardized measurement scales, this is positioned as a “this follow-up checklist is positioned as a “clinical data recording tool.” Therefore, it did not conduct validity tests such as construct validity and criterion-related validity, which are typical for scale tools. Instead, content validity was verified through expert consultations, and interrater reliability was preliminarily tested. Flottorp et al. (21) proposed that a follow-up list is a follow-up tool designed for the characteristics of patients with different diseases, and has applicability, logic, and clarity. It can standardize the follow-up process and solve problems such as unclear organization and insufficient systematicness during the follow-up (22). Therefore, this study is consistent with the functional orientation of the follow-up list—its core value lies in “comprehensively and normatively recording the multidimensional status of patients after discharge,” rather than “measuring an abstract concept.” In preliminary clinical tests, 6 nurses and 10 patients fed back that the checklist content was comprehensive and the follow-up duration was reasonable, further verifying the operability and clinical adaptability of the checklist.

Although traditional construct validity is not the focus of this study, the systematic assessment of content validity (based on evidence and expert consensus) ensures that the checklist covers all core domains of post-ECMO follow-up. The feasibility and acceptability verified by pre-testing confirm its applicability in clinical practice. Subsequent studies will focus on the measurement properties such as inter-rater reliability, test–retest reliability, and responsiveness to further improve the tool’s measurement performance and promote its clinical popularization.

### 4.2 The content of the constructed nursing follow-up checklist for adult ECMO postoperative discharge patients is comprehensive

The comprehensiveness of the checklist is reflected in its close alignment with the core needs of long-term rehabilitation of ECMO postoperative patients, and on this basis, the tool has novel contributions that fill the gaps in the current clinical follow-up of ECMO survivors, which is also the key to its differentiation from other general follow-up tools. On the basis of covering traditional physiological monitoring dimensions, it further expands into the fields of psychology, life, and social family support according to patient interview results and literature evidence-based approach, constructing an “bio-psycho-social” integrated follow-up system, fully reflecting clinical relevance and practical application. In terms of the physiological status dimension, this study fully combines the invasive characteristics of ECMO technology and the risk of multiple system complications after surgery, refining it into 10 secondary items. It not only focuses on the monitoring of specific complications after ECMO therapy but also covers basic physiological indicators and rehabilitation assessment, forming a complete chain of physiological follow-up. For the risk of vascular complications related to ECMO catheterization, entries such as “poor wound healing (bleeding or local hematoma)” and “limb ischemia or compartment syndrome” are set up, which are consistent with the high incidence of catheterization long-term risks reported in the literature (23, 24); For the long-term impact on the nervous system, items such as “cognitive function (screening for post-ICU syndrome)” and “neurological functional defects (motor, sensory, speech)” are included, echoing the conclusion in von Bahr et al.’s study that “patients after ECMO therapy are prone to cognitive disorders” (25); At the same time, through “6-min walk test” and “cardiopulmonary function combined test,” quantitative evaluation of rehabilitation outcomes is realized, solving the limitation of “emphasizing symptom monitoring while overlooking rehabilitation progress” in traditional follow-up, providing a basis for early identification of hidden physiological problems and timely adjustment of intervention plans.

The design of the psychological state dimension breaks through the single mode of “only recording symptoms without intervention guidance” in traditional follow-up, constructing a closed-loop management framework of “problem identification—intervention implementation—subjective experience,” which fully conforms to the high psychological risk clinical characteristics of ECMO postoperative patients. Existing studies show that the incidence of anxiety, depression, and post-traumatic stress disorder in ECMO postoperative patients reaches 30–50%, and psychological state directly affects rehabilitation compliance and long-term quality of life (13, 26, 27). On the basis of clearly listing core psychological problems such as “anxiety,” “depression,” and “post-traumatic stress disorder,” this checklist further refines targeted intervention measures, such as “providing psychological counseling based on different causes such as disease, economy, and family,” “connecting with psychological consultation outpatient services,” and “providing psychological crisis intervention hotline 12,356,” providing directly operable intervention paths for clinical practice, avoiding vague expressions like “psychological support is recommended”; At the same time, it innovatively includes the dimension of “recognition of ECMO treatment,” covering patients’ subjective experience such as perception of treatment value, sense of decision participation, and trust in medical teams, echoing Chen et al. qualitative research (12) that “treatment recognition affects the psychological state of ECMO postoperative patients” (28), providing a basis for individualized psychological intervention.

The design of the social and family support dimensions in this study revolves around the holistic nursing philosophy of “patient-centeredness,” focusing on the core needs of ECMO postoperative patients to return to society and family, and making up for the lack of attention to social functions and family support in previous follow-up tools. In the life status dimension, the study breaks through the single evaluation of “daily living ability” in tradition, adding new items such as “impact of sexual life and intimate relationships” (covering physical function, partner emotional connection) and “medical compliance” (lifestyle, medication, review). The former is rarely mentioned in previous ECMO follow-up tools but is an important component for patients to return to normal family life, while the latter is directly related to long-term prognosis (such as poor compliance with anticoagulants can easily lead to an increased risk of thrombosis), both being key links urgently needed in clinical practice. In the social and family support dimension, this study comprehensively covers the actual difficulties faced by patients after discharge from three levels: “family support system”, “social interaction”, and “support plan”: For the family level, items such as “caregiver needs” and “family economic impact” are set up, echoing the conclusion in Spangenberg et al.’s research that “family caregiver burden is an important influencing factor for the rehabilitation of ECMO postoperative patients” (11); For the social level, items such as “social avoidance and stigma” and “social belonging and sense of value” are included, paying attention to psychological barriers in the process of rebuilding social functions; Regarding the connection of support resources, items such as “public welfare rehabilitation support” and “employment guidance” are set up, providing external resource link paths for long-term rehabilitation for patients, truly achieving comprehensive follow-up management from “physical rehabilitation” to “return to social roles”.

### 4.3 The clinical application value of the constructed nursing follow-up checklist for adult ECMO postoperative discharge patients

The nursing follow-up checklist for adult ECMO postoperative discharge patients constructed in this study may provide multidimensional support for the standardized development of ECMO postoperative continuing care at the clinical application level and can provide multidimensional support for the standardized development of ECMO postoperative continuing care. Firstly, the checklist can effectively standardize the follow-up process, solving the problem that ECMO postoperative follow-up currently relies on individual nurse experience with large content arbitrariness—through a hierarchical framework of 4 primary items, 20 secondary items, and 83 tertiary items, it guides nurses to systematically collect patient information according to dimensions, ensuring that the follow-up content covers core areas such as physical condition, psychological state, life status, and social family support, avoiding omissions or deviation of focus, and improving the standardization level of follow-up work. Secondly, the checklist constructed in this study can serve as a communication carrier for multidisciplinary collaboration, promoting the transformation of ECMO postoperative continuing care from “single department follow-up” to “multidisciplinary linkage intervention”. For example, after nurses record the patient’s cognitive impairment performance through the checklist, they can directly connect with the rehabilitation department for targeted training; When psychosocial problems are identified, professional intervention can be quickly integrated with the psychology department, promoting the collaborative cooperation of multidisciplinary teams such as critical care medicine, rehabilitation, and psychology. In addition, the multidimensional follow-up data recorded by the checklist can provide real-world evidence for subsequent ECMO postoperative continuing care intervention studies. By analyzing data such as the incidence of physiological complications, types of psychosocial problems, and deficiencies in social support, key influencing factors for long-term rehabilitation of patients can be identified, providing a scientific basis for formulating “individualized intervention plans” (such as group psychological counseling for patients with social avoidance, skill training for caregivers), ultimately helping to improve the long-term survival rate and quality of life of ECMO postoperative patients.

We recommend that the checklist be used longitudinally at standardized time points: 1 week post-discharge: A core item screening via telephone to screen for acute complications. 1 month post-discharge: A comprehensive assessment (outpatient or home-based) using the full checklist. 3 months post-discharge: An in-depth, multi-system follow-up at this 90-day critical threshold to assess recovery and complications, which guides adjustments to subsequent follow-up frequency. 6 and 12 months post-discharge: Routine follow-ups with core items. For clinically stable patients, the interval can be extended. The 90-day critical threshold for long-term survival after ECMO proposed by von Bahr et al. (8) is considered a key juncture. We believe it can serve as a crucial point for evaluating long-term survival potential, identifying unmet rehabilitation needs, and developing personalized follow-up plans (e.g., shortening intervals for patients with poor recovery, extending them for those who are stable), thereby enabling targeted, continuous care.

### 4.4 Limitation

Despite the advantages in methodological design and clinical relevance, this study has certain limitations. First, the regional representativeness of the expert consultation was insufficient. Although the 13 experts covered five provinces, the proportion from Hubei was disproportionately high, and opinions from regions with limited medical resources, such as the western and northeastern areas, were lacking. This may affect the applicability of the checklist in medical institutions of different levels. In subsequent studies, we will expand the geographical scope of experts to include specialists from grassroots and rehabilitation institutions, thereby enhancing the universality of the tool. Second, the sample size and scope of the clinical validation were limited. The validation involved only 10 patients and 6 nurses at a single center, and focused solely on telephone follow-up, excluding outpatient and home-based follow-up scenarios. Furthermore, inter-rater reliability and test–retest reliability were not quantitatively evaluated. Future work will involve multi-center, large-sample studies to conduct systematic validation.

Moreover, the study participants were all recruited from a Grade III A hospital in Wuhan, where ECMO technology is mature and follow-up procedures are standardized. Consequently, the applicability of the checklist in grassroots or resource-limited institutions remains unverified. Furthermore, convenience sampling was adopted, which may introduce selection bias and limit the generalizability of the results. Meanwhile, this study only evaluated process indicators such as subjective satisfaction and operational feasibility; objective outcome data, such as complication rates and readmission rates, were not collected. Therefore, the actual impact of the checklist on patient prognosis could not be verified.

## 5 Conclusion

The nursing follow-up checklist for adult post-ECMO discharge patients developed in this study comprises 4 primary indicators (physiological status, psychological status, living conditions, and social and family support), 20 secondary indicators, and 83 tertiary indicators. Developed based on evidence-based practice and expert consensus, the checklist has undergone content validity verification and feasibility testing. It can meet clinical follow-up needs and serve as a standardized recording tool for post-ECMO discharge patients.

## Data Availability

The raw data supporting the conclusions of this article will be made available by the authors, without undue reservation.
