# Supplementary material for: Development of a nursing follow-up checklist for adult ECMO-treated discharged patients: a Delphi consensus and feasibility study
Source: Front Med (Lausanne). 2026 Mar 25;13:1779603. doi: 10.3389/fmed.2026.1779603 (PMC13057531; doi:10.3389/fmed.2026.1779603)
Supplement: Supplementary file 1 [file Table_1.DOCX]

**Constructing a nursing follow-up checklist for adult ECMO postoperative discharge patients**

Search terms: "extracorporeal membrane oxygenation/ECMO", "rehabilitation/follow-up/callback/family/household/home/out of-hospital/after discharge/continuing nursing/ continuing care/transitional care/quality of life"

**CNKI：12**

SU=(‘Extracorporeal Membrane Oxygenation’+‘ECMO’) AND SU=(‘Follow-up’+‘Revisit’+‘Out-of-hospital’+‘Home’+‘Post-discharge’+‘Continuing’+‘Quality of Life’+‘Survival Quality’)

**Wanfang：566**

("Extracorporeal Membrane Oxygenation" OR "ECMO") AND ("Revisit" OR "Follow-up" OR "Out-of-hospital" OR "Home" OR "Post-discharge" OR "Continuing" OR "Quality of Life" OR "Survival Quality")

**PubMed：4875**

((extracorporeal membrane oxygenation) OR (ECMO)) AND (((((rehabilitation) OR (follow up)) OR (callback)) OR ("family" or "household" or "home")) OR ("out of-hospital" or "after discharge" or "continuing nursing" or " continuing care" or " transitional care" or "quality of life"))


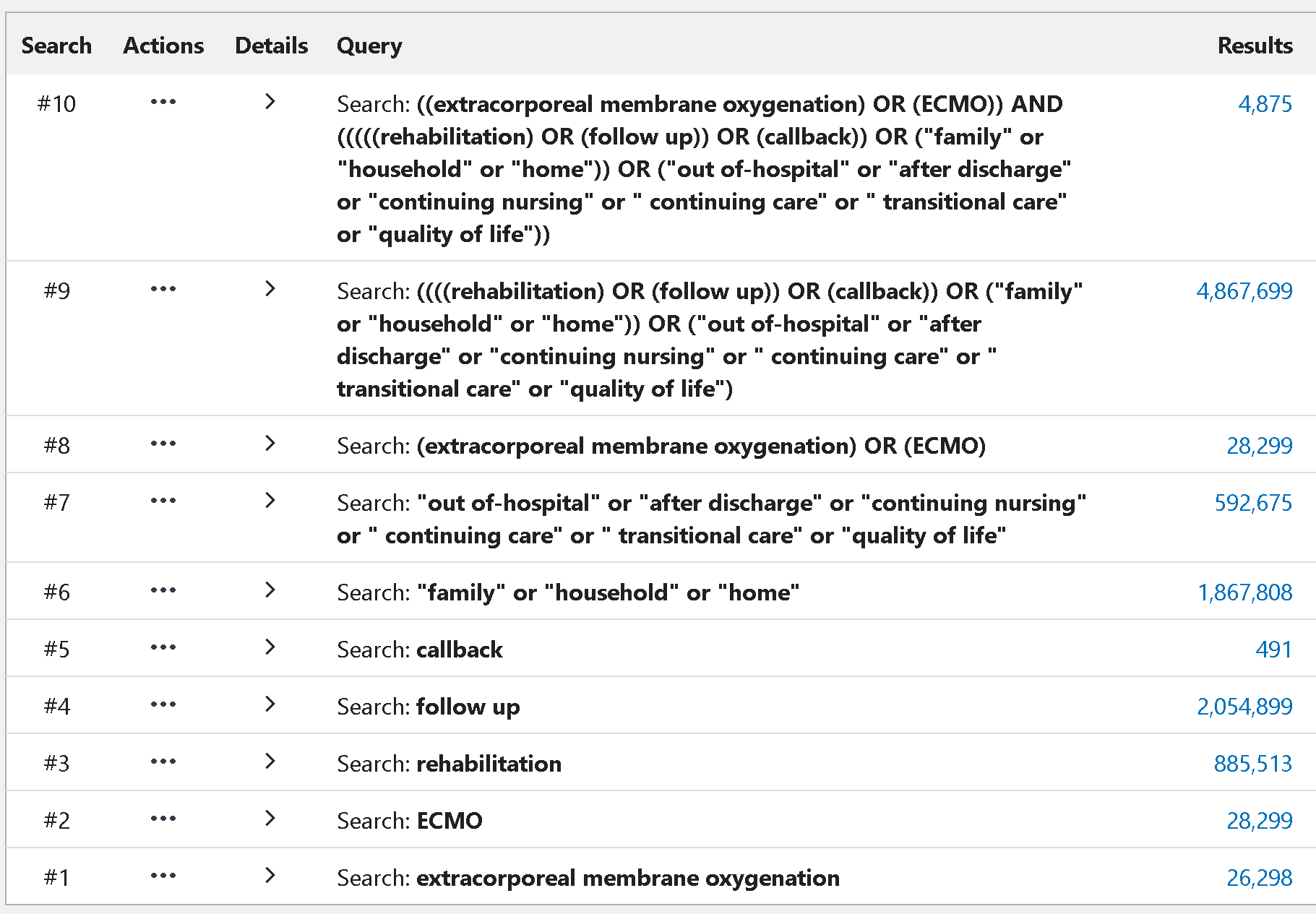


**CINAHL：1194**

(extracorporeal membrane oxygenation or ecmo) AND ((((((rehabilitation) OR (follow up)) OR (callback)) OR ("family" or "household" or "home")) OR ("out of-hospital" or "after discharge" or "continuing nursing" or " continuing care" or " transitional care" or "quality of life")))

**Cochrane Library：457**

(extracorporeal membrane oxygenation or ecmo) AND ((((((rehabilitation) OR (follow up)) OR (callback)) OR ("family" or "household" or "home")) OR ("out of-hospital" or "after discharge" or "continuing nursing" or " continuing care" or " transitional care" or "quality of life")))

**Agency for Healthcare Research and Quality (AHRQ)：49**

extracorporeal membrane oxygenation or ecmo

**Scottish Intercollegiate Guidelines Network (SIGN)：52**

extracorporeal membrane oxygenation or ecmo

**Guidelines International Network (GIN)：1**

extracorporeal membrane oxygenation or ecmo

**National Institute for Health and Care Excellence (NICE)：25**

extracorporeal membrane oxygenation or ecmo

**Medlive Guideline Network：95**

"Extracorporeal Membrane Oxygenation" OR "ECMO"
