# Supplementary material for: Development of a nursing follow-up checklist for adult ECMO-treated discharged patients: a Delphi consensus and feasibility study
Source: Front Med (Lausanne). 2026 Mar 25;13:1779603. doi: 10.3389/fmed.2026.1779603 (PMC13057531; doi:10.3389/fmed.2026.1779603)
Supplement: Supplementary file 2 [file Table_2.DOCX]

**Literature Evaluation**

**Quality Evaluation of Guidelines (n=1)**

| **No.** | **Included Literature** | **Standardized Percentage in Each Field (%)** | | | | | | **Number of Fields with ≥60%** | **Number of Fields with ≤30%** | **Recommendation Level** |
| --- | --- | --- | --- | --- | --- | --- | --- | --- | --- | --- |
|  |  | **Range and Purpose** | **Participants** | **Rigor** | **Clarity** | **Applicability** | **Independence** |  |  |  |
| 1 | Cho SM et al. | 94.44 | 81.25% | 92.86% | 83.33% | 77.78% | 100 | 6 | 0 | A |

1、Cho SM, Hwang J, Chiarini G, Amer M, Antonini MV, Barrett N, Belohlavek J, Brodie D, Dalton HJ, Diaz R, Elhazmi A, Tahsili-Fahadan P, Fanning J, Fraser J, Hoskote A, Jung JS, Lotz C, MacLaren G, Peek G, Polito A, Pudil J, Raman L, Ramanathan K, Dos Reis Miranda D, Rob D, Salazar Rojas L, Taccone FS, Whitman G, Zaaqoq AM, Lorusso R. Neurological monitoring and management for adult extracorporeal membrane oxygenation patients: Extracorporeal Life Support Organization consensus guidelines. Crit Care. 2024 Sep 6;28(1):296. Doi: 10.1186/s13054-024-05082-z. Erratum in: Crit Care. 2024 Oct 7;28(1):327. Doi: 10.1186/s13054-024-05107-7.

**Quality Evaluation of Quasi-Experimental Research Literature (n=1)**

| No. | Included Literature | ① | ② | ③ | ④ | ⑤ | ⑥ | ⑦ | ⑧ | ⑨ | ⑩ | Evaluation Result |
| --- | --- | --- | --- | --- | --- | --- | --- | --- | --- | --- | --- | --- |
| 2 | Chen et al. | Yes | Yes | Unclear | Yes | No | Yes | Yes | Unclear | Yes | Yes | Included |

Note: ① Is the research purpose clear? Is the basis for the topic sufficient? ② Is the process of selecting samples, grouping, inclusion criteria, and exclusion criteria clearly described? ③ Was blinding implemented for the subjects and outcome assessors? ④ Were the baseline characteristics comparable between the experimental and control groups? ⑤ Is the loss of samples described? Are the lost samples also included in the analysis? ⑥ Were the outcome measures assessed using the same methods for all groups (with training for assessors)? ⑦ Were other interventions received by the experimental and control groups the same, apart from the intervention being tested? ⑧ Is there a description of the methods for assessing adverse reactions or side effects? ⑨ Is the establishment of the outcome indicators appropriate? Are the assessment methods reliable? ⑩ Is the data analysis method appropriate?

2、Chen Jinmeng, Lu Kun. Analysis of related factors affecting the quality of life and complications in critically ill survivors after ECMO treatment. Heilongjiang Journal of Chinese Medicine and Pharmacy, 2024, 53(01):123-125.

**Quality Evaluation of Qualitative Research Literature (JBI 2016) (n=3)**

| **No.** | **Included Literature** | **①** | **②** | **③** | **④** | **⑤** | **⑥** | **⑦** | **⑧** | **⑨** | **⑩** | **Evaluation Result** |
| --- | --- | --- | --- | --- | --- | --- | --- | --- | --- | --- | --- | --- |
| 3 | Wang et al | Yes | Yes | Yes | Yes | Yes | Yes | Yes | Yes | Yes | Yes | Included |
| 23 | Chen KH et al | Yes | Yes | Yes | Yes | Yes | Yes | Yes | Yes | Yes | Yes | Included |
| 24 | Wang F et al | Yes | Yes | Yes | Yes | Yes | Yes | Yes | Yes | Yes | Yes | Included |

Note: ① Is there consistency between the philosophical foundation and methodology? ② Is there consistency between the methodology and the research question or objective? ③ Is there consistency between the methodology and data collection methods? ④ Is there consistency between the methodology and the representativeness and typicality of the data, as well as data analysis methods? ⑤ Is there consistency between the methodology and the interpretation of results? ⑥ Is the researcher's own situation explained from the perspective of cultural background and values? ⑦ Is the influence of the researcher on the study or the impact of the study on the researcher explained? ⑧ Are the subject and their views typical? ⑨ Has the study been approved by an ethics committee? ⑩ Is the conclusion derived from the analysis and interpretation of the data?

3、Wang Fengzhen, Zhang Yuhao, Wu Shujing, et al. Qualitative study on the living experience of patients receiving extracorporeal membrane oxygenation therapy after discharge from hospital. Evidence-Based Nursing, 2024, 10(5):942-946.DOI:10.12102/j.issn.2095-8668.2024.05.034.

23、Chen KH, Tsai FC, Tsai CS, Yeh SL, Weng LC, Yeh LC. Problems and health needs of adult extracorporeal membrane oxygenation patients following hospital discharge: A qualitative study. Heart Lung. 2016 Mar-Apr;45(2):147-53. doi: 10.1016/j.hrtlng.2015.12.005.

24、Wang F, Zhang Y, Wu S, Xie H, Lin D, Wen X, Duan Z, Lu Y, Liu Z, Hu S, Liu J. Post-discharge experiences of patients with extracorporeal membrane oxygenation support: A qualitative study. Perfusion. 2024 Jan;39(1):189-200. doi: 10.1177/02676591221135165.

**Quality Evaluation of Systematic Review Literature (n=5)**

| **No.** | **Included Literature** | **①** | **②** | **③** | **④** | **⑤** | **⑥** | **⑦** | **⑧** | **⑨** | **⑩** | **⑪** | **Evaluation Result** |
| --- | --- | --- | --- | --- | --- | --- | --- | --- | --- | --- | --- | --- | --- |
| 4 | Chen et al | Yes | Yes | Yes | Yes | Yes | Yes | Yes | Yes | Unclear | Yes | Yes | Included |
| 15 | Kurniawati ER et al | Yes | Yes | Yes | Yes | Yes | Yes | Yes | Unclear | Yes | Yes | Yes | Included |
| 30 | Knudson KA et al | Yes | Yes | Yes | Yes | Yes | Yes | Yes | Yes | Yes | Yes | Yes | Included |
| 32 | Turgeon J et al | Yes | Yes | Yes | Yes | Yes | Yes | Yes | Yes | Yes | Yes | Yes | Included |
| 34 | Wilcox ME | Yes | Yes | Yes | Yes | Yes | Yes | Yes | Unclear | Yes | Yes | Yes | Included |

Note: ① Is the statement of the systematic review problem clear? ② Are the inclusion criteria appropriate for the problem of the systematic review? ③ Is the search strategy appropriate? ④ Are the sources of the retrieved literature such as databases clear and appropriate? ⑤ Is the standard for evaluating literature quality appropriate? ⑥ Were the quality evaluations of the literature independently completed by two or more reviewers? ⑦ Were measures taken to reduce errors during data extraction? ⑧ Is the method for synthesizing/combining studies appropriate? ⑨ Is the possibility of publication bias evaluated? ⑩ Are the policy or practice recommendations based on the results of the systematic review? ⑪ Is a specific directional suggestion made for further research?

4、Chen Yuanyuan, Yao Ruishan, Wan Jia, et al. Meta-integration of qualitative studies on the living experience and needs of patients receiving extracorporeal membrane oxygenation therapy. Journal of Nursing Science, 2024, 39(22):43-47,51.DOI:10.3870/j.issn.1001-4152.2024.22.043.

15、Kurniawati ER, Rutjens VGH, Vranken NPA, Delnoij TSR, Lorusso R, van der Horst ICC, Maessen JG, Weerwind PW. Quality of life following adult veno-venous extracorporeal membrane oxygenation for acute respiratory distress syndrome: a systematic review. Qual Life Res. 2021 Aug;30(8):2123-2135. doi: 10.1007/s11136-021-02834-0.

30、Knudson KA, Gustafson CM, Sadler LS, Whittemore R, Redeker NS, Andrews LK, Mangi A, Funk M. Long-term health-related quality of life of adult patients treated with extracorporeal membrane oxygenation (ECMO): An integrative review. Heart Lung. 2019 Nov-Dec;48(6):538-552. doi: 10.1016/j.hrtlng.2019.08.016.

32、Turgeon J, Venkatamaran V, Englesakis M, Fan E. Long-term outcomes of patients supported with extracorporeal membrane oxygenation for acute respiratory distress syndrome: a systematic review and meta-analysis. Intensive Care Med. 2024 Mar;50(3):350-370. doi: 10.1007/s00134-023-07301-7.

34、Wilcox ME, Jaramillo-Rocha V, Hodgson C, Taglione MS, Ferguson ND, Fan E. Long-Term Quality of Life After Extracorporeal Membrane Oxygenation in ARDS Survivors: Systematic Review and Meta-Analysis. J Intensive Care Med. 2020 Mar;35(3):233-243. doi: 10.1177/0885066617737035

**Quality Evaluation of Randomized Controlled Trial Literature (n=1)**

| **No.** | **Included Literature** | **①** | **②** | **③** | **④** | **⑤** | **⑥** | **Evaluation Result** |
| --- | --- | --- | --- | --- | --- | --- | --- | --- |
| 5 | Thalanany MM et al | Yes | Yes | Unclear | Unclear | Yes | Yes | Included |

Note: ① Is the specific research question clearly stated? ② Was a randomization method used? ③ Was blinding implemented for the subjects, intervention implementers, and outcome assessors? ④ Are the baseline characteristics comparable between the groups? ⑤ Apart from the intervention being verified, are other measures received by each group the same? ⑥ Are all eligible subjects included in the outcome analysis?

5、Thalanany MM, Mugford M, Hibbert C, Cooper NJ, Truesdale A, Robinson S, Tiruvoipati R, Elbourne DR, Peek GJ, Clemens F, Hardy P, Wilson A; CESAR Trial Group. Methods of data collection and analysis for the economic evaluation alongside a national, multi-centre trial in the UK: conventional ventilation or ECMO for Severe Adult Respiratory Failure (CESAR). BMC Health Serv Res. 2008 Apr 30;8:94. doi: 10.1186/1472-6963-8-94.

**Quality Evaluation of Cohort Study Literature (CASP) (n=23)**

| **No.** | **Included Literature** | **①** | **②** | **③** | **④** | **⑤** | **⑥** | **Evaluation Result** |
| --- | --- | --- | --- | --- | --- | --- | --- | --- |
| 6 | Serpa Neto A et al | Yes | Yes | Unclear | Unclear | Yes | Yes | Included |
| 7 | Schmidt M et al | Yes | Yes | Yes | Yes | Yes | Yes | Included |
| 8 | Fernando SM et al | Yes | Yes | Yes | Yes | Yes | Yes | Included |
| 9 | Risnes I et al | Yes | Yes | Yes | Unclear | Yes | Yes | Included |
| 10 | Oh TK et al | Yes | Yes | Yes | Yes | Yes | Yes | Included |
| 11 | Tramm R et al | Yes | Yes | Yes | Yes | Yes | No | Included |
| 12 | Shao C et al | Yes | Yes | Yes | Unclear | Yes | Yes | Included |
| 14 | Hodgson CL et al | Yes | Yes | Yes | Yes | Yes | Yes | Included |
| 16 | Grasselli G et al | Yes | Yes | Yes | Yes | Yes | Yes | Included |
| 17 | Kanji HD et al | Yes | Yes | Yes | Yes | Yes | No | Included |
| 18 | Ozgur MM et al | Yes | Yes | Yes | Unclear | Yes | Yes | Included |
| 19 | Guenther SPW et al | Yes | Yes | Yes | Unclear | Yes | No | Included |
| 20 | Spangenberg T et al | Yes | Yes | Yes | Yes | Yes | Unclear | Included |
| 22 | Oude Lansink-Hartgring A et al | Yes | Yes | Yes | Yes | Yes | No | Included |
| 26 | Ayers B et al | Yes | Yes | Yes | Unclear | Unclear | Yes | Included |
| 27 | Lansink-Hartgring AO et al | Yes | Yes | Yes | Yes | Yes | No | Included |
| 29 | Harley O et al | Yes | Yes | Yes | Yes | Yes | Yes | Included |
| 31 | Dardik G et al | Yes | Yes | Yes | Yes | Yes | Yes | Included |
| 33 | von Bahr V et al | Yes | Yes | Yes | Unclear | Yes | Yes | Included |
| 35 | Norkiene I et al | Yes | Yes | Yes | Unclear | Yes | Yes | Included |
| 36 | Rossong H et al | Yes | Yes | Yes | Yes | Yes | Yes | Included |
| 38 | Hanuna M et al | Yes | Yes | Yes | Unclear | Yes | Yes | Included |
| 39 | Provaznik Z et al | Yes | Yes | Yes | Unclear | Yes | Yes | Included |

Note: ① Is the method of sample inclusion appropriate, and can it represent the research population? ② Is the measurement of exposure factors accurate? ③ Is the outcome assessment method appropriate and reliable? ④ Are all important confounding factors considered in the study design and data analysis? ⑤ Is the follow-up time for the study subjects sufficient? ⑥ Has follow-up been conducted on all subjects?

6、Serpa Neto A, Higgins AM, Bailey MJ, Anderson S, Bernard S, Fulcher BJ, Jones A, Linke NJ, Board JV, Brodie D, Buhr H, Burrell AJC, Cooper DJ, Fan E, Fraser JF, Gattas DJ, Hopper IK, Huckson S, Litton E, McGuinness SP, Nair P, Orford N, Parke RL, Pellegrino VA, Pilcher DV, Dicker C, Reddi BAJ, Stub D, Trapani TV, Udy AA, Hodgson CL; EXCEL Study Investigators on behalf of the International ECMO Network (ECMONet). Long-Term Functional Outcomes in the First 12 Months After VA-ECMO in Adult Patients: A Prospective, Multicenter Study. Circ Heart Fail. 2025 Jun;18(6):e012476. doi: 10.1161/CIRCHEARTFAILURE.124.012476.

7、Schmidt M, Zogheib E, Rozé H, Repesse X, Lebreton G, Luyt CE, Trouillet JL, Bréchot N, Nieszkowska A, Dupont H, Ouattara A, Leprince P, Chastre J, Combes A. The PRESERVE mortality risk score and analysis of long-term outcomes after extracorporeal membrane oxygenation for severe acute respiratory distress syndrome. Intensive Care Med. 2013 Oct;39(10):1704-13. doi: 10.1007/s00134-013-3037-2.

8、Fernando SM, Scott M, Talarico R, Fan E, McIsaac DI, Sood MM, Myran DT, Herridge MS, Needham DM, Hodgson CL, Rochwerg B, Munshi L, Wilcox ME, Bienvenu OJ, MacLaren G, Fowler RA, Scales DC, Ferguson ND, Combes A, Slutsky AS, Brodie D, Tanuseputro P, Kyeremanteng K. Association of Extracorporeal Membrane Oxygenation With New Mental Health Diagnoses in Adult Survivors of Critical Illness. JAMA. 2022 Nov 8;328(18):1827-1836. doi: 10.1001/jama.2022.17714.

9、Risnes I, Wagner K, Nome T, Sundet K, Jensen J, Hynås IA, Ueland T, Pedersen T, Svennevig JL. Cerebral outcome in adult patients treated with extracorporeal membrane oxygenation. Ann Thorac Surg. 2006 Apr;81(4):1401-6. doi: 10.1016/j.athoracsur.2005.10.008.

10、Oh TK, Cho HW, Lee HT, Song IA. Chronic respiratory disease and survival outcomes after extracorporeal membrane oxygenation. Respir Res. 2021 Jul 5;22(1):195. doi: 10.1186/s12931-021-01796-8.

11、Tramm R, Ilic D, Sheldrake J, Pellegrino V, Hodgson C. Recovery, Risks, and Adverse Health Outcomes in Year 1 After Extracorporeal Membrane Oxygenation. Am J Crit Care. 2017 Jul;26(4):311-319. doi: 10.4037/ajcc2017707. PMID: 28668917.

12、Shao C, Wang L, Yang F, Wang J, Wang H, Hou X. Quality of Life and Mid-Term Survival in Patients Receiving Extracorporeal Membrane Oxygenation After Cardiac Surgery. ASAIO J. 2022 Mar 1;68(3):349-355. doi: 10.1097/MAT.0000000000001473.

14、Hodgson CL, Higgins AM, Bailey MJ, Anderson S, Bernard S, Fulcher BJ, Koe D, Linke NJ, Board JV, Brodie D, Buhr H, Burrell AJC, Cooper DJ, Fan E, Fraser JF, Gattas DJ, Hopper IK, Huckson S, Litton E, McGuinness SP, Nair P, Orford N, Parke RL, Pellegrino VA, Pilcher DV, Sheldrake J, Reddi BAJ, Stub D, Trapani TV, Udy AA, Serpa Neto A; EXCEL Study Investigators on behalf of the International ECMO Network and the Australian and New Zealand Intensive Care Society Clinical Trials Group. Incidence of death or disability at 6 months after extracorporeal membrane oxygenation in Australia: a prospective, multicentre, registry-embedded cohort study. Lancet Respir Med. 2022 Nov;10(11):1038-1048. doi: 10.1016/S2213-2600(22)00248-X

16、Grasselli G, Scaravilli V, Tubiolo D, Russo R, Crimella F, Bichi F, Morlacchi LC, Scotti E, Patrini L, Gattinoni L, Pesenti A, Chiumello D. Quality of Life and Lung Function in Survivors of Extracorporeal Membrane Oxygenation for Acute Respiratory Distress Syndrome. Anesthesiology. 2019 Apr;130(4):572-580. doi: 10.1097/ALN.0000000000002624.

17、Kanji HD, Chouldechova A, Harris-Fox S, Ronco JJ, O'dea E, Harvey C, Shuster C, Thiara S, Peek GJ. Quality of life and functional status of patients treated with venovenous extracorporeal membrane oxygenation at 6 months. J Crit Care. 2021 Dec;66:26-30. doi: 10.1016/j.jcrc.2021.07.010.

18、Ozgur MM, Altinay E, Ogus H, Acar RD, Atagun Guney P, Kirali K. Functional and Social Recovery and Outcomes After Extracorporeal Membrane Oxygenation Support in COVID-19 Patients. ASAIO J. 2025 May 1;71(5):396-402. doi: 10.1097/MAT.0000000000002337.

19、Guenther SPW, Cheaban R, Hoepner L, Weinrautner N, Kirschning T, Al-Khalil R, Bruenger F, Serrano MR, Barndt I, Wiemer M, Niedermeyer J, Rudloff M, Helms S, Schramm R, Gummert JF. Functional Status and Quality of Life 6 Months After Extracorporeal Membrane Oxygenation Therapy for COVID-19-Related Pulmonary Failure. ASAIO J. 2023 Oct 1;69(10):942-949. doi: 10.1097/MAT.0000000000001993.

20、Spangenberg T, Schewel J, Dreher A, Meincke F, Bahlmann E, van der Schalk H, Kreidel F, Frerker C, Stoeck M, Bein B, Kuck KH, Ghanem A. Health related quality of life after extracorporeal cardiopulmonary resuscitation in refractory cardiac arrest. Resuscitation. 2018 Jun;127:73-78. doi: 10.1016/j.resuscitation.2018.03.036

22、Oude Lansink-Hartgring A, Miranda DDR, Mandigers L, Delnoij T, Lorusso R, Maas JJ, Elzo Kraemer CV, Vlaar APJ, Raasveld SJ, Donker DW, Scholten E, Balzereit A, van den Brule J, Kuijpers M, Vermeulen KM, van den Bergh WM; Dutch ECLS Study group. Health-related quality of life, one-year costs and economic evaluation in extracorporeal membrane oxygenation in critically ill adults. J Crit Care. 2023 Feb;73:154215. doi: 10.1016/j.jcrc.2022.154215.

26、Ayers B, Bjelic M, Kumar N, Wood K, Barrus B, Prasad S, Gosev I. Long-term renal function after venoarterial extracorporeal membrane oxygenation. J Card Surg. 2021 Mar;36(3):815-820. doi: 10.1111/jocs.15277

27、Lansink-Hartgring AO, van der Bij W, Verschuuren EA, Erasmus ME, de Vries AJ, Vermeulen KM, van den Bergh WM. Extracorporeal Life Support as a Bridge to Lung Transplantation: A Single-Center Experience With an Emphasis on Health-Related Quality of Life. Respir Care. 2017 May;62(5):588-594. doi: 10.4187/respcare.05300. Epub 2017 Mar 21. PMID: 28325778.

29、Harley O, Reynolds C, Nair P, Buscher H. Long-Term Survival, Posttraumatic Stress, and Quality of Life post Extracorporeal Membrane Oxygenation. ASAIO J. 2020 Aug;66(8):909-914. doi: 10.1097/MAT.0000000000001095. PMID: 32740351.

31、Dardik G, Ning Y, Kurlansky P, Almodovar Cruz G, Vinogradsky A, Fried J, Topkara VK, Takeda K. Long-term outcomes of patients bridged to recovery with venoarterial extracorporeal life support. Perfusion. 2024 Nov;39(8):1629-1635. doi: 10.1177/02676591231206524.

33、von Bahr V, Kalzén H, Frenckner B, Hultman J, Frisén KG, Lidegran MK, Diaz S, Malfertheiner MV, Millar JE, Dobrosavljevic T, Eksborg S, Holzgraefe B. Long-term pulmonary function and quality of life in adults after extracorporeal membrane oxygenation for respiratory failure. Perfusion. 2019 Apr;34(1_suppl):49-57. doi: 10.1177/0267659119830244.

35、Norkiene I, Jovaisa T, Scupakova N, Janusauskas V, Rucinskas K, Serpytis P, Laurusonis K, Samalavicius R. Long-term quality of life in patients treated with extracorporeal membrane oxygenation for postcardiotomy cardiogenic shock. Perfusion. 2019 May;34(4):285-289. doi: 10.1177/0267659118815291. Epub 2018 Dec 19. PMID: 30565505.

36、Rossong H, Debreuil S, Yan W, Hiebert BM, Singal RK, Arora RC, Yamashita MH. Long-term survival and quality of life after extracorporeal membrane oxygenation. J Thorac Cardiovasc Surg. 2023 Aug;166(2):555-566.e2. doi: 10.1016/j.jtcvs.2021.10.077.

38、Hanuna M, Herz G, Stanzl AL, Li Y, Mueller CS, Kamla CE, Scherer C, Wassilowsky D, Juchem G, Orban M, Peterss S, Hagl C, Joskowiak D. Mid-Term Outcome after Extracorporeal Life Support in Postcardiotomy Cardiogenic Shock: Recovery and Quality of Life. J Clin Med. 2024 Apr 12;13(8):2254. doi: 10.3390/jcm13082254.

39、Provaznik Z, Philipp A, Müller T, Kostiantyn K, Lunz D, Schmid C, Floerchinger B. Outcome after veno-venous extracorporeal membrane oxygenation in elderly compared to younger patients: A 14-year retrospective observational study. Artif Organs. 2023 May;47(5):882-890. doi: 10.1111/aor.14454.

**Cross-sectional study literature quality evaluation by the Agency for Healthcare Research and Quality (AHRQ) (n=5)**

| **No.** | **Included Literature** | **①** | **②** | **③** | **④** | **⑤** | **⑥** | **⑦** | **⑧** | **⑨** | **⑩** | **⑪** | **Evaluation Result** |
| --- | --- | --- | --- | --- | --- | --- | --- | --- | --- | --- | --- | --- | --- |
| 13 | Tiedebohl JM et al | Yes | Yes | Yes | Yes | Yes | Unclear | Yes | Yes | Unclear | Yes | Yes | Included |
| 21 | Chen KH et al | Yes | Yes | Yes | Yes | Yes | No | Yes | Yes | Yes | Yes | Yes | Included |
| 25 | Hsieh FT et al | Yes | Yes | Yes | Yes | Yes | Yes | Yes | Yes | Unclear | Yes | Yes | Included |
| 28 | O'Brien SG et al | Yes | Yes | Yes | Yes | Yes | Yes | Yes | Yes | Yes | Yes | Yes | Included |
| 37 | Zeng, X et al | Yes | Yes | Yes | Yes | Yes | Yes | Yes | Yes | Unclear | Yes | Yes | Included |

Note: ① Is the source of the data (survey, literature review) clearly specified? ② Is there a list of inclusion and exclusion criteria for the exposed and non-exposed groups (cases and controls) or reference to previous publications? ③ Is the time period for identifying patients provided? ④ If not from a population source, is the study population continuous? ⑤ Does the reviewer's subjective factor obscure other aspects of the research subjects? ⑥ Is any assessment that does not guarantee quality described (such as detection/redetection of subjective outcome measures)? ⑦ Is the reason for excluding any patient from analysis explained? ⑧ Is there a description of how confounding factors are evaluated and/or controlled? ⑨ If possible, is an explanation given on how missing data is handled in the analysis? ⑩ Is a summary of the patient response rate and the completeness of data collection provided? ⑪ If there is follow-up, identify the percentage of expected patients with incomplete data or the follow-up results.

13、Tiedebohl JM, DeFabio ME, Bell T, Buchko BL, Woods AB. ECMO survivors' quality of life and needs after discharge: A descriptive, comparative cross-sectional pilot study. Intensive Crit Care Nurs. 2020 Aug;59:102829. doi: 10.1016/j.iccn.2020.102829. Epub 2020 Mar 12. PMID: 32173238.

21、Chen KH, Lee PS, Tsai FC, Weng LC, Yeh SL, Huang HC, Lin SS. Health-related outcomes of extracorporeal membrane oxygenation in adults: A cross-sectional study. Heart Lung. 2022 Mar-Apr;52:76-85. doi: 10.1016/j.hrtlng.2021.11.008.

25、Hsieh FT, Huang GS, Ko WJ, Lou MF. Health status and quality of life of survivors of extra corporeal membrane oxygenation: a cross-sectional study. J Adv Nurs. 2016 Jul;72(7):1626-37. doi: 10.1111/jan.12943.

28、O'Brien SG, Carton EG, Fealy GM. Long-Term Health-Related Quality of Life After Venovenous Extracorporeal Membrane Oxygenation. ASAIO J. 2020 May;66(5):580-585. doi: 10.1097/MAT.0000000000001042. PMID: 31425257.

37、Zeng, X., Yang, F., Luo, X. et al. Long-term health related quality of life in adult extracorporeal membrane oxygenation survivors: a single-centre, cross-sectional study. BMC Public Health 24, 3243 (2024). https://doi.org/10.1186/s12889-024-20782-5
