# Supplementary material for: Development of a nursing follow-up checklist for adult ECMO-treated discharged patients: a Delphi consensus and feasibility study
Source: Front Med (Lausanne). 2026 Mar 25;13:1779603. doi: 10.3389/fmed.2026.1779603 (PMC13057531; doi:10.3389/fmed.2026.1779603)
Supplement: Supplementary file 3 [file Table_3.DOCX]

**Table S1: Concentration of Expert Opinion on Primary and Secondary Indicators (Round 1)**

| **Primary Indicator** | **Concentration of Expert Opinion (on Primary Indicator)** | | | **Secondary Indicator** | **Concentration of Expert Opinion (on Secondary Indicator)** | | |
| --- | --- | --- | --- | --- | --- | --- | --- |
|  | **Importance score** $\bar{\mathbf{x}}$**+s（Mj）** | **CV** | **Kj（%）** |  | **Importance score** $\bar{\mathbf{x}}$**+s（Mj）** | **CV** | **Kj（%）** |
| Physiological Status | 4.92±0.277 | 0.056 | 92.31 | 1. General condition | 4.46±0.660 | 0.148 | 53.85 |
|  |  |  |  | 2. Diet | 4.38±0.650 | 0.148 | 46.15 |
|  |  |  |  | 3. Muscles and joints | 4.46±0.660 | 0.148 | 53.85 |
|  |  |  |  | 4. ECMO cannulation site | 4.62±0.650 | 0.141 | 69.23 |
|  |  |  |  | 5. Limb with ECMO cannula | 4.62±0.870 | 0.188 | 76.92 |
|  |  |  |  | 6. Sleep | 4.46±0.770 | 0.174 | 61.54 |
|  |  |  |  | 7. Respiratory system | 4.85±0.555 | 0.114 | 92.31 |
|  |  |  |  | 8. Circulatory system | 4.69±0.751 | 0.160 | 84.62 |
|  |  |  |  | 9. Nervous system | 4.85±0.376 | 0.077 | 84.62 |
|  |  |  |  | 10. Digestive system | 4.69±0.480 | 0.102 | 69.23 |
|  |  |  |  | 11. Urinary system | 4.54±0.660 | 0.145 | 61.54 |
| Psychological State | 4.69±0.480 | 0.102 | 69.23 | 1. Existing problems | 4.46±0.660 | 0.148 | 53.85 |
|  |  |  |  | 2. Analyze causes | 4.46±0.519 | 0.116 | 46.15 |
|  |  |  |  | 3. Countermeasures | 4.46±0.660 | 0.148 | 53.85 |
| Life Status | 4.54±0.660 | 0.145 | 61.54 | 1. Daily life | 4.46±0.776 | 0.174 | 61.54 |
|  |  |  |  | 2. Work status | 4.23±0.723 | 0.171 | 38.46 |
| Life Status | 4.69±0.480 | 0.102 | 69.23 | 1. Family finances | 4.54±0.660 | 0.145 | 61.54 |
|  |  |  |  | 2. Social interactions | 4.15±0.899 | 0.216 | 46.15 |

**Table S2: Concentration of Expert Opinion on Tertiary Indicators (Round 1)**

| **Primary Indicator** | **Secondary Indicator** | **Tertiary Indicators** | **Concentration of Expert Opinion** | | |
| --- | --- | --- | --- | --- | --- |
|  |  |  | Importance score $\bar{x}$+s（Mj） | CV | Kj（%） |
| Physiological Status | 1. General condition | 1. Body temperature, heart rate, blood pressure, oxygen saturation (SpO₂), respiration | 4.69±0.63 | 0.134 | 76.92 |
|  |  | 2. Blood glucose | 4.15±0.801 | 0.193 | 38.46 |
|  | 2. Diet | 1. Dietary regularity / Regular eating habits | 4.23±0.725 | 0.171 | 38.46 |
|  |  | 2. Diet composition / Meal balance / Dietary balance | 4.00±0.913 | 0.228 | 38.46 |
|  |  | 3. Nutritional guidance (when intake is insufficient) | 4.54±0.660 | 0.145 | 61.54 |
|  | 3. Muscles and joints | 1. Muscle strength (graded assessment) | 4.69±0.48 | 0.102 | 69.23 |
|  |  | 2. Joints (spasm, pain, fluid accumulation) | 4.62±0.506 | 0.110 | 61.54 |
|  |  | 3. 6-minute walk distance | 4.46±0.776 | 0.174 | 61.54 |
|  | 4. ECMO cannulation site | 1. Signs of unhealed wounds, such as redness, swelling, heat, pain | 4.92±0.277 | 0.056 | 92.31 |
|  |  | 2. Infection | 4.92±0.277 | 0.056 | 92.31 |
|  | 5. Limb with ECMO cannula | 1. Discomfort (toes, foot, calf, thigh, groin) | 4.85±0.376 | 0.077 | 84.62 |
|  |  | 2. Pain | 5.00±0 | 0.000 | 100 |
|  |  | 3. Numbness (hypoesthesia, tingling sensation, or formication) | 4.77±0.439 | 0.092 | 76.92 |
|  |  | 4. Paralysis / Weakness (reduced muscle strength) | 4.69±0.480 | 0.102 | 69.23 |
|  |  | 5. Foot drop | 4.92±0.277 | 0.056 | 92.31 |
|  |  | 6. Limited mobility | 4.92±0.377 | 0.056 | 92.31 |
|  |  | 7. Loss of mobility or paralysis | 5.00±0 | 0.000 | 100 |
|  | 6. Sleep | 1. Sleep quality | 4.54±0.660 | 0.145 | 61.54 |
|  |  | 2. Sleep guidance (when sleep quality is poor) | 4.31±0.751 | 0.174 | 46.15 |
|  |  | 3. Medication assistance / Pharmacological intervention | 4.23±0.832 | 0.197 | 46.15 |
|  | 7. Respiratory system | 1. Cough, sputum production | 4.54±0.660 | 0.145 | 61.54 |
|  |  | 2. Dyspnea (chest tightness, breathlessness) | 4.85±0.376 | 0.077 | 84.62 |
|  |  | 3. Lung infection, respiratory failure | 4.85±0.376 | 0.077 | 84.62 |
|  |  | 4. Oxygen therapy, invasive/non-invasive ventilation support | 4.85±0.555 | 0.114 | 92.31 |
|  | 8. Circulatory system | 1. Arrhythmia (palpitations, tachycardia/bradycardia, taking antiarrhythmic drugs) | 4.85±0.555 | 0.114 | 92.31 |
|  |  | 2. Cardiac insufficiency / Heart failure (edema, orthopnea, exertional dyspnea, use of cardiac glycosides or other inotropic agents) | 4.69±1.109 | 0.236 | 92.31 |
|  |  | 3. Vasoactive medications | 4.85±0.555 | 0.114 | 92.31 |
|  |  | 4. Mechanical circulatory support | 5.00±0 | 0.000 | 100 |
|  | 9. Nervous system | 1. Consciousness status | 4.92±0.277 | 0.056 | 92.31 |
|  |  | 2. Cognitive status | 4.69±0.480 | 0.102 | 69.23 |
|  |  | 3. Dizziness, headache | 4.62±0.506 | 0.110 | 61.54 |
|  |  | 4. Seizures | 4.77±0.439 | 0.092 | 76.92 |
|  |  | 5. Intracranial hemorrhage, cerebral infarction (with symptoms such as dysarthria, ataxia, hemiplegia) | 4.69±0.630 | 0.134 | 76.92 |
|  | 10. Digestive system | 1. Nausea, vomiting | 4.31±0.947 | 0.220 | 53.85 |
|  |  | 2. Bleeding (hematemesis, hematochezia, melena) | 4.54±0.776 | 0.171 | 69.23 |
|  |  | 3. Skin condition (jaundice, cheilitis, spider angioma, palmar erythema) | 4.62±0.650 | 0.141 | 69.23 |
|  |  | 4. Bowel movements (diarrhea, constipation) | 4.23±0.832 | 0.197 | 38.46 |
|  |  | 5. Pain (abdominal pain, abdominal distension, abdominal cramping) | 4.54±0.660 | 0.145 | 53.85 |
|  |  | 6. Pleural effusion, ascites | 4.46±1.127 | 0.253 | 69.23 |
|  | 11. Urinary system | 1. Urination (frequent urination, urgency, dysuria) | 4.08±1.115 | 0.274 | 38.46 |
|  |  | 2. Hematuria, proteinuria | 4.38±1.261 | 0.288 | 76.92 |
|  |  | 3. Abnormal urine output | 4.31±1.251 | 0.290 | 69.23 |
|  |  | 4. Blood purification therapy | 4.62±0.768 | 0.166 | 76.92 |
| Psychological State | 1. Existing problems | 1. Anxiety | 4.38±1.261 | 0.175 | 53.85 |
|  |  | 2. Depression | 4.54±0.519 | 0.114 | 53.85 |
|  |  | 3. Post-traumatic stress disorder (PTSD) | 4.46±0.660 | 0.148 | 53.85 |
|  |  | 4. Other psychological issues | 4.38±0.650 | 0.148 | 46.15 |
|  | 2. Analyze causes | 1. Medical reasons / Underlying illness | 4.62±0.506 | 0.110 | 61.54 |
|  |  | 2. Financial reasons / Economic factors | 4.31±0.630 | 0.146 | 38.46 |
|  |  | 3. Family and social pressures | 4.15±0.801 | 0.193 | 38.46 |
|  |  | 4. Work-related reasons | 4.23±0.725 | 0.171 | 38.46 |
|  | 3. Countermeasures | 1. Provide psychological guidance based on different causes | 4.23±1.166 | 0.276 | 53.85 |
|  |  | 2. Pharmacotherapy / Medication treatment | 4.38±0.650 | 0.148 | 46.15 |
| Life Status | 1. Daily life | 1. Self-care ability | 4.38±0.70 | 0.198 | 61.54 |
|  |  | 2. Quality of life | 4.62±0.650 | 0.141 | 69.23 |
|  |  | 3. Nutritional status | 4.08±1.188 | 0.291 | 46.15 |
|  | 2. Work status | 1. Unable to return to work | 4.31±0.855 | 0.198 | 53.85 |
|  |  | 2. Decreased work efficiency | 4.15±0.689 | 0.166 | 30.77 |
|  |  | 3. Restricted job type / Limited work capacity | 4.23±0.927 | 0.219 | 46.15 |
|  |  | 4. Returned to work, no impact | 4.46±0.660 | 0.148 | 53.85 |
| Social and Family Support | 1. Family finances | 1. Financial burden on the family due to ECMO | 4.46±0.660 | 0.148 | 53.85 |
|  |  | 2. Change in the family's financial situation compared to before | 4.38±0.768 | 0.175 | 53.85 |
|  | 2. Social interactions | 1. Emotional distance from family and friends | 4.31±0.855 | 0.198 | 53.85 |
|  |  | 2. Indifference toward colleagues (or classmates) and friends | 4.31±1.182 | 0.274 | 61.54 |
|  |  | 3. Decline in social status due to illness | 4.23±1.166 | 0.276 | 53.85 |

**Table S3: Concentration of Expert Opinion on Primary and Secondary Indicators (Round 2)**

| **Primary Indicator** | **Concentration of Expert Opinion (on Primary Indicator)** | | | **Secondary Indicator** | **Concentration of Expert Opinion (on Secondary Indicator)** | | |
| --- | --- | --- | --- | --- | --- | --- | --- |
|  | **Importance score** $\bar{\mathbf{x}}$**+s（Mj）** | **CV** | **Kj（%）** |  | **Importance score** $\bar{\mathbf{x}}$**+s（Mj）** | **CV** | **Kj（%）** |
| Physiological Status | 5±0 | 0 | 100 | 1. General condition | 4.69±0.480 | 0.102 | 69.23 |
|  |  |  |  | 2. Motor function and musculoskeletal system | 4.62±0.506 | 0.110 | 61.54 |
|  |  |  |  | 3. ECMO cannulation site and vascular complications | 4.92±0.277 | 0.056 | 92.31 |
|  |  |  |  | 4. Sleep status | 4.62±0.506 | 0.110 | 61.54 |
|  |  |  |  | 5. Respiratory system | 5.00±0 | 0.000 | 100 |
|  |  |  |  | 6. Circulatory system | 5.00±0 | 0.000 | 100 |
|  |  |  |  | 7. Nervous system | 5.00±0 | 0.000 | 100 |
|  |  |  |  | 8. Digestive system | 4.85±0.376 | 0.077 | 84.62 |
|  |  |  |  | 9. Urinary system | 4.85±0.376 | 0.077 | 84.62 |
|  |  |  |  | 10. Skin condition | 4.46±0.660 | 0.148 | 53.85 |
| Psychological State | 4.54±0.519 | 0.114 | 53.85 | 1. Existing problems | 4.69±0.630 | 0.134 | 84.62 |
|  |  |  |  | 2. Coping strategies | 4.62±0.650 | 0.141 | 69.23 |
| Life Status | 4.46±0.660 | 0.148 | 53.85 | 1. Ability for daily living activities | 4.69±0.630 | 0.134 | 84.62 |
|  |  |  |  | 2. Work status | 4.23±0.832 | 0.197 | 46.15 |
|  |  |  |  | 3. Medical compliance | 4.77±0.439 | 0.092 | 76.92 |
| Social and Family Support | 4.69±0.0.480 | 0.102 | 69.23 | 1. Family support system | 4.77±0.439 | 0.092 | 76.92 |
|  |  |  |  | 2. Social interaction and connections | 4.46±0.660 | 0.148 | 53.85 |
|  |  |  |  | 3. Support plan and action strategy | 4.77±0.439 | 0.092 | 76.92 |

**Table S4: Concentration of Expert Opinion on Tertiary Indicators (Round 2)**

| **Primary Indicator** | **Secondary Indicator** | **Tertiary Indicators** | **Concentration of Expert Opinion** | | |
| --- | --- | --- | --- | --- | --- |
|  |  |  | **Importance score** $\bar{\mathbf{x}}$**+s（Mj）** | **CV** | **Kj（%）** |
| Physiological Status | 1. General condition | 1. Vital signs (body temperature, heart rate, blood pressure, oxygen saturation, respiration) | 5.00±0 | 0.000 | 100 |
|  |  | 2. Blood glucose | 4.54±0.660 | 0.145 | 61.54 |
|  |  | 3. Weight changes | 4.23±0.725 | 0.171 | 38.46 |
|  | 2. Motor function and musculoskeletal system | 1. 6-minute walk test (6MWT) | 4.69±0.480 | 0.102 | 69.23 |
|  |  | 2. Cardiopulmonary exercise testing (CPET) or 1-minute sit-to-stand test | 4.77±0.439 | 0.092 | 76.92 |
|  |  | 3. Muscle weakness (graded muscle strength assessment) | 4.85±0.376 | 0.077 | 84.62 |
|  |  | 4. Muscle atrophy (changes in muscle volume of limbs and trunk) | 4.85±0.376 | 0.077 | 84.62 |
|  |  | 5. Balance and coordination disorders (unsteady standing, increased risk of falls) | 4.62±0.506 | 0.110 | 61.54 |
|  |  | 6. Decreased mobility and walking ability (difficulty rising from sitting, abnormal gait) | 4.62±0.506 | 0.110 | 61.54 |
|  |  | 7. Diminished or absent tendon reflexes | 4.62±0.650 | 0.141 | 69.23 |
|  |  | 8. Nerve injury (limb pain, numbness, tingling sensation, or formication) | 4.69±0.480 | 0.102 | 69.23 |
|  |  | 9. Joint spasm, pain, fluid accumulation | 4.54±0.660 | 0.145 | 61.54 |
|  |  | 10. Restricted joint movement and stiffness (reduced range of motion, fine motor skill impairment, difficulty climbing stairs, inability to stand, foot drop) | 4.54±0.519 | 0.114 | 53.85 |
|  |  | 11. Loss of mobility or paralysis (bedridden, wheelchair-dependent) | 4.77±0.439 | 0.092 | 76.92 |
|  | 3. ECMO cannulation site and vascular complications | 1. Bleeding and local hematoma formation | 4.92±0.277 | 0.056 | 92.31 |
|  |  | 2. Signs of infection (redness, swelling, heat, pain, purulent discharge) | 5.00±0 | 0.000 | 100 |
|  |  | 3. Poor wound healing | 4.92±0.277 | 0.056 | 92.31 |
|  |  | 4. Vascular injury (dissection, pseudoaneurysm, arteriovenous fistula) | 4.92±0.277 | 0.056 | 92.31 |
|  |  | 5. Thrombosis (arterial, venous) | 5.00±0 | 0.000 | 100 |
|  |  | 6. Limb swelling (measured by thigh/calf or arm/forearm circumference) | 5.00±0 | 0.000 | 100 |
|  |  | 7. Signs of distal limb ischemia (pain, sensory disturbance, pallor, pulselessness, motor impairment, temperature changes) | 5.00±0 | 0.000 | 100 |
|  |  | 8. Compartment syndrome | 5.00±0 | 0.000 | 100 |
|  |  | 9. Limb gangrene | 5.00±0 | 0.000 | 100 |
|  |  | 10. Amputation | 4.85±0.376 | 0.077 | 84.62 |
|  | 4. Sleep status | 1. Sleep quality (assessment scale) | 4.69±0.480 | 0.102 | 69.23 |
|  |  | 2. Sleep guidance (when sleep quality is poor) | 4.54±0.660 | 0.145 | 61.54 |
|  |  | 3. Medication assistance (type, dosage) | 4.38±0.768 | 0.175 | 53.85 |
|  | 5. Respiratory system | 1. Chronic cough, sputum production, wheezing, and similar manifestations | 4.85±0.376 | 0.077 | 84.62 |
|  |  | 2. Chest tightness, chest pain, or chest discomfort | 4.85±0.376 | 0.077 | 84.62 |
|  |  | 3. Signs of dyspnea (shortness of breath) | 4.92±0.277 | 0.056 | 92.31 |
|  |  | 4. Need for oxygen therapy, invasive/non-invasive respiratory support | 4.92±0.277 | 0.056 | 92.31 |
|  | 6. Circulatory system | 1. Chest discomfort (palpitations, feeling of unease) | 4.92±0.277 | 0.056 | 92.31 |
|  |  | 2. Arrhythmias and conduction disorders (tachycardia or bradycardia) | 4.92±0.277 | 0.056 | 92.31 |
|  |  | 3. Signs of cardiac insufficiency (limb edema, fatigue, orthopnea, exertional dyspnea) | 5.00±0 | 0.000 | 100 |
|  |  | 4. Cardiovascular medication types and dosages (antiarrhythmic, cardiotonic, vasopressor, antihypertensive, anticoagulant, etc.) | 4.92±0.277 | 0.056 | 92.31 |
|  |  | 5. Mechanical circulatory support | 5.00±0 | 0.000 | 92.31 |
|  | 7. Nervous system | 1. Manifestations such as dizziness, headache, and seizures | 4.85±0.376 | 0.077 | 84.62 |
|  |  | 2. Level of consciousness and arousal | 4.92±0.277 | 0.056 | 76.92 |
|  |  | 3. Cognitive function | 4.85±0.376 | 0.077 | 84.62 |
|  |  | 4. Neurological deficits (motor, sensory, speech, visual, swallowing) | 4.77±0.439 | 0.092 | 76.92 |
|  |  | 5. Intracranial hemorrhage or infarction (with symptoms such as ataxia, hemiplegia, etc.) | 4.85±0.376 | 0.077 | 69.23 |
|  | 8. Digestive system | 1. Nutritional risk assessment | 4.69±0.630 | 0.134 | 84.62 |
|  |  | 2. Signs of digestive intolerance (early satiety, nausea, vomiting, anorexia, diarrhea, etc.) | 4.62±0.650 | 0.141 | 76.92 |
|  |  | 3. Gastric motility disorders (delayed gastric emptying, gastric retention) | 4.77±0.599 | 0.126 | 100 |
|  |  | 4. Intestinal motility disorders (abdominal distension, abdominal pain, constipation, intestinal obstruction, etc.) | 4.69±0.630 | 0.134 | 61.54 |
|  |  | 5. Gastrointestinal bleeding (amount, characteristics, and color of vomitus and stool) | 5.00±0 | 0.000 | 84.62 |
|  | 9. Urinary system | 1. Manifestations such as frequent urination, urgent urination, and painful urination | 4.62±0.506 | 0.110 | 76.92 |
|  |  | 2. Urine volume and characteristics (hematuria, proteinuria) | 4.85±0.376 | 0.077 | 76.92 |
|  |  | 3. Type and dosage of diuretics used | 4.77±0.439 | 0.092 | 69.23 |
|  |  | 4. Receiving blood purification therapy | 4.77±0.439 | 0.092 | 92.31 |
|  | 10. Skin condition | 1. Skin manifestations such as yellow discoloration, petechiae, ecchymoses | 4.62±0.650 | 0.141 | 61.54 |
|  |  | 2. Mottling, cyanosis, ischemic necrosis of skin in distal tissues | 4.85±0.555 | 0.114 | 61.54 |
|  |  | 3. Skin integrity (pressure injuries, scarring at catheter insertion sites) | 4.54±0.660 | 0.145 | 61.54 |
|  |  | 4. Incontinence-associated dermatitis (extent, grading) | 4.31±0.630 | 0.146 | 69.23 |
| Psychological State | 1. Existing problems | 1. Anxiety | 4.62±0.650 | 0.141 | 69.23 |
|  |  | 2. Depression | 4.69±0.480 | 0.102 | 69.23 |
|  |  | 3. Post-traumatic stress disorder (PTSD) | 4.77±0.439 | 0.092 | 76.92 |
|  |  | 4. Other psychological issues | 4.54±0.660 | 0.145 | 61.54 |
|  | 2. Coping strategies | 1. Receive psychological counseling based on different causes (medical, financial, family/social stress, work-related) | 4.69±0.630 | 0.134 | 76.92 |
|  |  | 2. Access outpatient psychological counseling services | 4.54±0.660 | 0.145 | 61.54 |
|  |  | 3. Medication type and dosage | 4.46±0.776 | 0.174 | 61.54 |
| Life Status | 1. Ability for daily living activities | 1. Self-care ability | 4.77±0.599 | 0.126 | 84.62 |
|  |  | 2. Quality of life | 4.69±0.630 | 0.134 | 76.92 |
|  |  | 3. Rehabilitation training | 4.77±0.559 | 0.126 | 84.62 |
|  | 2. Work status | 1. Unable to return to work | 4.46±0.660 | 0.148 | 53.85 |
|  |  | 2. Returned to work with decreased efficiency | 4.23±0.832 | 0.197 | 46.15 |
|  |  | 3. Returned to work but restricted in job type/tasks | 4.23±0.832 | 0.197 | 46.15 |
|  |  | 4. Returned to work without any impact | 4.31±0.941 | 0.220 | 53.85 |
|  | 3. Medical compliance | 1. Lifestyle changes (dietary habits, smoking cessation, alcohol abstinence, etc.) | 4.54±0.660 | 0.145 | 61.54 |
|  |  | 2. Medication adherence | 4.69±0.480 | 0.102 | 69.23 |
|  |  | 3. Follow-up compliance | 4.69±0.480 | 0.102 | 69.23 |
| Social and Family Support | 1. Family support system | 1. Need for fixed or rotating family caregivers | 4.54±0.776 | 0.171 | 69.23 |
|  |  | 2. Change in family income compared to before | 4.54±0.660 | 0.145 | 61.54 |
|  |  | 3. Impact of illness on family finances | 4.54±0.660 | 0.145 | 61.54 |
|  |  | 4. Impact on caregivers (physical, psychological, social) | 4.46±0.776 | 0.174 | 61.54 |
|  | 2. Social interaction and connections | 1. Emotional distance from family and friends | 4.46±0.776 | 0.174 | 61.54 |
|  |  | 2. Decreased frequency of interaction or indifferent attitude toward colleagues (or classmates) and friends | 4.46±0.660 | 0.148 | 53.85 |
|  |  | 3. Social withdrawal and feelings of stigma related to illness | 4.54±0.660 | 0.145 | 61.54 |
|  |  | 4. Sense of social belonging and personal value | 4.62±0.650 | 0.141 | 69.23 |
|  |  | 5. Decline in social status due to illness | 4.54±0.660 | 0.145 | 61.54 |
|  | 3. Support plan and action strategy | 1. Public welfare rehabilitation support (physical and psychological) | 4.31±0.855 | 0.198 | 53.85 |
|  |  | 2. Employment guidance and support | 4.31±0.751 | 0.174 | 46.15 |
|  |  | 3. Caregiver support (caregiving skills, caregivers' own physical and mental health) | 4.46±0.660 | 0.148 | 61.54 |
