# Supplementary material for: Development of a nursing follow-up checklist for adult ECMO-treated discharged patients: a Delphi consensus and feasibility study
Source: Front Med (Lausanne). 2026 Mar 25;13:1779603. doi: 10.3389/fmed.2026.1779603 (PMC13057531; doi:10.3389/fmed.2026.1779603)
Supplement: Supplementary file 5 [file Table_5.DOCX]

**Extraction of literature content and corresponding items in the list**

| **Evidence Source Information** | **Extracted Core Concepts** | **Extract the content** | **Corresponding Checklist Items (Level 1 - Level 2 - Level 3)** |
| --- | --- | --- | --- |
| Literature 1: Cho SM et al. (2024)  Study Type: Guideline  Quality Grade: Grade A | Neurological function monitoring, structured follow-up plan, multidisciplinary specialist follow-up, cognitive function assessment | **Long-term Outcomes and Quality of Life****  **Pre-Discharge Care**  ① It is recommended to perform a clinical examination before discharge and use the modified Rankin Scale (mRS). For patients with neurological or cognitive impairments, neuroimaging (preferably routine brain MRI after decannulation) is reasonable.  ② A structured outpatient care plan should be developed, carefully considering post-discharge follow-up timing (ideally at 3, 6, and 12 months), location (preferably an ECMO clinic or neurologist), and ECMO-related comorbidities/complications (vascular, myopathy, chronic infection, cardiopulmonary recovery).  ③ Comprehensive education and psychosocial support should be provided to patients, family members, and caregivers.  ④ Nutritional assessment and planning are recommended to optimize recovery.  **Post-Discharge Care**  ① Serial neurological assessments and quality-of-life evaluations are advised.  ② For patients with neurological complications, specialized neurological evaluation, neuroimaging (preferably MRI), and tailored diagnostic tests are recommended.  ③ Disease-specific specialist follow-up (e.g., pulmonologists, cardiologists, neurologists, nephrologists, gastroenterologists, hematologists) for underlying conditions and comorbidities should be arranged as needed.  ④ Coordination with primary care providers is essential.  ⑤ A centralized, secure data repository for sharing patient information with outpatient healthcare providers is recommended.  **Neurological Outcomes and Quality of Life**  All patients should undergo mRS assessment at discharge and during each follow-up. Additional detailed evaluations (e.g., Glasgow Outcome Scale-Extended, Montreal Cognitive Assessment) may be conducted based on local practices and individual patient needs. | 1. Physiological Status - Nervous System - Cognitive Function (ICU Post-Syndrome Screening)  2. Physiological Status - Nervous System - Neurological Deficits (Motor, Sensory, Speech, Visual, Swallowing)  3. Social and Family Support - Support Plan and Action Steps |
| Literature 2: Chen Jinmeng et al. (2024)  Study Type: Quasi-experimental Study  Quality Grade: Moderate | Quality of life assessment, anxiety and depression monitoring, cognitive function (MMSE), rehabilitation intervention | Quality of life (SF-36 score) and Mini-Mental State Examination (MMSE) score are protective factors against the development of complications in critically ill survivors treated with ECMO.  Hamilton Depression Rating Scale (HAMD) score and Hamilton Anxiety Rating Scale (HAMA) score are both risk factors for complications in these patients. HAMD and HAMA serve as effective indicators for evaluating patients' mental health status, while MMSE can objectively demonstrate cognitive impairment.  **Interventions during ECMO treatment:**  During ECMO therapy, early risk assessment should be conducted upon hospitalization using tools such as the Survival Prediction Score for Respiratory Diseases and the Cardiogenic Shock Outcome Prediction Score post-V-A ECMO for acute myocardial infarction. Close monitoring and interventions should be implemented for identified risk areas.  **Post-ECMO interventions**:  After ECMO treatment, rehabilitation nursing interventions should be initiated promptly, with effective management to prevent catheter displacement or accidental extubation. Early rehabilitation interventions positively contribute to cardiopulmonary function and psychological well-being.  **Post-discharge interventions:**  Following discharge, regular follow-up services provide professional guidance to alleviate patients' anxiety about uncertain prognostic outcomes. Communication with family members enables accurate assessment of recovery progress, thereby preventing non-compliance with follow-ups, reluctance to disclose information, and delays in rehabilitation. | 1. Physiological Status - Nervous System - Dizziness, Headache  2. Psychological Status - Existing Problems - Anxiety  3. Psychological Status - Existing Problems - Depression  4. Living Status - Activities of Daily Living - Rehabilitation Training |
| Literature 3: Wang Fengzhen et al. (2024)  Study Type: Qualitative Study  Quality Grade: High | Changes in physical function, changes in psychological state, active adaptation to life, strong demand for rehabilitation | A qualitative study was conducted using a phenomenological approach, selecting 13 patients who were discharged after receiving ECMO treatment at a cardiac center in Ganzhou City from January 2017 to June 2018 for semi-structured interviews.  **Participants:** ECMO patients 6-12 months post-discharge.  **Four major themes were identified**: changes in physical function, changes in psychological state, active adaptation to life, and strong demand for rehabilitation.  **① Life of ECMO-treated patients remains affected by complications after discharge.**  Healthcare providers should pay attention to the physical and psychological complications of ECMO-treated patients and strengthen their continued care after discharge. It is recommended to develop intervention measures based on different catheterization modes and primary diseases to help patients recover after discharge.  **② ECMO-treated patients experience significant stress.**  Before providing psychological interventions, healthcare providers should analyze the sources of stress for the patients to improve the effectiveness of these interventions. Additionally, it is advisable to call on the government to offer more support in the medical insurance reimbursement ratio for ECMO consumables, thereby alleviating the financial burden on ECMO patients.  **③ ECMO-treated patients adopt positive attitudes toward life, and multi-level support contributes to patient recovery.**  In their work, healthcare providers should fully utilize the patients' proactive mindset towards rehabilitation, provide more comprehensive professional knowledge on rehabilitation, and recommend appropriate self-exercise and dietary adjustment methods based on different symptoms. Besides encouraging social support from family, friends, and fellow patients, healthcare providers should also offer spiritual care tailored to the cultural and religious needs of ECMO patients.  **④ ECMO-treated patients have a strong demand for post-discharge rehabilitation services and crave professional rehabilitation knowledge.**  Medical professionals should enhance their focus on the need for rehabilitation knowledge among ECMO-treated patients post-discharge. Under conditions of unequal urban-rural medical resources, efforts should be made to overcome difficulties and provide convenient, efficient disease rehabilitation guidance to patients.  **Conclusion:**  Healthcare providers should promptly develop meticulous and comprehensive continuing care plans for ECMO-treated patients, offering multi-level support to help them address physical, psychological, and socio-life issues, thereby improving their quality of life.。 | 1. Physiological Status - Motor Function and Musculoskeletal System - Functional Activity Disorders (Difficulty Climbing Stairs, Inability to Stand, etc.)  2. Psychological Status - Existing Problems - Anxiety  3. Living Status - Activities of Daily Living - Rehabilitation Training  4. Social and Family Support - Family Support System - Needs of Fixed/Rotating Family Caregivers |
| Literature 4: Chen Yuanyuan et al. (2024)  Study Type: Meta-synthesis of Qualitative Studies  Quality Grade: High | Dynamic assessment of physical and psychological conditions, social support network, continuous rehabilitation nursing, remote rehabilitation guidance | **① Dynamically assess the physical and psychological conditions of patients undergoing ECMO treatment, and implement individualized treatments and support.**  **- Before ECMO treatment**: Medical staff should inform patients and their families about the expected outcomes and potential risks of ECMO therapy, providing information on treatment costs, insurance coverage, and financial assistance.  **- During ECMO support**: Nurses should closely monitor the operation status of the ECMO machine, actively prevent and manage various complications.  **- After weaning from ECMO:** In addition to progressively implementing rehabilitation training, nurses also need to pay attention to the patient's psychological state. Music therapy can be used to alleviate the patient's psychological stress, flexible arrangements for family visits and video communication should be made to provide emotional support.  **② Improve social support networks and assist patients in actively adapting to life changes.**  Medical staff can help patients establish, improve, and fully utilize social support networks by linking community resources, providing guidance on economic aid, organizing and guiding patients to join ECMO mutual aid groups, and using social media and online platforms. This will help them adapt to life changes after ECMO treatment.  **③ Conduct continuous rehabilitation and nursing care to meet the needs of patients recovering their health.**  For patients receiving ECMO treatment before discharge, educate them and their families on disease management and rehabilitation training knowledge in advance. If conditions permit, help patients connect with community rehabilitation centers. For patients in remote areas, remote medical technology can be used to provide online rehabilitation monitoring and guidance to ensure the continuity of rehabilitation.  Medical institutions can develop short-term and long-term follow-up plans based on the needs of patients, especially those within six months of discharge, using the 4C continuous nursing model to provide targeted continuous nursing care and guidance, thereby improving the survival rate and quality of life of patients. | 1. Physiological Status - General Condition - Vital Signs  2. Psychological Status - Coping Strategies/Interventions - Etiology-Based Psychological Counseling  3. Social and Family Support - Family Support System - Impact of Illness on Caregivers  4. Living Status - Activities of Daily Living - Rehabilitation Training |
| Literature 5: Thalanany MM et al. (2008)  Study Type: Multicenter RCT  Quality Grade: High | 6-month quality of life, functional status assessment, cost-utility analysis | The CESAR trial's economic evaluation provides a comprehensive framework, focusing on the precise measurement and valuation of resource use (ICU costs), clinical outcomes (survival, disability, quality of life), and economic metrics (costs, cost-effectiveness ratio, cost-utility ratio).  **① Study Objectives:**  - To compare the cost-effectiveness and cost-utility differences between patients undergoing ECMO and those receiving mechanical ventilation at the 6-month follow-up.  - Cost-effectiveness: Survival rate without severe disability (inability to care for oneself and bedridden) at 6 months.  - Cost-utility: Quality-adjusted life years (QALYs) measured using the Euroqol EQ-5D at 6 months; projected and modeled lifetime (based on assumptions and models) cost-utility.  **② Data Collection:**  a. Treatment phase: Data on organ support, transportation, and outcome-related information.  b. Post-discharge phase: Event recording, patient cost questionnaires at 6 months.  c. Special study on ICU costs.  d. Special study on visiting costs: Separate estimation of social costs associated with ICU visits (time loss, transportation expenses, income loss, etc.).  e. Unit costs: Ambulance, medication, transportation, wages, etc.  **③ Significance of the Study:**  a. The first adult ECMO randomized controlled trial (RCT) to incorporate a comprehensive economic assessment into its design.  b. Cost collection covered ICU, hospitalization, transportation, post-discharge services, and social costs. An organ support weighting system was developed to adjust ICU costs.  c. Provides high-quality evidence on the cost-effectiveness of ECMO for health technology assessment agencies in the UK and international decision-makers. | 1. Physiological Status - Motor Function and Musculoskeletal System - 6-Minute Walk Test  2. Living Status - Activities of Daily Living - Self-Care Ability  3. Physiological Status - Respiratory System - Dyspnea (Wheezing, Shortness of Breath) |
| Literature 6: Serpa Neto A et al. (2025)  Study Type: Prospective Cohort Study  Quality Grade: High | 12-month functional outcomes, cognitive impairment, activities of daily living, employment status | **Objective:** To investigate the incidence of death or new-onset disability within 12 months after the initiation of VA-ECMO.  **Population**: 389 patients receiving VA-ECMO across 26 hospitals in Australia and New Zealand (diagnostic groups: perioperative support, myocardial infarction, acute decompensated heart failure).  **Outcome Measures:**  **- Primary Outcome:** Death or new-onset disability at 6 and 12 months (defined as a ≥10% increase in World Health Organization Disability Assessment Schedule 2.0 [WHODAS] score from baseline) — a composite endpoint.  **- Secondary Outcomes:**  - WHODAS score (expressed as percentage),  - Incidence of new-onset disability,  - European Quality of Life Visual Analogue Scale (EQ-VAS),  - Cognitive impairment (Montreal Cognitive Assessment <18 points),  - Complete dependence (Instrumental Activities of Daily Living [IADL] ≤2),  - Complete independence (IADL = 8),  - Unemployment due to health reasons.  **Results:** The incidence of death or new-onset disability was 70.6% at 12 months and 70.8% at 6 months (adjusted odds ratio [aOR], 0.61; 95% confidence interval [CI], 0.25–1.49; P=0.27). At 12 months, more patients achieved self-care in daily living (62.1% vs 48.2%; OR, 2.84; 95% CI, 1.50–5.36; P=0.001), and fewer were unemployed due to health reasons (32.7% vs 47.4%; OR, 0.29; 95% CI, 0.13–0.65; P<0.001).  **Conclusion**: At 12 months post-ECMO initiation, 30% of patients survived without disability, with outcomes varying by reason for ECMO initiation (i.e., diagnostic group). Disability primarily developed during the first 6 months after ECMO initiation and persisted through the 6- to 12-month period. | 1. Physiological Status - Nervous System - Cognitive Function (ICU Post-Syndrome Screening)  2. Living Status - Activities of Daily Living - Self-Care Ability  3. Physiological Status - Motor Function and Musculoskeletal System - Muscle Function Status (Muscle Strength, Muscle Atrophy) |
| Literature 7: Matthieu S et al. (2013)  Study Type: Multicenter Retrospective Study  Quality Grade: Moderate | Anxiety, depression, PTSD, quality of life (SF-36), respiratory symptoms, daily function | **① Objectives:**  a. To identify factors associated with death within 6 months after discharge in ARDS patients treated with ECMO.  b. To develop a practical mortality risk prediction score (the PRESERVE score).  c. To assess health-related quality of life (HRQL), respiratory symptoms, and the prevalence of anxiety, depression, and post-traumatic stress disorder (PTSD) among long-term survivors.  **② Methods**:  a. Analysis of data from 140 ARDS patients treated with ECMO admitted to three French ICUs between 2008 and 2012. Data included baseline characteristics, parameters prior to ECMO initiation, treatment course, and outcomes (6-month mortality).  **③ Long-Term Follow-Up Assessments:**  For patients who survived at least 6 months after ICU discharge:  - Health status evaluated using the SF-36 questionnaire  - Respiratory condition assessed via the St. George’s Respiratory Questionnaire (SGRQ)  - Anxiety and depressive symptoms measured by the Hospital Anxiety and Depression Scale (HAD)  - PTSD symptoms assessed using the Impact of Event Scale (IES)  - Daily functioning evaluated through Activities of Daily Living (ADL) and Instrumental Activities of Daily Living (IADL) scales  **④ Results:**  a. The 6-month survival rate was 60%.  b. Among survivors, 80% reported persistent physical and emotional distress related to HRQL; 36% experienced ongoing dyspnea. Additionally, 34%, 25%, and 16% reported symptoms of anxiety, depression, or PTSD, respectively. At follow-up, 72% had returned to work.  c. ICU-acquired diaphragm dysfunction and respiratory muscle weakness may contribute to the persistence of these symptoms.  d. Future research should focus on understanding the pathophysiology of critical illness musculoskeletal sequelae and developing individualized, patient-centered rehabilitation programs to improve long-term outcomes.  **⑤ Conclusions:**  a. The PRESERVE score, incorporating variables such as age, immunocompromised status, SOFA score, duration of mechanical ventilation, absence of prone positioning, PEEP, plateau pressure (Pplat), and BMI, demonstrated excellent predictive performance. It can assist ICU clinicians in selecting appropriate candidates for ECMO among severe ARDS patients.  b. Despite successful life-saving ECMO therapy, long-term survivors face significant physiological (physical limitations, breathlessness) and psychological burdens (anxiety, depression, PTSD risk), which negatively impact their quality of life.  c. Further validation of the PRESERVE score in broader populations is needed.  d. Emphasis should be placed on implementing targeted rehabilitation interventions aimed at improving both physical function and mental health in ECMO survivors. | 1. Psychological Status - Existing Problems - Anxiety  2. Psychological Status - Existing Problems - Depression  3. Psychological Status - Existing Problems - Post-Traumatic Stress Disorder (PTSD)  4. Physiological Status - Respiratory System - Chronic Cough, Sputum Production  5. Living Status - Activities of Daily Living - Self-Care Ability |
| Literature 8: Fernando SM et al. (2022)  Study Type: Retrospective Cohort Study  Quality Grade: High | New-onset mental illness, anxiety, depression, PTSD, social problems | **① Objective:**  To investigate whether ECMO treatment is associated with an increased risk of new-onset mental health diagnoses or social problems after hospital discharge among ICU survivors. To compare outcomes between ECMO survivors and non-ECMO ICU survivors.  **② Methods:**  Adult patients admitted to ICUs in Ontario, Canada, from April 1, 2010, to March 31, 2020, who survived to hospital discharge. Included 642 ECMO survivors and 3,820 non-ECMO ICU survivors.  **- Primary Outcome:** New-onset mental health diagnosis or social problem (composite outcome), determined using the ICES Mental Health and Addictions Scorecard criteria. This included:  - Mood disorders  - Anxiety disorders  - Post-traumatic stress disorder (PTSD)  - Schizophrenia or other psychotic disorders  - Other mental health diagnoses (e.g., adjustment reactions, personality disorders)  - Social problems (e.g., financial, marital, family, occupational, legal issues)  **- Secondary Outcomes:** Substance abuse, self-harm, suicide death, and individual components of the primary outcome.  **③ Results:**  - **Primary Outcome (New-onset mental health diagnosis or social problem):**  - **Incidence rate**:  - ECMO survivors: 36.8%, 22.1 per 100 person-years (95% CI, 19.5–25.1)  - Non-ECMO ICU survivors: 40.9%, 14.5 per 100 person-years (95% CI, 13.8–15.2)  - Absolute incidence difference: +7.6 per 100 person-years (95% CI, 4.7–10.5)  - Hazard ratio (HR): 1.24 (95% CI, 1.01–1.52) — indicating a significantly 24% higher risk for ECMO survivors.  - **Secondary Outcomes**:  - No significant differences in substance abuse or self-harm rates.  - Suicide deaths: fewer than 5 cases total; no meaningful comparison possible.  - Risk factors for new-onset mental health diagnoses in ECMO survivors:  - Pre-existing mental health diagnosis  - Psychiatric outpatient visits within one year prior to admission  **④ Discussion**:  a. Mental health issues are relatively common among ECMO survivors, most frequently depression, anxiety, and PTSD—likely linked to the traumatic nature of invasive ICU treatments.  b. Findings help identify vulnerable patient subgroups that may benefit from additional psychosocial support.  c. Emphasizes the importance of long-term mental health monitoring and intervention for ECMO survivors.  d. Calls for further exploration of prognostic factors contributing to new-onset mental health conditions post-ECMO.  e. The question of whether ECMO directly causes mental illness remains controversial due to lack of clear mechanistic evidence.  **⑤ Conclusion:**  Patients treated with ECMO have a slightly increased risk of being diagnosed with new mental health conditions or experiencing social problems after hospital discharge. Further research is needed to clarify the underlying mechanisms behind this association. | 1. Psychological Status - Existing Problems - Anxiety  2. Psychological Status - Existing Problems - Depression  3. Psychological Status - Existing Problems - Post-Traumatic Stress Disorder (PTSD)  4. Social and Family Support - Social Interaction and Network |
| Literature 9: Risnes I et al. (2006)  Study Type: Case Series  Quality Grade: Moderate | Cerebral outcomes, cognitive deficits, neuroimaging abnormalities, neurological complications of VA-ECMO | **Background:**  ECMO (Extracorporeal Membrane Oxygenation) carries a high risk of brain injury. This study investigated the long-term brain function status in 28 adult patients at an average of 5.0 years (range: 0.5–12 years) after ECMO treatment for severe cardiopulmonary failure.  **Methods:**  A comprehensive assessment was conducted on 28 survivors, including medical history review, physical examination, neuropsychological testing, electroencephalography (EEG), and neuroradiological evaluation.  **Results:**  - All patients were able to walk independently; 43% had no clinical symptoms.  - Neuropsychological impairments were present in 41% of patients.  - Abnormalities on neuroimaging were found in 52%.  - Pathological EEG changes occurred in 41%.  - Cognitive outcomes correlated significantly with neuroimaging findings.  - The incidence of imaging abnormalities—such as cerebral infarction, microemboli, or hemorrhage—was higher in the venoarterial (VA)** group compared to the venovenous (VV) group (75% vs. 17%).  - No association was observed between ECMO type and neuropsychological dysfunction.  - EEG results showed no correlation with either cognitive performance or neuroimaging abnormalities.  **Conclusions:**  Cerebral sequelae are more common among VA-ECMO patients. There is a significant correlation between cognitive deficits and neuroradiological abnormalities.  **Follow-up Assessment Details**:  **Comprehensive Evaluation Protocol Included**:  - Medical history  - Physical examination  - Neuropsychological assessment  - Neuroradiological evaluation (brain MRI)  - Resting-state 21-channel scalp EEG  **Neuropsychological Tests Administered**:  - Wechsler Adult Intelligence Scale – Revised (WAIS-R)  - Standardized neuropsychological battery assessing:  - Motor coordination: Purdue Pegboard Test  - Psychomotor speed: Digit Symbol Substitution Test, Trail Making Test Part A & B  - Attention: Digit Span Test, Stroop Color-Word Interference Test  - Verbal learning and memory: Rey Auditory Verbal Learning Test (RAVLT)  - Visual memory: Rey-Osterrieth Complex Figure Test (RCFT)  - Verbal fluency: Controlled Oral Word Association Test (COWAT)  **Findings from Neuropsychological Testing**:  The cohort performed within the mildly impaired range across most cognitive domains. Scores on verbal fluency tests were slightly above average. Impairments were particularly noted in attention (Stroop test) and verbal memory (RAVLT).  **Neuroradiological Assessment:** Brain Magnetic Resonance Imaging (MRI)  **EEG Assessment**: Resting-state 21-lead scalp EEG  **Discussion**:  ECMO therapy itself may contribute to brain injury. Patients often experience periods of hypoxia before and during cannulation. The procedure of cannulation, along with solid and gaseous microemboli formed during perfusion, may lead to cerebral damage—risks that are amplified in VA-ECMO due to direct arterial cannulation.  **Different follow-up emphases based on ECMO mode**:  - VA-ECMO: Greater focus on potential neurological injury- VV-ECMO: More emphasis on pulmonary recovery, though neurological risks remain relevant | 1. Physiological Status - Nervous System - Intracerebral Hemorrhage, Infarction (Ataxia, Hemiplegia, etc.)  2. Physiological Status - Nervous System - Neurological Deficits (Motor, Sensory, Speech, Visual, Swallowing)  3. Physiological Status - ECMO Catheter Site and Vascular Complications - Limb Ischemia or Compartment Syndrome |
| Literature 10: Oh TK et al. (2021)  Study Type: Cohort Study  Quality Grade: Moderate | Chronic respiratory diseases, impaired lung function, long-term survival | **Background:** This study aims to describe the characteristics of patients who develop chronic respiratory diseases (CRDs) after receiving Extracorporeal Membrane Oxygenation (ECMO) therapy and to investigate the association between newly diagnosed post-ECMO CRD and the 5-year all-cause mortality among ECMO survivors.  The quality of life (QOL) for ECMO survivors has become a significant public health issue. Among the factors contributing to a decline in their QOL, impaired pulmonary function after discharge is a critical one.  **Definitions:**   - **ECMO Survivor:** A patient who survived for more than 365 days after the initiation of ECMO therapy. - **Primary Outcome:** 5-year all-cause mortality in ECMO survivors (defined as death from any cause occurring within 5 years after ECMO initiation). - **Secondary Outcome:** New-onset heart failure within 365 days post-ECMO, assessed within 365 days after ECMO initiation. - **Chronic Respiratory Diseases (CRDs):** Include Chronic Obstructive Pulmonary Disease (COPD), interstitial lung disease, asthma, lung cancer, extrinsic lung disease, obstructive sleep apnea, and tuberculosis. - **Comorbidities:** Assessed using the Charlson Comorbidity Index (CCI), which is calculated from 17 predefined conditions and ranges from a score of 0 (best) to 27 (worst).   **Pathophysiology Note:** During VA-ECMO therapy, multiple factors can lead to persistent lung injury, triggering pulmonary inflammation and fibrosis, which results in long-term pulmonary dysfunction in VA-ECMO survivors. | 1. Physiological Status - Respiratory System - Chronic Cough, Sputum Production  2. Physiological Status - Respiratory System - Chest Tightness, Chest Pain, Chest Discomfort  3. Physiological Status - Respiratory System - Oxygen Therapy, Invasive/Non-Invasive Respiratory Support |
| Literature 11: Tramm R et al. (2017)  Study Type: Prospective Longitudinal Study  Quality Grade: High | Pain and neurological injury at catheter site, foot drop, anxiety, depression, PTSD | **Objective:** To investigate the one-year post-discharge recovery (both physical and psychological) of patients who received Extracorporeal Membrane Oxygenation (ECMO) therapy.  **Follow-up Schedule:** Prospective data collection was conducted at 3, 6, and 12 months after ECMO treatment.  **Follow-up Methods:** Telephone interviews and mailed questionnaires.  **Assessment Scales:**   - The 36-Item Short Form Health Survey (SF-36) - EQ-5D-5L - Hospital Anxiety and Depression Scale (HADS) - Impact of Event Scale-Revised (IES-R) - The Telephone Interview for Cognitive Status (TICS) was used to assess cognitive function after each telephone follow-up.   **Interview Outcomes:**   - **Adverse Health Outcomes:** Pain or neurological injury at the ECMO cannulation site, mobility issues related to ECMO, and foot drop. - **Social and Economic Status:** Household income, employment status, ability to drive a car, and social and material support. - **Healthcare Service Utilization:** Outpatient visits, readmissions, number of hospitalizations, general practitioner (GP) visits, physical therapy, psychological counseling (and frequency), and cardiac rehabilitation.   **Results:** Fifty percent of patients experienced pain and neurological dysfunction related to the ECMO cannulation site or leg. Severe conditions such as foot drop were particularly prominent (associated with direct stimulation or local injury to the groin area and femoral nerve in VA-ECMO patients). The symptoms of foot drop gradually improved over time.  **Conclusions:** ① Physical impairment was more severe than psychological impairment, although both domains showed improvement over time. ② The prevalence of anxiety, depression, and PTSD was two to three times higher than that in the general community population. ③ The mobility dimension of the EQ-5D-5L scale is helpful for the early identification of lower limb neurological problems. If its validity can be confirmed by other researchers, it has the potential to become a core tool for evaluating treatment outcomes in ECMO patients. ④ Cognitive dysfunction requires further in-depth investigation, including the validation of measurement tools and the reliability of their results. ⑤ Future research should focus on exploring the impact of ECMO therapy and its associated risk factors on cognitive impairment. | 1. Physiological Status - ECMO Catheter Site and Vascular Complications - Poor Wound Healing (Bleeding, Local Hematoma)  2. Physiological Status - Motor Function and Musculoskeletal System - Functional Activity Disorders (Foot Drop)  3. Psychological Status - Existing Problems - Anxiety  4. Psychological Status - Existing Problems - Depression  5. Psychological Status - Existing Problems - Post-Traumatic Stress Disorder (PTSD) |
| Literature 12: Shao C et al. (2022)  Study Type: Retrospective Study  Quality Grade: Moderate | Quality of life (SF-36), mid-term survival, daily function | **Objective:** To evaluate the survival rates and quality of life (QOL) by comparing patients who received ECMO therapy for Post-Cardiotomy Shock (PCS) with a control group of cardiac surgery patients who did not receive ECMO.  **Methods:** The follow-up cohort consisted of 102 post-cardiac surgery patients who received ECMO and 102 post-cardiac surgery patients who did not receive ECMO.  **Quality of Life Assessment Tool:** The QOL was assessed using the SF-36 survey questionnaire, which includes eight dimensions: Physical Functioning (PF), Role-Physical (RP), Bodily Pain (BP), General Health (GH), Vitality (VT), Social Functioning (SF), Role-Emotional (RE), and Mental Health (MH). Each dimension is scored from 0 to 100.  **Follow-up Methods:** Follow-up was conducted via telephone interviews.  **Follow-up Schedule:** Follow-up assessments were performed at 1 month, 6 months, 1 year, 3 years, and 5 years after discharge.  **Results:** There were no significant differences between the two groups in terms of employment status, complications, readmission rates, or subsequent surgical procedures. The ECMO group had significantly lower scores for General Health (GH) and Vitality (VT) compared to the control group. Within the ECMO group, the highest SF-36 composite scores were observed at year 5, while the lowest scores were at year 1.   - At the 1-year follow-up: The VT score in the ECMO group was lower than in the control group. - At the 3-year follow-up: The RP and GH scores in the ECMO group showed a significant decline compared to the control group. - At the 5-year follow-up: No significant differences were observed between the two groups.   **Conclusion:** The majority of patients who survived the first month after discharge achieved a 5-year survival rate. | 1. Physiological Status - General Condition - Vital Signs  2. Living Status - Activities of Daily Living - Self-Care Ability  3. Physiological Status - Circulatory System - Cardiac Insufficiency (Limb Edema, Fatigue, etc.) |
| Literature 13: Tiedebohl JM et al. (2020)  Study Type: Cross-sectional Study  Quality Grade: Moderate | Quality of life (SF-36), gender differences, mental health needs, needs for follow-up coordinator | **Objective**: To describe gender differences in health-related quality of life (HRQoL) among patients after Extracorporeal Membrane Oxygenation (ECMO) therapy and to analyze survivors’ perceptions of follow-up care needs post-discharge.  **Methods and Design**: A descriptive, comparative, cross-sectional pilot study was conducted. The study population comprised adult patients who were discharged after receiving ECMO therapy between January 1, 2016, and March 31, 2018.  **Follow-up Method:** A 20- to 30-minute telephone interview was conducted.  Assessment Tool: The SF-36 survey, which includes eight dimensions, with each dimension scored from 0 to 100.  **Results:** **Post-Discharge Care Services:** There was a clear need for a designated “contact person” or “coordinator” to manage the continuous follow-up for patients’ complex and multifaceted needs. It was suggested that ECMO survivors establish a long-term relationship with a coordinator to facilitate subsequent follow-up and improve patient-provider communication. The need for mental health follow-up was also prominent.  **SF-36 Scores**: While scores in Role-Emotional (RE) and Mental Health (MH) were comparable to national norms, gender differences were observed in both domains.  **Conclusions**: ① ECMO survivors demonstrated impairment in all dimensions of health-related quality of life (HRQoL), with the exception of the Role-Emotional and Mental Health dimensions. ② Significant gender differences were observed in the sample: female patients scored significantly lower on all SF-36 dimensions, with particularly pronounced differences in Bodily Pain (BP), Social Functioning (SF), and Role-Emotional (RE). ③ The proportion of female patients reporting mental health problems was also higher than that of male patients. ④ There is a widespread perception among ECMO survivors of the need for long-term follow-up with a coordinator to facilitate the management of complex care pathways. ⑤ Post-discharge care, including mental health services, is crucial. Future research should aim to increase the level of evidence-based medicine by evaluating the effectiveness of long-term, coordinated, and comprehensive follow-up care for ECMO survivors through randomized controlled trials (RCTs). | 1. Psychological Status - Coping Strategies/Interventions - Etiology-Based Psychological Counseling  2. Social and Family Support - Support Plan and Action Steps  3. Living Status - Activities of Daily Living - Self-Care Ability |
| Literature 14: Hodgson CL et al. (2022)  Study Type: Prospective Multicenter Cohort Study  Quality Grade: High | 6-month disability rate, cognitive function, activities of daily living, employment status | The largest cohort study to date on the 6-month (180-day) prognosis of critically ill patients supported by ECMO, and the first study to describe changes in functional status among survivors.  **Objectives:** To assess the incidence of death or disability within 6 months (180 days) after ECMO initiation and to compare the differences in efficacy between different ECMO modes. By comparing baseline pre-ECMO data with 6-month follow-up data, the study aimed to evaluate changes in functional status and health levels.  **Follow-up Content:**  **Primary Outcome:** Death or moderate-to-severe disability at 6 months after the start of ECMO (defined using the 12-item WHO Disability Assessment Schedule 2.0). Specifically defined as any patient who experienced death or moderate-to-severe disability (i.e., a WHODAS score ≥25%).  **Secondary Outcomes:** ICU mortality, in-hospital mortality, 90-day mortality, 180-day mortality, degree of disability, new-onset disability, composite death/new-onset disability event, health status of survivors, cognitive function, activities of daily living (ADL), and work status. All indicators were observed over a 6-month period.  **Clinical Outcomes:** Use of CRRT (Continuous Renal Replacement Therapy), incidence of ECMO-related complications, duration of ECMO, duration of mechanical ventilation, total length of ICU stay, and total length of hospital stay.  **Follow-up Method:** Initially conducted via email, then switched to telephone follow-up (with up to three contact attempts made at different times within a three-week period).  **Assessment Scales:** World Health Organization Disability Assessment Schedule 2.0 (WHODAS 2.0, 12-item version), EuroQol Five-Dimension Five-Level Scale (EQ-5D-5L), EuroQol Visual Analogue Scale (EQ-VAS), Instrumental Activities of Daily Living (IADL) scale, Barthel Index (for Activities of Daily Living), and the Montreal Cognitive Assessment - Blind version (MoCA-BLIND) for cognitive impairment.  **Follow-up Time:** 6 months after ECMO initiation or at the time of death.  **Factors Influencing Health-Related Quality of Life:**  Multiple factors, including individual resilience, adaptability, post-traumatic growth following major life events, and the living environment.  **Results:** Among 391 critically ill patients who received ECMO and had follow-up data, only one-third remained free of moderate-to-severe disability at 6 months. There were significant differences in disability-free survival rates between different types of ECMO. Among the survivor group, new-onset disability was prevalent across various functional domains, including physical function, mental status, and cognitive ability.  This highlights the necessity of providing long-term medical support and care services for the ECMO population. Further research on the long-term outcomes of patients receiving different ECMO modes is needed to help formulate ICU management strategies and provide long-term healthcare. | 1. Physiological Status - Nervous System - Cognitive Function (ICU Post-Syndrome Screening)  2. Living Status - Activities of Daily Living - Self-Care Ability  3. Physiological Status - Motor Function and Musculoskeletal System - Balance and Coordination Disorders |
| Literature 15: Kurniawati ER et al. (2021)  Study Type: Systematic Review  Quality Grade: High | Quality of life (SF-36/EQ-5D), anxiety, depression, PTSD, motor function disorders | **Objective:** To provide a comprehensive overview of the Health-Related Quality of Life (HRQoL) in patients with Acute Respiratory Distress Syndrome (ARDS) after receiving Veno-venous ECMO (VV-ECMO) support.  Methods: Studies reporting HRQoL in adult ARDS patients after VV-ECMO, published between 2009 and 2020, were included.  **Results:** A total of 8 studies were included (7 observational studies and 1 randomized controlled trial), comprising 441 patients. Among them, 265 VV-ECMO survivors reported reduced HRQoL, with a follow-up period ranging from 6 months to 3 years.  The majority of survivors experienced varying degrees of anxiety, depression, and post-traumatic stress disorder (PTSD).  **Conclusion:** Survivors of ARDS supported by VV-ECMO experience a decline in HRQoL and suffer from physical and psychological impairments. This reduction in HRQoL is comparable to, and in some cases even better than, that of ARDS survivors treated with conventional therapy.  Follow-up Related Information  After patients are successfully weaned from ECMO support, they typically require comprehensive rehabilitation support, including physical therapy, occupational rehabilitation, nutritional management, and speech therapy.  **Assessment Tools:** All studies utilized the SF-36 or EQ-5D. Some studies also included other tools such as: the St. George's Respiratory Questionnaire (SGRQ), the Impact of Event Scale-Revised (IES-R), the Hospital Anxiety and Depression Scale (HADS), the Mini-Mental State Examination (MMSE), the Center for Epidemiologic Studies Depression Scale (CES-D), the Short Form Beck Depression Inventory (BDI), and the Beck Anxiety Inventory (BAI).  **Detailed Findings**  ① **HRQoL Outcomes for ARDS Patients after VV-ECMO**  HRQoL scores were generally decreased. Compared to a matched general population, the mean SF-36 scale scores for VV-ECMO survivors were significantly lower.  Mobility problems were common among VV-ECMO patients.  A significant proportion (15%-54%) experienced mental health symptoms, specifically manifesting as anxiety, depression, and PTSD.  More than half of the ARDS patients treated with VV-ECMO returned to work.  ② **HRQoL Comparison**: VV-ECMO vs. Conventional Ventilation  Overall HRQoL outcomes were similar between the two groups.  The incidence of anxiety and depression was higher in the VV-ECMO group (36%-55%) compared to the conventional therapy group (27%-44%).  Conversely, the incidence of PTSD symptoms was higher in the conventional therapy group (44%) than in the VV-ECMO group (33%).  The reported rates of fatigue and decreased endurance were similar in both groups.  **Additional Insights & Considerations**  Gaining a deep understanding of patients' post-discharge health needs, physiological problems, psychological distress, and social adjustment challenges will help in developing more comprehensive follow-up treatment plans. Qualitative research can provide valuable insights into patient needs across various dimensions of quality of life.  When comparing HRQoL, it is also essential to consider the level of advanced care provided by the medical team during hospitalization, including psychological support and resources for nutrition, wound care, and physical therapy. | 1. Psychological Status - Existing Problems - Anxiety  2. Psychological Status - Existing Problems - Depression  3. Psychological Status - Existing Problems - Post-Traumatic Stress Disorder (PTSD)  4. Physiological Status - Motor Function and Musculoskeletal System - Muscle Function Status (Muscle Strength, Muscle Atrophy) |
| Literature 16: Grasselli G et al. (2019)  Study Type: Prospective Cohort Study  Quality Grade: High | Lung function, 6-minute walk test, quality of life (SF-36), PTSD | **Background:** Survivors of Acute Respiratory Distress Syndrome (ARDS) have long-term impairments in pulmonary function and health-related quality of life (HRQoL), but little is known about the outcomes for ARDS survivors who received treatment with extracorporeal membrane oxygenation (ECMO). The purpose of this study was to compare the long-term outcomes of ARDS patients who did and did not receive ECMO.  **Methods**: A prospective observational study of adult ARDS patients was conducted at a single center between January 2013 and December 2015. One year after hospital discharge, survivors underwent pulmonary function tests, chest computed tomography (CT), and completed HRQoL questionnaires.  **Results:** A total of 84 patients were studied (34 ECMO, 50 non-ECMO). The two groups had similar characteristics at baseline, but comorbidities were more common in the non-ECMO group (23 of 50 vs. 4 of 34, 46% vs. 12%; P < 0.001), and the severity of hypoxemia was greater in the ECMO group (median PaO2/FiO2 72 [interquartile range, 50 to 103] vs. 114 [87 to 133] mm Hg; P < 0.001). At one year, survival rates were similar (22/33 vs. 28/47, 66% vs. 59%; P = 0.52), and pulmonary function and CT scans were nearly normal in both groups. Non-ECMO patients had lower HRQoL scores and a higher incidence of post-traumatic stress disorder (PTSD).  **Conclusion:** Despite having more severe respiratory failure on admission, 1-year survival for ECMO patients did not differ from that of non-ECMO patients. Pulmonary function recovered nearly completely in each group, but non-ECMO patients had greater impairment in their health-related quality of life.  **Short-term Clinical Outcome Indicators:**  ICU survival rate, length of ICU stay, duration of mechanical ventilation and ECMO, frequency of tracheostomy, prone positioning and CRRT (Continuous Renal Replacement Therapy), total hospital length of stay, and in-hospital survival rate.  **One-Year Post-Discharge Follow-up**  Follow-up Method: Patients were contacted by telephone and invited to the center for on-site examinations.  **One-Year Follow-up Assessments:**  (1) Pulmonary function tests (including diffusing capacity for carbon monoxide), (2) resting arterial blood gas analysis, (3) 6-minute walk test, (4) chest CT scan, (5) HRQoL assessed using three questionnaires: the SF-36, St. George's Respiratory Questionnaire (SGRQ), and the Impact of Event Scale-Revised (IES-R).  **Follow-up Questionnaire Results:**  The quality of life of non-ECMO patients was significantly impaired. On the SF-36, the median scores for this patient group decreased by more than 5 points in all dimensions, indicating a clinically meaningful impairment in their quality of life; the difference was particularly significant in the emotional dimension (a decrease of over 30 points). Similarly, non-ECMO patients showed marked declines in quality of life impact dimensions such as activity and social function, and psychosocial disturbances (i.e., the "impacts" and "activity" dimensions of the SGRQ), rather than in the "symptoms" dimension (i.e., respiratory symptoms). Furthermore, the Impact of Event Scale (IES-R) showed that non-ECMO patients had a higher risk of post-traumatic stress disorder. | 1. Physiological Status - Respiratory System - Pulmonary Function Tests (Diffusing Capacity, etc.)  2. Physiological Status - Motor Function and Musculoskeletal System - 6-Minute Walk Test  3. Psychological Status - Existing Problems - Post-Traumatic Stress Disorder (PTSD) |
| Literature 17: Kanji HD et al. (2021)  Study Type: Retrospective Observational Study  Quality Grade: Moderate | Quality of life (EQ-5D), anxiety, depression, PTSD, pain and discomfort | **Objective:** Quality of life (QoL) outcomes for patients with Acute Respiratory Distress Syndrome (ARDS) treated with extracorporeal membrane oxygenation (ECMO) have been inconsistent. This study reports the QoL outcomes for a broad population of ARDS patients treated with modern modalities.  **Methods:** We prospectively recruited patients with ARDS who received ECMO at a quaternary care hospital in the United Kingdom between 2013 and 2015. We assessed their pulmonary function and QoL at 6 months post-admission using three QoL instruments: the EuroQol 5D (EQ-5D), the Hospital Anxiety and Depression Scale (HADS), and the Post-Traumatic Stress Syndrome 14-Questions Inventory (PTSS-14).  **Follow-up Time**: 6 months after hospital discharge.  **Follow-up Assessments**: Pulmonary function tests and chest X-ray.  **Follow-up Questionnaires:** EQ-5D, HADS (with a score of ≥11 as the cutoff for moderate-to-severe anxiety or depression), and PTSS-14.  **Follow-up Results:**  **A total of 43 patients were included in the analysis.**  ① At 6 months, pulmonary function tests were near normal.  ② The HADS showed that 32% and 11% of patients had moderate-to-severe anxiety and depression, respectively.  ③ The PTSS-14 indicated that 29% of patients showed signs of post-traumatic stress disorder (PTSD).  ④ The EQ-5D revealed that 67% of patients had difficulty returning to normal activities, 74% experienced some pain/discomfort, and none reported severe problems. A total of 77% of patients were able to return to work. No clinical or demographic variables were associated with poorer 6-month QoL.  **Conclusion:** Patients with ARDS treated with ECMO generally have favorable QoL outcomes, similar to those reported for patients not treated with ECMO. In terms of QoL, VV-ECMO is an effective treatment for patients with refractory ARDS. | 1. Psychological Status - Existing Problems - Anxiety  2. Psychological Status - Existing Problems - Depression  3. Psychological Status - Existing Problems - Post-Traumatic Stress Disorder (PTSD)  4. Physiological Status - General Condition - Pain Assessment |
| Literature 18: Ozgur MM et al. (2024)  Study Type: Retrospective Longitudinal Study  Quality Grade: Moderate | Cardiopulmonary Exercise Test (CPET), neurological sequelae, psychological status, rehabilitation training | **Study Overview and Patient Cohort**  A total of 29 patients who received VV-ECMO for COVID-19-related ARDS, were successfully weaned, and discharged were included in regular follow-up examinations. Of 35 patients who were successfully weaned, 30 were successfully discharged. The mean age of the patients was 37.1 years (±10.3), and 55% were male (16/29). The mean duration of ECMO support was 49.1 days (±22.3 days), with a 1-year survival rate after discharge of 100%. At the end of the 12-month follow-up, all patients had no mobility limitations. The VO2max score reached a high of 18.9 at the 12-month mark. The work return rate was 90%. Initiating rehabilitation training early and including patients in the ECMO center's follow-up plan not only aids their functional recovery but also promotes their successful reintegration into social life.  **Follow-up Protocol**  **① Follow-up Duration**: One-year follow-up results and cardiopulmonary exercise testing (CPET) data for discharged patients.  **② Follow-up Team**: Patients underwent meticulous follow-up through a comprehensive, multidisciplinary approach, including cardiovascular surgeons, intensive care specialists, pulmonologists, thoracic surgeons, infectious disease experts, cardiologists, physical therapists, and psychologists. This collaborative effort spanned the ICU, general wards, and continued post-discharge.  **③ Follow-up Schedule:** An ECMO coordinator was responsible for organizing patients' post-discharge treatment and follow-up plan. Patients received weekly follow-ups for the first two weeks after discharge, followed by monthly visits for the next three months, with subsequent follow-up appointments at 6 months and 1 year.  **④ Follow-up Method:** A combination of telephone calls and in-person interviews.  **⑤ Follow-up Intervention**: Patients were assessed by ECMO team members according to clinical protocols. Referrals to other departments were made when necessary. During follow-up, patients' medical conditions were reported to the ECMO committee. The assessment process included a comprehensive evaluation of their quality of life, covering social integration, employment status, occurrence of foot drop or other neuromuscular issues, neurological sequelae, tracheostomy and vocal cord-related complications, and mental health status.  **⑥ Follow-up Metrics:** Pulmonary function tests, cardiopulmonary exercise testing (CPET), and echocardiography.  **⑦ Recommended Follow-up Mechanism:**  Initiate multidisciplinary assessments from the early stages of treatment, start rehabilitation training as soon as possible, and encourage active participation in follow-up programs organized by the ECMO center. This integrated approach can effectively promote the patient's recovery process and facilitate their smooth return to social life.  **Rehabilitation Details**  The recovery of patients' mobility during long-term ECMO support and ICU stays remains a significant clinical challenge. The patient management strategy developed for this study emphasized providing rehabilitation support from the initial phase of ECMO therapy and promptly initiating early mobilization training after weaning. The post-discharge mobility rate for patients in this study was as high as 96.6%.  **(1) Rehabilitation During ECMO Support**  The rehabilitation plan was implemented by intensive care physicians, ECMO specialists, physical therapists, and nurses.  **For Sedated Patients:** Basic range of motion (ROM) exercises and bed positioning/turning were performed.  For Awake Patients (regardless of ECMO status): The plan focused on primary rehabilitation activities, including sensorimotor tasks, basic auditory and visual exercises, upper and lower limb ROM exercises, fine and gross motor coordination activities, and functional communication tasks.  **(2) Rehabilitation Post-ECMO and Post-Mechanical Ventilation Weaning**  Regardless of whether they had a tracheostomy or were in the ICU or a general ward, patients participated in bedside sitting exercises, standing exercises, followed by progression to sitting in a chair, stepping, and walking exercises.  **(3) Post-Discharge Rehabilitation Plan**  Patients received rehabilitation training three times a week for 8 weeks. The duration could be shortened to 4 weeks if the patient's overall condition was good. The plan primarily included: specialized training to strengthen upper and lower limb strength, basic breathing exercises (including diaphragmatic and pursed-lip breathing), and short-duration walking and cycling exercises.  **Definitions of Complications**  **Neurological Complications:** Primarily intracerebral hemorrhage and/or ischemic cerebrovascular events, diagnosed based on imaging methods and physical examination findings.  **Gastrointestinal Bleeding:** Defined as active bleeding discovered via endoscopy or direct examination, including symptoms like melena and hematemesis.  Pulmonary Complications: Diagnosed via imaging or bronchoscopy, including pneumothorax, hemothorax, alveolar hemorrhage, bronchial bleeding, and empyema.  Oral and Nasal Bleeding: Classified as oral or nasal bleeding requiring wound packing, typically performed by an otolaryngologist.  **Renal Complications**: Identified in patients who required continuous renal replacement therapy (CRRT) or permanent dialysis during or after ECMO support.  **Tracheal Complications:** Including bleeding in the tracheal area, formation of a tracheoesophageal fistula, or restenosis requiring intervention after tracheostomy removal.  **Psychiatric Issues:** Such as anxiety, delirium, and depression, as diagnosed by a psychiatrist. | 1. Physiological Status - Motor Function and Musculoskeletal System - Cardiopulmonary Exercise Test (CPET) or 1-Minute Sit-to-Stand Test  2. Physiological Status - Nervous System - Neurological Deficits (Motor, Sensory, Speech, Visual, Swallowing)  3. Psychological Status - Coping Strategies/Interventions - Etiology-Based Psychological Counseling  4. Living Status - Activities of Daily Living - Rehabilitation Training |
| Literature 19: Guenther SPW et al. (2023)  Study Type: Retrospective Cohort Study  Quality Grade: Moderate | Cognitive impairment, depression, PTSD, activities of daily living, cardiac function classification | **Objective:** This study evaluated the 6-month survival rate and recovery status of COVID-19 patients after receiving ECMO therapy.  **Methods:** A total of 60 patients received VV/VA-ECMO.  Results: 41.7% of patients were successfully weaned from ECMO, with a hospital discharge survival rate of 40.0% (24 patients). The use of vasopressor support and pre-ECMO SOFA scores were associated with non-survival. All patients over 65 years of age with a history of cancer, immunosuppression, chronic renal failure, or frailty died. At 6 months, 20 patients were alive (6-month survival rate of 33.3%, and 83.3% when calculated based on hospital discharge survivors), of whom 19 completed follow-up. Among these, 57.9% had no significant deficits, 26.3% had moderate deficits, and 15.8% had severe deficits. Cardiopulmonary status was good (exercise tolerance test: 84.2% were Class ≤2). 73.7% of patients had normal activities of daily living (ADL). Cognitive impairment was common (52.6%). 26.3% of patients experienced moderate depression, and 15.8% had post-traumatic stress disorder (PTSD). Social and work life were significantly impacted.  Conclusion: ECMO can be used as a rescue therapy for COVID-19, but relative contraindications such as advanced age, immunosuppression, a history of cancer, and frailty should be considered. Age, vasopressor support, and SOFA scores can aid in daily clinical decision-making. Deficits at 6 months are significant and highlight the need to focus on long-term rehabilitation.  **Follow-up Related Information**  **Follow-up Time**: 6 months after the initiation of ECMO therapy.  **Follow-up Method**: Telephone or email.  **Follow-up Content**: Health status, work status, and social quality of life.  **Assessment Scales:**  ①Modified Medical Research Council (mMRC) Dyspnea Scale: To assess the degree of dyspnea on a scale of 0-4 (0 = breathlessness only with strenuous exercise; 4 = too breathless to leave the house or when dressing).  ②New York Heart Association (NYHA) Classification: To assess cardiopulmonary function.  ③Montreal Cognitive Assessment (Telephone Version): To assess cognitive function.  ④Beck Depression Inventory-II (BDI-II): To assess the severity of depression.  ⑤Impact of Event Scale-Revised (IES-R): To screen for post-traumatic stress disorder.  ⑥Modified Rankin Scale (mRS): To assess the degree of dependency.  ⑦Health-Related Quality of Life (HRQoL): Assessed using the EQ-5D-5L questionnaire.  **Follow-up Recommendations**  ① As important as acute-phase survival is meaningful, long-term functional recovery. As the number of patients receiving ECMO increases, the importance of assessing potential persistent functional deficits and their management becomes more prominent, especially in the context of long-COVID related symptoms in patients with mild acute courses.  ② Healthcare institutions need to recognize that patients have ongoing medical needs after the critical care period. Tailored rehabilitation therapies and closer monitoring are required for those experiencing a prolonged recovery. Optimal long-term rehabilitation should become a focus of care.  ③ The current medical strategy should shift from solely focusing on acute-phase survival to emphasizing the goal of achieving meaningful long-term recovery. | 1. Physiological Status - Nervous System - Cognitive Function (ICU Post-Syndrome Screening)  2. Psychological Status - Existing Problems - Depression  3. Psychological Status - Existing Problems - Post-Traumatic Stress Disorder (PTSD)  4. Physiological Status - Circulatory System - Cardiac Insufficiency (Limb Edema, Fatigue, etc.) |
| Literature 20: Spangenberg T et al. (2018)  Study Type: Retrospective Cohort Study  Quality Grade: Moderate | Quality of life (SF-36), neurological function status (CPC), daily function | **Background:** Extracorporeal Cardiopulmonary Resuscitation (eCPR) can be considered a potential adjunct to conventional CPR (cCPR).  **Objective:** There is currently a lack of consensus standards to clearly define the indications for eCPR. This study is the first to conduct an in-depth analysis of the improvement in Health-Related Quality of Life (HRQoL) for patients treated with eCPR in a real-world medical setting.  **Methods:** A retrospective analysis of 60 consecutive patients who received eCPR between January 2014 and June 2016. One-year survival rates and HRQoL data for out-of-hospital cardiac arrest (OHCA) and in-hospital cardiac arrest (IHCA) were obtained using the SF-36 questionnaire.  **Results:** The mean duration of CPR before eCPR initiation was 66 ± 35 minutes, with 63.3% of cases being OHCA. Overall, 91.7% (n=55) of events were witnessed, and bystander CPR was performed in 73.3% (n=44) of cases. The primary cause of cardiac arrest was acute myocardial infarction (AMI, 66.7%), with an initial rhythm of ventricular fibrillation/ventricular tachycardia (VF/VT) being slightly more common (53.3%). The 12-month survival rate was 31%. Survivors were more likely to have received bystander CPR (p=0.001) and had a shorter duration of CPR (p=0.002). The HRQoL of intermediate-term survivors was decreased compared to a control group, but their scores were similar to the cardiac function and quality of life of subjects treated with ECMO for cardiogenic shock or respiratory failure.  **Conclusion**: Although the HRQoL scores of survivors in this study were significantly lower than the control group of patients on long-term hemodialysis, most of these differences improved when compared to patients who received ECMO for cardiogenic shock or respiratory failure. Therefore, the successful implementation of eCPR in appropriately selected patients can indeed significantly improve their quality of life, bringing it close to the HRQoL level of patients with chronic renal failure.  **Follow-up Related Information**  **Follow-up Time:** Patients were contacted starting at 6 months post-discharge, with data collected up to 12 months.  **Follow-up Scales:**  SF-36: Assesses eight dimensions: Physical Functioning (PF), Role-Physical (RP), Bodily Pain (BP), General Health (GH), Role-Emotional (RE), Social Functioning (SF), Mental Health (MH), and Vitality (VT).  Glasgow-Pittsburgh Cerebral Performance Categories (CPC) Scale: Used to assess neurological function status at the time of discharge.  **Follow-up Method:** A combination of face-to-face and telephone interviews.  This study found that factors such as age, location of cardiac arrest, and duration of low perfusion appear to further influence HRQoL. However, in the current context, our findings further reinforce the clinical value of eCPR.  **Follow-up Recommendations**  The impairment in emotional role functioning among survivors indicates their need for further rehabilitative support. Treating HRQoL as a core evaluation metric should guide future research—focusing on validating CPR-related variables that may influence the HRQoL of patients after eCPR. | 1. Physiological Status - Nervous System - Consciousness and Arousal Level  2. Living Status - Activities of Daily Living - Self-Care Ability  3. Physiological Status - Circulatory System - Palpitations |
| Literature 21: Chen KH et al. (2022)  Study Type: Cross-sectional Study  Quality Grade: Moderate | Quality of life (SF-36), anxiety, depression, employment status, self-perceived health | **Objective**: To assess the Health-Related Quality of Life (HRQoL) of adult patients who previously received ECMO and to explore the factors influencing HRQoL.  **Methods**: The study included patients discharged after ECMO treatment between April 2006 and April 2016. Data collection was conducted from October 2015 to October 2016 using structured questionnaires: the Hospital Anxiety and Depression Scale (HADS), the Impact of Event Scale-Revised (IES-R), and the Short Form-36 (SF-36v2). Predictors of physical and mental HRQoL were identified.  **Results:** After a median follow-up of 1060 days, the long-term survival rate was 28.6%. The mean scores for the physical and mental components of HRQoL were 46.32 and 50.39, respectively, indicating a low-to-moderate level of HRQoL. Employment status influenced all physical components of HRQoL. Depression was a major predictor for both physical and mental components. Self-perceived health status and anxiety were also factors influencing HRQoL.  **Conclusion**: Variables such as employment, self-perceived health status, and mental health affect HRQoL. Early assessment of these factors by healthcare professionals can allow for the integration of multidimensional interventions after discharge, which can improve the HRQoL of patients weaned from ECMO.  **Follow-up Background**  The focus of research in the field of critical care medicine has shifted from disease-oriented efficacy metrics (such as disease remission or improvement) to patient-centered comprehensive assessment indicators, covering the three dimensions of physical, psychological, and social well-being. Health-Related Quality of Life (HRQoL) is a crucial metric for evaluating the overall status of patients after critical illness recovery or treatment, as it reflects individual health elements that influence disease prognosis.  **Follow-up Assessment Forms**  ① Hospital Anxiety and Depression Scale (HADS): Used to assess the anxiety and depression status of subjects.  ② Impact of Event Scale-Revised (IES-R): A 22-item scale to assess symptoms related to post-traumatic stress disorder (PTSD). The scale uses statement items for subjects to describe their subjective feelings about past events.  ③ SF-36v2: Used to assess the physical and mental components of the patient's HRQoL.  **Health Predictors**  ① The most significant predictors of HRQoL were, in order: employment status (affecting all physical dimensions), self-perceived health (a predictor for role-physical and general health), and depressive symptoms (a major predictor for physical functioning, bodily pain, vitality, and social functioning).  **Follow-up Recommendations**  ① Patients who developed lower limb complications during ECMO treatment should be closely monitored for symptoms of anxiety and PTSD, as well as for potential reductions in physical HRQoL. Neurological injury can lead to limitations in daily function, which may contribute to depression.  ② Patients expressed a strong desire for wound care and limb rehabilitation to be a priority for improving their overall health. Therefore, incorporating physical rehabilitation measures into post-ECMO intervention programs can help enhance the physical dimensions of their HRQoL.  ③ For ECMO survivors, interventions should include continuous monitoring of anxiety and depression levels to enable timely measures to help alleviate symptoms.  ④ By identifying high-risk groups for mental health disorders and providing early interventions such as pharmacotherapy or cognitive-behavioral therapy (CBT), it is possible not only to reduce disease-related symptoms but also to improve long-term HRQoL and significantly enhance patients' quality of life.  ⑤ Providing emotional and psychological support as early as possible after the conclusion of ECMO therapy and continuing it post-discharge can help patients cope with anxiety and depression, as well as mitigate the impacts of employment status and self-perceived health.  ⑥ Informing patients about available resources after discharge—such as community support for adapting to a home environment, psychological teams for emotional support when needed, and encouraging patients to actively seek help through follow-up—can facilitate their recovery and reintegration. | 1. Psychological Status - Existing Problems - Anxiety  2. Psychological Status - Existing Problems - Depression  3. Living Status - Medical Adherence - Lifestyle Changes (Dietary Habits, Smoking Cessation, Alcohol Cessation, etc.)  4. Physiological Status - General Condition - Vital Signs |
| Literature 22: Oude Lansink-Hartgring A et al. (2023)  Study Type: Prospective Cohort Study  Quality Grade: High | Quality of life (EQ-5D), 1-year survival rate, follow-up costs | **Background:** To date, this is the largest study on the costs and Health-Related Quality of Life (HRQoL) of ECMO treatment. The study costs were calculated using a bottom-up, actual-cost approach, encompassing both societal expenditures and the economic impact at the individual patient level.  **Objective:** This study aimed to report the survival rate, Health-Related Quality of Life (HRQoL), and associated costs for the first year following extracorporeal membrane oxygenation (ECMO) treatment.  **Methods:** This was a prospective observational cohort study that included patients who received ECMO in the intensive care unit (ICU) between August 2017 and July 2019. We analyzed all medical costs incurred during the first year after the index admission. Follow-up included HRQoL analysis using the EQ-5D-5L questionnaire at 6 and 12 months.  **Results:** A total of 428 patients who received ECMO during their ICU stay were included. The one-year mortality rate was 50%. A total of 124 patients completed the 12-month follow-up. Survivors reported a good mean HRQoL (utility value) of 0.71 (scale range 0-1), with another reported value of 0.77. Their overall health status (Visual Analogue Scale, VAS, scale range 0-100) was reported as 73.6 at 12 months. The mean total cost in the first year was $204,513 ± $211,590, with hospitalization costs being the major component of the total cost. Follow-up costs were $53,752 ± $65,051, and costs due to absenteeism were $7,317 ± $17,036.  **Conclusion:** One year after an ECMO admission, the health-related quality of life is favorable, but the costs are high, though this may be acceptable considering the survival rate. However, our results are limited by loss to follow-up. Therefore, it is possible that only the patients with the best recovery returned their questionnaires. In real-life scenarios, this potential bias could lead to higher costs and poorer HRQoL.  **Follow-up Assessment Metrics**  ① Primary Outcome: Health-Related Quality of Life (HRQoL) one year after the start of ECMO treatment, assessed using the EQ-5D-5L questionnaire.  ② Secondary Outcomes: 1-year survival rate, EQ-5D Visual Analogue Scale (VAS) score, and costs.  Follow-up Time: At 6 and 12 months after the start of ECMO, patient HRQoL was measured using the EQ-5D-5L.  **Follow-up Method:** The same questionnaire was used to assess the patient's health status for the month prior to ICU admission. It was completed by the patient while conscious upon admission, or by a family member if necessary. Questionnaires were sent to patients via regular mail or email upon request. If no response was received, two reminders were sent.  **Follow-up Related Information**  ① To properly evaluate whether patients truly benefit after ECMO treatment and to reasonably assess the associated treatment costs, it is necessary to conduct long-term Health-Related Quality of Life (HRQoL) assessments. | 1. Living Status - Activities of Daily Living - Self-Care Ability  2. Physiological Status - General Condition - Vital Signs  3. Social and Family Support - Family Support System - Family Income/Impact |
| Literature 23: Chen KH et al. (2016)  Study Type: Qualitative Study  Quality Grade: High | Post-discharge problems and health needs, limb ischemia and neurological injury, anxiety, depression, social support | **Objective:** To explore the problems and health needs of adult ECMO patients within the first year after discharge.  **Background:** ECMO serves as life support during the treatment of advanced heart and respiratory failure. There is a lack of knowledge regarding the problems and health needs of adult patients discharged after receiving ECMO.  **Methods:** This study employed a qualitative descriptive interview design. Fourteen adult ECMO patients were recruited through purposive sampling before discharge. Data were generated through semi-structured, in-depth interviews conducted every three months post-discharge.  **Results:** Four themes described the post-discharge problems and needs: stress caused by the ECMO procedure; prioritizing health; support from family, friends, and healthcare professionals; and emotional support.  **Conclusion:** Multidisciplinary, evidence-based interventions should be implemented before or shortly after discharge to help address the physical, psychological, and social problems encountered by ECMO survivors, which can contribute to improving their quality of life.  **Follow-up Methodology**  **Follow-up Time and Method:** Semi-structured, face-to-face interviews. Each participant was interviewed four times at three-month intervals. The first interview was scheduled for three months after discharge. All interviews focused on the participants' post-discharge health problems and needs, following an open-ended, semi-structured interview guide. Each recorded interview lasted approximately 30-50 minutes.  **Summary of Follow-up Findings**  ① By systematically reviewing the multi-dimensional issues faced by patients post-discharge—including physical function, psychological state, and social adaptation—we can not only provide precise guidance for subsequent treatment plans but also help healthcare professionals develop more personalized care plans tailored to the characteristics of this patient population.  ② Stress Caused by the ECMO Procedure  The slow recovery stemming from the procedure persisted after discharge, leading to physiological discomfort. Participants commonly reported that this was both a physical and psychological burden, particularly pronounced during the first six months after discharge.  ③ Somatic Stress Responses Triggered by Physiological Discomfort  Nerve damage (in the leg, foot, groin) from ECMO cannulation led to an inability to squat to use the toilet.  ④ Psychological Stress Resulting from Physiological Discomfort  Hoarseness was a significant trigger for psychological stress after extubation. Four interviewees admitted to a fear of falling due to limb weakness. One patient experienced severe lower limb pain due to a chordoma and cardiac issues but was unable to participate in cardiac rehabilitation.  ⑤ Prioritizing Health  ⑥ Changing Previous Lifestyles  ⑦ Incorporating Complementary and Alternative Medicine (CAM)  ⑧ Self-Motivation for Successful Recovery  ⑨ Support from Family, Friends, and Healthcare Professionals  ⑩ Emotional Adaptation  Family support was found to have a positive impact on patients' recovery responses—not only boosting morale but also promoting the improvement of self-care behaviors. Including the experiences of patients' family members in future qualitative research could provide more insights into the home life experiences of ECMO survivors.  **Follow-up Recommendations**  To address the health problems and needs of post-ECMO patients, several effective strategies are proposed:  ① For patients with lower limb ischemia or neurological complications, the following measures are recommended:  Educate patients on how to monitor and assess the "6 P's" (Pain, Pallor, Pulselessness, Paresthesia, Poikilothermia, Paralysis) and encourage them to report relevant information to healthcare staff promptly.  Arrange for physical therapy and/or medication to improve mobility.  ② To comprehensively improve the physical and mental health of ECMO patients and promote the recovery process, it is recommended that healthcare professionals take the following measures: first, guide patients in strategies to cope with anxiety and depression to reduce psychological distress; second, encourage patients to participate in a systematic exercise rehabilitation program; and finally, provide education about Complementary and Alternative Medicine (CAM).  ③ The findings of this study provide an effective framework for healthcare workers—by providing physical therapy, enhancing patients' self-management skills, and offering psychological support to patients at risk of anxiety, depression, or PTSD—to help patients transitioning from the ICU quickly adapt to changes in physical function and their psychosocial environment. | 1. Physiological Status - ECMO Catheter Site and Vascular Complications - Limb Ischemia or Compartment Syndrome  2. Psychological Status - Existing Problems - Anxiety  3. Psychological Status - Existing Problems - Depression  4. Social and Family Support - Family Support System - Needs of Fixed/Rotating Family Caregivers |
| Literature 24: Wang F et al. (2024)  Study Type: Qualitative Study  Quality Grade: High | Changes in physical function, changes in psychological state, adaptation to life, demand for rehabilitation | **Objective:** To explore the life experiences of patients discharged after receiving extracorporeal membrane oxygenation (ECMO) support.  **Design:** A qualitative descriptive approach was used.  **Methods:** Patients who had received ECMO support and been discharged were recruited. Thirteen participants took part in this study. Data were collected through semi-structured interviews and analyzed using the Colaizzi method.  **Findings**: Participants reported four main themes regarding their life experiences: changes in physical function, changes in psychological state, positive adaptation to daily life, and a significant need for rehabilitation.  **Conclusion:** Differentiated, continuous, and accessible post-discharge interventions—including physical and psychological care, social support, spiritual support, and rehabilitation services—should be provided according to the patient's condition. We also call on the government to increase the reimbursement rate for ECMO treatment for patients. These measures may help improve patients' quality of life.  **Follow-up Recommendations**  ① Healthcare professionals should pay attention to the physical and psychological complications and residual effects in patients after ECMO support and provide better long-term care after discharge.  The lives of patients after ECMO support are often plagued by complications and sequelae. For example, VA-ECMO can lead to lower limb ischemia, affecting muscle and nerve function in the legs. Patients treated with VV-ECMO may experience shortness of breath, which is related to incomplete recovery of lung function from the primary disease.  ② Healthcare professionals should provide targeted psychological intervention plans based on the individual situation of each patient. At the same time, we call on the government to strengthen insurance support for ECMO consumables to reduce the financial burden on patients.  Post-ECMO patients endure immense stress; most experienced a life-threatening situation in the ICU and expressed a fear of death. Increased financial burdens and difficulties in returning to work are primary reasons for the significant stress patients face after discharge.  ③ It is recommended that healthcare professionals make good use of patients' positive mindset and provide more comprehensive professional guidance during the rehabilitation process. This includes systematically explaining various symptoms, recommending personalized exercise plans, and offering dietary advice.  ④ A multi-dimensional support system encompassing medical care, nursing, rehabilitation therapy, and psychological counseling plays a significant role in promoting the health of post-ECMO patients.  ⑤ In addition to encouraging ECMO patients to seek social support from family, friends, and peer support groups, healthcare professionals should also provide spiritual care according to the patient's religious and cultural needs.  ⑥ It is recommended that managers of medical institutions should develop comprehensive care plans:  Implement differentiated interventions (lasting 6-12 months) based on patients' underlying diseases and ECMO mode, with a focus on patient groups with mental health issues. Provide systematic care through multi-dimensional social support, spiritual care, professional guidance, and accessible rehabilitation services. Simultaneously, call on the government to increase the medical insurance reimbursement ratio for ECMO treatment. These initiatives will effectively improve the quality of life (QoL) for patients receiving ECMO therapy. | 1. Physiological Status - Motor Function and Musculoskeletal System - Functional Activity Disorders (Difficulty Climbing Stairs, Inability to Stand, etc.)  2. Psychological Status - Existing Problems - Anxiety  3. Living Status - Activities of Daily Living - Self-Care Ability  4. Living Status - Activities of Daily Living - Rehabilitation Training |
| Literature 25: Hsieh FT et al. (2016)  Study Type: Cross-sectional Study  Quality Grade: Moderate | Quality of life (SF-36), activities of daily living (Barthel Index, IADL) | **Objective:** To understand the factors that influence the quality of life of adult patients treated with ECMO.  **Methods:** Data were collected from a convenience sample of adult patients who received ECMO treatment between 2009 and 2011. A structured questionnaire was used to collect health-related data, with the SF-36 used to assess quality of life.  **Results:** The mean age of the 100 participants was 48.95 years. Two health status indicators showed a significant positive correlation with quality of life: the Barthel Index and the Instrumental Activities of Daily Living (IADL) scale. Two other indicators showed a significant negative correlation: the Charlson Comorbidity Index and Part II of the Nottingham Health Profile (NHP-II). The mean scores for the physical and mental component summary scores of the SF-36 were 49.25 and 48.13, respectively. These component scores were both significantly and negatively correlated with Part II of the Nottingham Health Profile. Stepwise multiple linear regression analysis indicated that the number of life areas affected, as measured by the Nottingham Health Profile-Part II, was a common factor influencing both the psychological and physical component summary scores of quality of life.  **Conclusion:** Social participation is a common factor influencing quality of life. By observing the health status and quality of life of ECMO patients, nurses can identify interventions to effectively improve health-related quality of life.  Quality of life is defined as the subjective satisfaction with physiological, psychological, and self-actualization needs. In the past, medical treatment focused more on prognosis and survival rates, but now it also considers whether patients can return to their previous lifestyle. Therefore, quality of life has become an important indicator when assessing a patient's ability to reintegrate into society.  **Follow-up Methodology**  **Follow-up Method**: Health data were collected from participants through telephone interviews and questionnaires.  **Follow-up Survey Content:**  ①Demographic Questionnaire  ②Charlson Comorbidity Index (CCI): To measure physical function and structure.  ③Barthel Index (BI): To assess the degree of disability.  Instrumental Activities of Daily Living (IADL) Scale: To assess the level of dependency.  ④Nottingham Health Profile-Part II (NHP-II): To assess the degree of social participation.  ⑤Short Form-36 (SF-36): To assess quality of life.  **Follow-up Recommendations**  ① Healthcare professionals should encourage health monitoring and self-management skills to enable patients to cope with their illness and maintain good health.  ② It is recommended to initiate patient rehabilitation plans early to maintain sufficient physical activity for preserving independence in daily living. Simultaneously, family members should be encouraged to understand the importance of rehabilitation training.  ③ Early rehabilitation or vocational recovery is a method to reduce the time and energy required for patients to return to normal life and to improve their quality of life.  ④ Healthcare professionals should provide or refer patients for vocational rehabilitation services to help them make full use of available resources. Starting rehabilitation training during hospitalization can help them return to the workplace and resume social interactions, a practice that helps enhance social participation and thereby improve quality of life.  ⑤ During ECMO: Nurses should initiate early mobilization and rehabilitation training. These measures should be started during the ICU stay and continued throughout the entire hospitalization period.  ⑥ Post-ECMO Discharge: Implement continuous post-ECMO follow-up and provide rehabilitation guidance and referral information. | 1. Living Status - Activities of Daily Living - Self-Care Ability  2. Physiological Status - Motor Function and Musculoskeletal System - Muscle Function Status (Muscle Strength, Muscle Atrophy)  3. Social and Family Support - Social Interaction and Network |
| Literature 26: Ayers B et al. (2021)  Study Type: Retrospective Cohort Study  Quality Grade: Moderate | Long-term renal function, dialysis needs, multiple organ dysfunction | **Methods**: We aimed to utilize over 208 patient-years of follow-up data from our large institutional cohort of VA-ECMO patients to determine the incidence of the need for renal replacement therapy (long-term dialysis, LT-dialysis) after discharge in patients who received VA-ECMO support. This retrospective study included all adult VA-ECMO patients at our institution from January 2014 to October 2018 (N = 283).  **Results**: Among the 99 survivors (35%), 88 (89%) did not require long-term dialysis of any duration after discharge from the index hospitalization. Patients who required VA-ECMO for decompensated cardiogenic shock were more likely to need long-term dialysis (p = .034). Furthermore, those who required renal replacement therapy during VA-ECMO (N = 27) also had a higher incidence of long-term dialysis (33%).  **Conclusion:** Among survivors of VA-ECMO support, the incidence of long-term dialysis dependence is low. Concerns about potential long-term adverse effects of VA-ECMO should not preclude patients from receiving this life-saving support.  **Additional Findings and Recommendations**  **Indications and Duration of VA-ECMO Support**  Although ECMO support can lead to temporary, severe multi-organ failure, the vast majority of VA-ECMO survivors are expected to recover to near-normal renal function.  Renal failure requiring CRRT during ECMO therapy has been shown to be closely associated with poorer overall outcomes, including an up to four-fold increase in mortality compared to patients who do not require CRRT.  Patients who required LT-dialysis had a higher mortality rate (45%), suggesting a fragile clinical state. However, most of these patients survived for more than a year after ECMO decannulation. Multi-system organ failure was the most common cause of eventual death. These patients require close follow-up by a coordinated, multidisciplinary team to proactively manage their underlying organ dysfunction, including referral for multi-organ transplantation when applicable. | 1. Physiological Status - Urinary System - Urine Volume and Characteristics (Hematuria, Proteinuria)  2. Physiological Status - Urinary System - Use of Diuretics  3. Physiological Status - Urinary System - Blood Purification (Optional) |
| Literature 27: Lansink-Hartgring AO et al. (2017)  Study Type: Retrospective Cohort Study  Quality Grade: Moderate | Quality of life (EQ-5D), 6-minute walk test, lung function | **Objective:** To summarize clinical experience, with a primary focus on evaluating patients' health-related quality of life (HRQoL). Secondary outcome measures included length of ICU stay, total hospital days, and mortality before and after transplantation.  **Methods**: A retrospective analysis of data from all adult patients treated with Extracorporeal Life Support (ECLS) between 2010 and 2014, compared with a contemporaneous cohort of adult patients who underwent double-lung transplantation. For the ECLS group, the European Quality of Life Scale (EQ-5D) was used to assess their overall health status.  **Results:** A total of 130 double-lung transplant surgeries were performed, with 9 of these cases being completed in patients after ECLS therapy. An additional 11 patients died while receiving ECLS while awaiting transplantation. From the patient's perspective, the quality of life at 12 months post-operation was comparable between the two groups, with a median score of 80 on the Visual Analogue Scale (VAS). The median (Interquartile Range [IQR]) score on the EQ-5D self-reported questionnaire, assessed from a societal perspective, was 0.73 (0.5–0.9) for the ECLS group. In terms of length of ICU stay, the ECLS group had a median of 25 days (9–68 days) compared to 7 days (4–18 days) for the control group (P=0.001). The total hospital stays were 66 days (40–114 days) and 42 days (29–62 days), respectively (P=0.004).  **Conclusion:** ECLS can be used as a bridge to lung transplantation. However, for various reasons, a considerable number of subjects were not successfully bridged. Despite longer ICU and hospital stays, patients who were successfully transplanted after ECLS had outcomes comparable to the general lung transplant population in terms of quality of life, lung function, exercise test results, and mortality.  **Follow-up Methodology**  **Follow-up Time:** At 12 months.  **Follow-up Method:** Telephone interview.  **Follow-up Assessments:**  ① Primary Outcome Measures: European Quality of Life-5 Dimensions (EQ-5D) and Visual Analogue Scale (VAS).  ② Secondary Outcome Measures: Length of ICU stay, use of blood products, primary graft dysfunction (PGD), lung function at 12 months post-transplant (FEV1), exercise test results (6-minute walk test), and mortality.  **Follow-up Recommendations**  ① Rehabilitation after critical illness is achieved through measures implemented during the ICU stay (e.g., minimizing sedation and using awake ECLS strategies).  ② Comprehensive attention to the five dimensions of the EQ-5D (mobility, self-care, usual activities, pain/discomfort, and anxiety/depression) will significantly enhance the health-related quality of life for lung transplant patients.  ③ Physical rehabilitation is crucial for patients' early recovery of HRQoL post-surgery. This is even more critical for patients treated with ECLS as a bridge to transplantation, as they often experience longer hospital stays and suffer from muscle weakness and atrophy. | 1. Physiological Status - Motor Function and Musculoskeletal System - 6-Minute Walk Test  2. Physiological Status - Respiratory System - Pulmonary Function Tests (FEV1, etc.)  3. Living Status - Activities of Daily Living - Self-Care Ability |
| Literature 28: O'Brien SG et al. (2020)  Study Type: Retrospective Cross-sectional Study  Quality Grade: Moderate | Quality of life (SF-36), anxiety, depression, PTSD, return to work | **Objective**: To assess the long-term health-related quality of life (HRQoL) of adult patients in Ireland who received V-V ECMO for Acute Respiratory Distress Syndrome (ARDS).  **Methods:** Through a retrospective cross-sectional survey, self-reported quality of life data were collected from V-V ECMO survivors who had been discharged from the ICU for ≥6 months. Between 2009 and 2013, 29 patients with respiratory failure received V-V ECMO, of whom 19 (66%) were successfully discharged and participated in the study. The mean age of the subjects was 44 ± 11 years, and 7 were male. The median follow-up time was 36 (14–39) months. Study participants reported a decline in physical health scores compared to an age- and sex-matched Irish general population, while their mental health status was similar to that of the matched population.  **Results**: 54% of participants exhibited symptoms of anxiety, 15% had symptoms of depression, and 23% were at risk for post-traumatic stress disorder (PTSD). Of the participants who were previously employed, 67% had returned to work.  **Conclusion**: The physical and psychological recovery process for ARDS patients is prolonged, even three years after receiving V-V ECMO.  **Follow-up Methodology**  **Follow-up Method:** Initial contact was made via email, followed by telephone interviews. Each interview lasted approximately 30 minutes. To minimize inter-interviewer variability and the risk of error, all questions were systematically read by a single researcher according to the questionnaire design, avoiding in-depth follow-up questions. This approach reduced the burden on respondents, ensured standardized administration of the questionnaire, and enhanced data reliability.  **Follow-up Assessments:**  SF-36: To assess overall health-related quality of life.  Hospital Anxiety and Depression Scale (HADS): To assess symptoms of anxiety and depression.  Impact of Event Scale-Revised (IES-R): To assess the risk of PTSD.  **Follow-up Recommendations**  ① Future research on long-term HRQoL should include multicenter data to identify modifiable factors that can alleviate the physical and psychological burden on patients and their families/caregivers.  ② Early research on ARDS patients post-ICU treatment primarily focused on survival rates or pulmonary function recovery. There is a growing awareness of the long-term physical and psychological challenges patients face during recovery. Despite near-normalization of lung function, persistent physical weakness remains a significant feature of the ARDS recovery period.  ③ Survivors may face multidimensional limitations, including impairments in physical function, social ability, emotional state, or neurocognitive function.  ④ The causes of long-term physical disability are often difficult to pinpoint. The near-universal presence of ICU-acquired weakness and potential ECMO-specific complications are likely significant contributors to long-term physical disability.  ⑤ It is recommended that dedicated ICU outpatient follow-up clinics be established to assist post-ECMO patients and their families with their rehabilitation. | 1. Psychological Status - Existing Problems - Anxiety  2. Psychological Status - Existing Problems - Depression  3. Psychological Status - Existing Problems - Post-Traumatic Stress Disorder (PTSD)  4. Living Status - Medical Adherence - Follow-up Adherence |
| Literature 29: Harley O et al. (2020)  Study Type: Retrospective Cohort Study  Quality Grade: Moderate | Quality of life (SF-36), PTSD, long-term survival | **Methods**: A single-center retrospective cohort study of patients who received ECMO support between 2012 and 2016. Survivors completed the SF-36 questionnaire and the PTSD Checklist-Civilian Version (PCL-C). A total of 241 patients (52 years old, 158 male) received ECMO. One hundred fifty-one patients (62.7%) survived to discharge, of whom 129 (85%) were alive at a median follow-up of 31.8 months. The median survival time was 56.6 months. Seventy-six people (58.9%) returned complete surveys.  **Results:** The ECMO cohort demonstrated a decreased Quality of Life (QoL) in all domains, which was significantly correlated with a high risk of PTSD, with 30.8% falling into the highest-risk category for PTSD.  **Conclusion:** The need for Renal Replacement Therapy (RRT) and ECMO duration were significantly associated with increased mortality but not with quality of life. A diagnosis of primary graft dysfunction or respiratory failure was independently associated with better long-term survival, but there was no difference in quality of life between different underlying diseases. Despite good long-term survival, decreased quality of life and post-traumatic stress disorder are frequently observed. These findings reaffirm the need for long-term follow-up and rehabilitation for this population.  **Follow-up Related Information**  ① Assessing long-term outcomes and health-related quality of life is both a responsibility to ensure that clinical ethical principles (such as beneficence) are upheld and is aligned with the patient-centered care philosophy. This aligns closely with the concepts of Patient-Reported Outcome Measures (PROMs), Patient-Reported Experience Measures (PREMs), and Patient-Centered Outcome Measures (PCOMs).  **Follow-up Method:** Telephone contact, with questionnaires completed online or via mail.  **Follow-up Assessment Forms**: SF-36 questionnaire, PTSD Checklist-Civilian Version (PCL-C).  **Predictive Factors:**  ① The need for RRT during ECMO therapy is the strongest predictor of patient mortality and will have a significant impact on long-term survival.  ② ECMO duration increases the risk of mortality, reflecting both disease severity and an increased likelihood of potential complications (such as chronic critical illness syndrome, bleeding, or infection).  **It is particularly important to provide younger patients with long-term follow-up programs that include both physical and psychosocial rehabilitation.**  **PTSD-Related Descriptions and Recommendations**  PTSD has a negative impact on all tested dimensions of quality of life. Specific risk factors, such as delirium and prolonged sedation, may exacerbate this process. A diagnosis of PTSD following ICU treatment may be related to the patient's perception of chronic illness and the degree of exposure to the ICU environment. To reduce its incidence, the underlying mechanisms linking invasive organ support, sedation, and other interventions need to be clarified. Future research should employ more comprehensive PTSD assessment tools to deeply analyze this complex interplay. Follow-up screening for PTSD and early psychological intervention are equally essential; neither can be neglected.  It is recommended to implement more intensive physical rehabilitation in an ICU outpatient clinic, focusing on patient-reported physical and psychosocial issues that affect quality of life. Future research should evaluate the impact of such interventions on this vulnerable patient population. | 1. Psychological Status - Existing Problems - Post-Traumatic Stress Disorder (PTSD)  2. Living Status - Activities of Daily Living - Self-Care Ability  3. Physiological Status - General Condition - Vital Signs |
| Literature 30: Knudson KA et al. (2019)  Study Type: Systematic Review  Quality Grade: High | Long-term quality of life, anxiety, depression, PTSD, physical function disorders, social function | **Objective**: To synthesize studies on the long-term (at least 6 months post-ECMO) HRQoL of adult patients who received ECMO treatment.  **Methods:** In this comprehensive review, we searched three electronic databases and manually searched relevant journals for literature published between 2000 and 2019, in accordance with the Preferred Reporting Items for Systematic Reviews and Meta-Analyses (PRISMA) guidelines.  **Results:** Thirty-one studies involving 913 patients who received extracorporeal membrane oxygenation (ECMO) were included. Patients in the V-V ECMO group had slightly better long-term HRQoL than those in the V-A group, and mental health scores were generally better than physical health scores. Although an improving trend was observed during the follow-up period, survivors frequently experienced physical complications, functional limitations, anxiety, depression, and symptoms of post-traumatic stress disorder.  **Conclusion**: The findings indicate that early identification and management of physical and psychological problems may help improve HRQoL outcomes.  **Follow-up Assessment Tools**  ①Long-term HRQoL outcomes were assessed using both generic and disease-specific HRQoL instruments.  ②Generic Instruments: SF-36, SF-12, EQ-5D  ③Disease-Specific Instruments: St. George's Respiratory Questionnaire (SGRQ), Airways Questionnaire 20-revised (AQ20-R), and Kansas City Cardiomyopathy Questionnaire (KCCQ)  **Key Findings from Follow-up Assessments:**  ① Comparison by ECMO Type  Overall scores (assessed by SF-36 or SF-12 PCS, MCS, and EQ-5D VAS) showed that the V-V cohort performed slightly better than the V-A group.  In the SF-36 dimensions, the highest scores (indicating less pain) for the Bodily Pain domain were found in the ECPR, mixed-type, and V-A samples. The Role-Emotional domain performed best in the mixed-type, V-A, and V-V samples, and the Social Functioning dimension was most prominent in the mixed-type, V-A, and V-V samples.  ② Outcomes after Bridging to Heart Transplantation  The largest difference between groups was in the Role-Emotional domain (which was better in transplant recipients).  ③ Comparison with Non-ECMO Populations  In most cases, patients who received ECMO treatment had better HRQoL.  ④ Changes in HRQoL Over Time  HRQoL metrics showed a trend of improvement over time. From discharge to 12 months, the Physical Functioning, Physical Role, and Emotional Role domains of the SF-36, as well as the SF-36 physical function scores and the EQ-5D self-care and usual activities dimensions, all showed significant improvement.  Patients receiving ECMO are routinely and extensively exposed in the ICU to nearly all known risk factors for PTSD, including prolonged mechanical ventilation and exposure to certain specific medications, especially opioids, benzodiazepines, and catecholamines.  **Implications for Clinical Practice**  ① Persistent health issues leading to decreased health-related quality of life are common, yet according to clinical experience, routine screening for these problems remains inadequate.  ② Assessing HRQoL may provide crucial insights into the perceived health status of post-ECMO patients.  ③ There is an urgent need to strengthen screening for anxiety, depression, and PTSD, and to refer patients to mental health counseling services as needed. The long-term care for these patients should also include enhanced physical rehabilitation strategies, along with proper management of pain and discomfort.  ④ By establishing interdisciplinary collaboration mechanisms (e.g., integrating nurses, physicians, social workers, clinical pharmacists, and chaplains), long-term, comprehensive medical care services can be provided to patients after ICU recovery.  **Implications for Future Research**  ① To guide clinical practice, further research on long-term HRQoL is necessary. Currently, data are particularly scarce for elderly patients, women, or populations receiving ECPR. Larger sample sizes are needed to conduct more meaningful statistical analyses.  ② Longitudinal studies—especially those including baseline HRQoL data—can reveal the differences in patients' HRQoL before and after ECMO treatment.  ③ A core outcome set for ECMO patients was recently proposed, encompassing three areas: adverse events (e.g., intracranial and major bleeding), mortality, and life impact (including HRQoL, neurological recovery, disability, activities of daily living, and return to work).  ④ There is strong support for the standardization of outcome reporting for this population, which will facilitate cross-study data sharing and enhance the generalizability of research findings. | 1. Psychological Status - Existing Problems - Anxiety  2. Psychological Status - Existing Problems - Depression  3. Psychological Status - Existing Problems - Post-Traumatic Stress Disorder (PTSD)  4. Physiological Status - Motor Function and Musculoskeletal System - Functional Activity Disorders  5. Social and Family Support - Social Interaction and Network |
| Literature 31: Dardik G et al. (2024)  Study Type: Retrospective Cohort Study  Quality Grade: Moderate | Recurrence of heart failure, long-term survival, cardiac function (EF value) | **Objective**: To investigate the long-term outcomes of patients with cardiogenic shock who were discharged without receiving heart replacement therapy (HRT) after treatment with V-A ECMO.  **Methods:** A retrospective analysis of 615 patients with cardiogenic shock who received V-A ECMO at our institution between January 2015 and July 2021. Of these, 166 patients (27.0%) who did not receive HRT were included in the study. Baseline characteristics, discharge laboratory values, vital signs, electrocardiograms, and echocardiogram results were evaluated. Follow-up was conducted to determine survival status, with the primary outcome measure being post-discharge mortality.  **Results**: Among the 166 patients, 158 (95.2%) had post-discharge follow-up, with a median follow-up time of 2 years. The median ejection fraction (EF) at discharge was 52.5% (IQR: 32.5-57.5). At discharge, 92 patients (56%) were on beta-blockers, 28 (17%) were on ACE inhibitors, ARBs, or ARNIs, and 50 (30%) were using loop diuretics. Kaplan-Meier analysis showed a 1-year survival rate of 85.6% (95% CI: 80.1%-91.2%) and a 5-year survival rate of 60.6% (95% CI: 49.9%-71.3%). The Cox regression model revealed that a history of congestive heart failure (CHF) was a strong predictor of increased mortality (HR=1.929; p=0.036), whereas discharge EF value and the etiology of VA-ECLS were not associated with increased post-discharge mortality.  **Conclusion**: Patients who are discharged after full myocardial recovery following VA-ECMO support, without receiving HRT, require close outpatient follow-up due to the risk of recurrent heart failure and increased mortality.  **Follow-up Methodology**  **Follow-up Method**: Surveys and informed consent forms were sent via telephone or email.  **Follow-up Results and Recommendations**  ① Patients discharged after recovering from VA-ECMO face a high risk of recurrent heart failure and mortality. This patient population requires close monitoring, timely treatment, and regular follow-up by heart failure specialists.  ② Close follow-up is essential to achieve optimal treatment outcomes, which includes enabling myocardial recovery and/or ensuring timely referral for heart replacement therapy.  ③ Post-discharge care for VA-ECMO survivors often involves multidisciplinary collaboration and requires the participation of multiple different teams. Therefore, a comprehensive discharge summary is crucial. There is an urgent need to develop standardized post-discharge management guidelines for this population and to raise public awareness of this issue.  ④ All patients should be evaluated by a heart failure specialist before discharge and, if necessary, receive a guideline-directed medical therapy (GDMT) regimen. Therefore, the low utilization rate of neurohormonal blockers may be related to patient tolerance to these medications. Further research is needed to systematically evaluate the actual use of neurohormonal blockers during post-discharge follow-up to determine their impact on long-term myocardial recovery.  ⑤ Compared to the LVEF metric, a history of congestive heart failure may be a more effective indicator for identifying patients who require more intensive, long-term follow-up and management. | 1. Physiological Status - Circulatory System - Cardiac Insufficiency (Limb Edema, Fatigue, etc.)  2. Physiological Status - Circulatory System - Use of Cardiovascular Drugs (Antiarrhythmic, Cardiotonic, etc.)  3. Physiological Status - Circulatory System - Palpitations |
| Literature 32: Turgeon J et al. (2024)  Study Type: Systematic Review and Meta-analysis  Quality Grade: High | Quality of life (SF-36), cognitive function, functional status, return to work | **Methods**: We searched electronic databases from their inception up to January 17, 2023. We screened for clinical trials and observational studies that reported long-term outcomes of patients with ARDS treated with ECMO. Health-related quality of life (HRQoL) was the primary outcome measure. Secondary outcomes included cognitive function, mental health, functional status, respiratory symptoms, and return to work.  **Results**: From 7,126 screened articles, one randomized clinical trial and 31 observational studies were ultimately included, with 7 of these studies comparing conventional mechanical ventilation (CMV) with ECMO. The overall quality of the included studies was limited, with most being of low (45%) or moderate (32%) quality. There was no significant difference in HRQoL, as measured by the SF-36 scale, between ECMO and CMV patients (Physical Component Summary [PCS]: Mean Difference 3.91 [-6.22 to 14.95]; Mental Component Summary [MCS]: Mean Difference 1.33 [-3.93 to 6.60]). There were no differences between the groups in cognitive function, mental health, functional status, and respiratory symptoms, but the available data for comparison were limited. ECMO survivors had a high rate of disability, with only 49% of patients returning to work and 23% requiring home care during the follow-up period.  **Conclusion:** Survivors of ARDS treated with ECMO experience significant functional impairment across multiple domains. Further research is needed to compare the long-term effects of ECMO versus CMV on patient outcomes.  **Key Findings and Recommendations**  A large number of survivors experience issues such as decreased health-related quality of life, mental health symptoms, and cognitive impairment, which prevent them from returning to work. This important finding must be given serious consideration when discussing ECMO support options with patients and their caregivers.  Long-term cognitive outcomes should be incorporated into follow-up metrics to assess the impact of ECMO on cognitive function.  Resource-intensive interventions can only be provided in specialized centers and have limited capacity. The evaluation of future extracorporeal support should include long-term outcomes as a key metric to ensure that any potential positive impact on mortality is not offset by a significant decline in the quality of life of survivors. | 1. Physiological Status - Nervous System - Cognitive Function (ICU Post-Syndrome Screening)  2. Living Status - Activities of Daily Living - Self-Care Ability  3. Living Status - Medical Adherence - Follow-up Adherence |
| Literature 33: von Bahr V et al. (2019)  Study Type: Retrospective Cohort Study  Quality Grade: Moderate | Lung function, quality of life (SF-36/SGRQ), anxiety, depression, 6-minute walk test | **Objective:** To assess the long-term complications in ARDS survivors treated with ECMO over an extremely long-term (≥3 years) period, including pulmonary function and morphological damage, decreased quality of life, mood disorders, limited walking ability, and return-to-work status.  **Methods:** A single-center retrospective cohort study of long-term survivors treated with extracorporeal membrane oxygenation for respiratory failure between 1995 and 2010 at a Swedish tertiary referral center. Eligible patients were contacted, and those who agreed to participate were interviewed and surveyed during a one-day hospital visit.  **Results:** A total of 38 patients were included, with a median follow-up time of 9.0 years. Consistent with previous research on conventionally managed ARDS survivors, all dimensions of the SF-36 and St. George's Respiratory Questionnaire (SGRQ) showed a decreased quality of life. 47% of patients exhibited reduced diffusing capacity for carbon monoxide, and 82% had some degree of residual lung parenchymal abnormalities. The presence of parenchymal lung disease was positively correlated with decreased quality of life and reduced diffusing capacity. Additionally, 22% and 14% of patients reported symptoms of anxiety and depression, respectively.  **Conclusion:** Similar to ARDS survivors treated with conventional therapy, a significant long-term burden persists even 3-17 years after ECMO treatment. Future prospective studies are needed to elucidate the risk factors for these sequelae.  **Follow-up Methodology**  **Follow-up Assessment Scales:**  Patient-reported quality of life was assessed using the SF-36 and the St. George's Respiratory Questionnaire (SGRQ).  Anxiety and depression were assessed using the Hospital Anxiety and Depression Scale (HADS).  Symptoms of Post-Traumatic Stress Disorder (PTSD) were assessed using a trauma screening questionnaire.  **Follow-up Examinations:**  Clinical consultation  Chest CT scan  Pulmonary function tests (PFTs)  6-minute walk test (6MWT)  **Additional Insights and Recommendations**  For the SF-36 and SGRQ scales, studies have reported the Minimal Clinically Important Difference (MCID), which represents the smallest change in score that a patient or clinician would consider meaningful. | 1. Physiological Status - Respiratory System - Pulmonary Function Tests (Diffusing Capacity, etc.)  2. Physiological Status - Motor Function and Musculoskeletal System - 6-Minute Walk Test  3. Psychological Status - Existing Problems - Anxiety  4. Psychological Status - Existing Problems - Depression |
| Literature 34: Wilcox ME et al. (2020)  Study Type: Systematic Review and Meta-analysis  Quality Grade: High | Quality of life (SF-36), psychological disorders, muscle weakness, residual lung injury | **Objective:** As Extracorporeal Membrane Oxygenation (ECMO) has become a mainstream treatment for Acute Respiratory Failure (ARF), its impact on the long-term prognosis of critically ill adult patients warrants in-depth investigation.  **Methods:** Databases were searched from 1948 to November 30, 2016, to select controlled trials and observational studies focusing on critically ill patients with ARDS, with a specific emphasis on the long-term complication metric of health-related quality of life (HRQL). Two researchers independently performed literature screening, data extraction, and methodological quality assessment.  **Results:** From 633 articles, one randomized controlled trial and five observational studies were included. The overall quality of the observational studies was moderate to high. In three studies (total sample size of 245), SF-36 scores indicated that ECMO survivors experienced a more pronounced decrease in quality of life compared to survivors of conventional mechanical ventilation (CMV). However, compared to CMV survivors, patients treated with ECMO had a significantly lower incidence of psychological disorders (two studies, total of 217 cases [ECMO vs. CMV groups]: weighted mean difference [WMD] for depressive symptoms was -1.31 [95% CI -1.98 to -0.64]; WMD for anxiety symptoms was -1.60 [95% CI -1.80 to -1.39]).  **Conclusion:** Further research is needed to validate these findings and to identify predictors associated with the prognosis of ECMO survivors.  **Additional Insights and Research Directions**  Many patients exhibit features of residual lung injury, such as chronic pulmonary fibrosis and decreased lung function, for up to five years after discharge.  Limited exercise function in survivors of severe ARDS is often unrelated to structural lung disease but is more likely associated with extrapulmonary muscle weakness.  Implementing early rehabilitation therapy for adult patients in the Intensive Care Unit (ICU) may improve outcomes. Consequently, current research priorities include how to safely implement rehabilitation strategies, optimize sedation practices, and gain a deeper understanding of the pharmacokinetics in high-risk, critically ill patients requiring ECMO therapy. | 1. Physiological Status - Motor Function and Musculoskeletal System - Muscle Function Status (Muscle Strength, Muscle Atrophy)  2. Physiological Status - Respiratory System - Chronic Cough, Sputum Production  3. Psychological Status - Existing Problems - Anxiety  4. Psychological Status - Existing Problems - Depression |
| Literature 35: Norkiene I et al. (2019)  Study Type: Prospective Follow-up Study  Quality Grade: Moderate | Quality of life (SF-36), PTSD, long-term survival | **Objective:** To investigate the long-term health-related quality of life (HRQoL) and the incidence of Post-Traumatic Stress Disorder (PTSD) in ECMO survivors.  **Methods:** All patients who started ECMO for refractory cardiogenic shock between 2009 and 2014 were included. The SF-36 was used to assess HRQoL, and the Impact of Event Scale-Revised (IES-R) questionnaire was used to assess the incidence of PTSD.  **Results:** Sixty-nine patients received V-A ECMO. Nineteen patients survived to discharge, and 15 patients were alive at the study cutoff point in June 2017; the mean follow-up time was 70.6 ± 10 months. The mean Physical Component Summary (PCS) and Mental Component Summary (MCS) scores for the long-term survivors were 46.1 ± 7 and 47.1 ± 8, respectively. Four of the 15 participants exhibited PTSD.  **Conclusion:** Despite a complex clinical course and a long recovery period, both the mental and physical recovery levels of ECMO survivors were satisfactory, comparable to population averages adjusted for age and pathology.  **Background and Study Rationale**  ① The findings from studies on long-term, self-reported health status after critical illness are contradictory.  On one hand, survivors of critical illness universally experience long-term deficits in physical function and cognitive abilities.  On the other hand, a few studies suggest that, compared to patients with chronic diseases, ECMO survivors exhibit good physical and social function.  ② There is still a lack of definitive evidence supporting a favorable long-term prognosis for ECMO survivors.  Study Aims: 1) To assess the long-term HRQoL and PTSD levels in patients who received ECMO after cardiac surgery. 2) To compare the HRQoL of ECMO survivors with a cohort of ICU survivors from the same institution.  **Follow-up Methodology**  **Follow-up Method:** Mail-out surveys or semi-structured telephone interviews.  **Follow-up Assessments:** SF-36, IES-R.  **Follow-up Recommendations and Insights**  ① Any medical intervention should be patient-centered; therefore, long-term health-related quality of life should be a crucial factor in decision-making. However, defining quality of life is challenging and subjective—any perception of well-being encompasses both considerations of basic human needs and broader life satisfaction.  ② Health-related quality of life assessment covers physical, mental, and social aspects of health (as described by the World Health Organization), but the perception of quality of life is personal. A level of quality of life that an observer might deem low may be acceptable to the individual.  ③ HRQoL Outcomes: The HRQoL of patients treated with ECMO was comparable to the levels seen in patients with coronary artery disease and after major cardiac surgery.  ④ PTSD: The duration of mechanical ventilation and disease severity may be important factors contributing to the high incidence of PTSD. | 1. Psychological Status - Existing Problems - Post-Traumatic Stress Disorder (PTSD)  2. Living Status - Activities of Daily Living - Self-Care Ability  3. Physiological Status - Circulatory System - Cardiac Insufficiency (Limb Edema, Fatigue, etc.) |
| Literature 36: Rossong H et al. (2023)  Study Type: Retrospective Cohort Study  Quality Grade: Moderate | Quality of life (EQ-5D-5L), anxiety, depression, PTSD, daily function (ADL/IADL) | **Follow-up Methodology**  **Follow-up Method:** Telephone interviews.  **Follow-up Assessments**:  ①Activities of Daily Living (ADLs)  ②Instrumental Activities of Daily Living (IADLs)  ③EQ-5D-5L  ④Hospital Anxiety and Depression Scale (HADS)  ⑤PTSD Checklist (PCL)  ⑥Paffenbarger Physical Activity Questionnaire  ⑦Modified Fried Frailty Criteria  ⑧Modified Decision Regret Scale (assesses psychological distress after a medical decision. Since patients receiving ECMO are often too critically ill to make autonomous decisions, most are made by a surrogate. Therefore, the scale was adapted to measure whether the survivor regrets the surrogate's decision and if they would make the same choice if given the opportunity again).  **Follow-up Results and Recommendations**  ① Functional limitations and mental health challenges can severely impact a patient's quality of life. Patients who receive ECMO often have underlying medical conditions and prolonged ICU and hospital stays, which can lead to critical illness polyneuromyopathy. It is therefore important to identify the specific barriers faced by ECMO survivors and to coordinate ongoing care in these areas.  ② Compared to V-A patients, V-V patients generally reported a lower quality of life, greater difficulties with activities of daily living/instrumental activities of daily living (ADLs/IADLs), and an increase in mental health problems.  ③ While V-A patients did experience more difficulties in some aspects of HRQoL compared to the population norm, the actual magnitude of the difference was smaller than that for the V-V group. The presence of some disability and psychological disorders among ECMO survivors underscores the necessity of providing long-term, interprofessional care for them, including physical therapy, occupational therapy, and mental health resources. Efforts should focus on ensuring accessible mental health services for ECMO survivors.  ④ Quality Improvement Project: The ICU Liberation Collaborative is a quality improvement initiative aimed at providing patient- and family-centered care. It employs the evidence-based, team-based ABCDEF bundle to address daily issues of pain, agitation, sedation, delirium, immobility, and sleep disruption.  * Interventions: Limiting sedative medications, providing guidance for early mobilization, standardizing circadian rhythms, equipping a dedicated palliative care team, and forming a rehabilitation team that includes physical therapy, occupational therapy, speech therapy, and nutritional services.  ⑤ Establish a standardized assessment process for effective communication with patients after their recovery. Implement a referral for psychiatric screening if Post-Traumatic Stress Disorder (PTSD) is identified during hospitalization, and ensure patients have access to continuous community care upon discharge.  ⑥ Decision Regret Scale: Both V-V and V-A patients had low scores, indicating minimal regret. Although the decision to initiate ECMO was not made autonomously by the patient, most survivors believed it was the correct choice and stated they would choose it again if necessary. | 1. Psychological Status - Existing Problems - Anxiety  2. Psychological Status - Existing Problems - Depression  3. Psychological Status - Existing Problems - Post-Traumatic Stress Disorder (PTSD)  4. Living Status - Activities of Daily Living - Self-Care Ability |
| Literature 37: Zeng X et al. (2024)  Study Type: Cross-sectional Study  Quality Grade: Moderate | Quality of life (SF-36), PTSD, self-care ability (Barthel Index), social function | **Follow-up Assessments**  ①SF-36 (Short Form-36 Health Survey)  ②Barthel Index (BI)  ③PTSD-SS (Post-Traumatic Stress Disorder Symptom Scale)  ④NHP-part II (Nottingham Health Profile - Part II)  **Conclusion**  Adult patients who survive ECMO experience poor long-term quality of life, with varying degrees of physical, psychological, and social dysfunction. Social function, in particular, is in urgent need of recovery. Long-term quality of life is influenced by multiple factors, including caregiver type, employment status, Barthel Index level, PTSD-SS level, NHP-part II level, and place of residence.  **Recommendations**  There is a need for professional medical teams to provide long-term management of chronic conditions through various methods, such as establishing dedicated ECMO outpatient clinics, online follow-up, and offline home visits. This approach can deliver more comprehensive and precise care, thereby improving overall long-term quality of life and extending the survival period of survivors.  **Detailed Findings and Analysis**  **① In terms of self-care ability:**  Self-care ability depends on the presence of complications after discharge that affect daily life. Complications such as lower limb ischemia, neurological damage, and chronic renal failure can persist long after discharge, may even require lifelong treatment, and directly or indirectly impact the patient's long-term quality of life. This is related to proactively establishing collateral circulation for lower limb reperfusion to reduce or avoid the occurrence of limb ischemia.  **② In terms of Post-Traumatic Stress Disorder (PTSD):**  The incidence rate was 17.0%. The followed-up patients had been discharged for more than three months, and their memory of the ECMO event had diminished or become blurred. With the companionship of family and gradual recovery from the illness, their psychological state tended to stabilize.  **③ In terms of social function:**  56.6% of patients had impaired social function, and only 37.7% of patients had returned to work.  **④** In the multivariate analysis of factors affecting the long-term quality of life of critically ill patients treated with ECMO, caregiver type was an independent factor influencing the Physical Component Summary (PCS) dimension.  When the caregiver was a spouse, the patient's quality of life score was lower. This may be related to the patient's physical function not yet having recovered, the patient needing additional assistance, and the spouse being the closest family member. | 1. Psychological Status - Existing Problems - Post-Traumatic Stress Disorder (PTSD)  2. Living Status - Activities of Daily Living - Self-Care Ability  3. Social and Family Support - Social Interaction and Network  4. Living Status - Medical Adherence - Lifestyle Changes |
| Literature 38: Hanuna M et al. (2024)  Study Type: Retrospective Study  Quality Grade: Moderate | Quality of life (SF-36), functional status (mRS), mid-term survival | **Objective**: To identify negative predictors of mid-term survival and to assess the health-related quality of life (HRQoL) and functional recovery of survivors.  **Methods:** Between 2017 and 2020, 142 patients received Extracorporeal Life Support (ECLS) after cardiac surgery. The median age was 66.0 [57.0-73.0] years, 67.6% were male, and the median EuroSCORE II was 10.5% [4.2-21.3]. At a median follow-up time of 2.2 [1.9-3.2] years, 48 patients were assessed for HRQoL using the SF-36 and the modified Rankin Scale (mRS).  **Results:** Survival rates at 3, 12, 24, and 36 months were 47%, 46%, 43%, and 43%, respectively (standard error: 4%). Multivariate Cox proportional hazards regression analysis identified preoperative EuroSCORE II (p=0.013), impaired renal function (p=0.010), cardiopulmonary bypass (CPB) duration (p=0.015), and lactate level before ECLS initiation (p=0.004) as independent predictors of mid-term mortality. At follow-up, 83.3% of survivors had no moderate-to-severe disability (mRS < 3). The SF-36 analysis showed a Physical Component Summary (PCS) score of 45.5 ± 10.2 and a Mental Component Summary (MCS) score of 50.6 ± 12.5.  **Conclusion:** ECLS for refractory post-cardiotomy shock (rPCS) is associated with acceptable mid-term survival, health-related quality of life, and functional status. Preoperative EuroSCORE II, impaired renal function, CPB duration, and pre-ECLS lactate levels were identified as negative predictors and should be incorporated into the clinical decision-making process.  **Follow-up Methodology**  **Follow-up Method:** Telephone follow-up.  **Follow-up Assessments:** SF-36, modified Rankin Scale (mRS).  **Follow-up Results and Recommendations**  ① ECLS treatment for rPCS is associated with high mortality within the first 3 months, but the survival rate stabilizes thereafter.  ② Patients with reduced preoperative ejection fraction, higher EuroSCORE II scores, higher preoperative lactate levels, and longer CPB duration had a significantly worse mid-term prognosis. Patients with a preoperative lactate level > 6.5 mmol/L and a EuroSCORE II score > 13% had a particularly poor prognosis.  ③ HRQoL at 2 years post-ECLS was favorable. In patients with decreased HRQoL, the impairment was primarily attributed to physical limitations (low PCS score). The overall recovery was positive, with only a small subset of patients (primarily those over 65 years of age at follow-up) experiencing moderate-to-severe physical disability.  Metrics such as preoperative EuroSCORE II, renal dysfunction, CPB time, and lactate levels may indicate a poorer prognosis and should be considered during decision-making. Furthermore, patients showing signs of low perfusion after cardiac surgery should undergo a rigorous evaluation for early ECLS support. | 1. Living Status - Activities of Daily Living - Self-Care Ability  2. Physiological Status - Circulatory System - Cardiac Insufficiency (Limb Edema, Fatigue, etc.)  3. Physiological Status - General Condition - Vital Signs |
| Literature 39: Provaznik Z et al. (2023)  Study Type: Retrospective Cohort Study  Quality Grade: Moderate | Short-term and long-term outcomes, neurological function status, impact of comorbidities | **Background:** The outcomes of elderly patients after V-V ECMO are unsatisfactory.  **Objective**: To evaluate the impact of advanced age on short- and long-term outcomes; a secondary objective was to analyze risk factors for impaired outcomes.  **Methods:** Between January 2006 and June 2020, 755 patients received V-V ECMO support in our department. Patients were stratified into groups based on age (18-49.9, 50-59.9, 60-69.9, and ≥70 years) and short- and long-term outcomes were retrospectively analyzed. Multivariate regression analysis was used to assess risk factors for in-hospital mortality and mortality during follow-up.  **Results:** The duration of V-V support was comparable across all median age groups (8-10 days, p = 0.256). Similarly, weaning rates were comparable across all age groups at 68.2%-76.5% (p = 0.354), but in-hospital mortality increased significantly with age (<50 years 30.1%/n=91 vs. 50-59.9 years 37.1%/n=73, vs. 60-69.9 years 45.6%/n=78 vs. ≥70 years 51.8%/n=44; p < 0.001). The older age groups also had significantly lower Cerebral Performance Category scores. Multivariate logistic analysis identified age, acute and chronic hemodialysis, bilirubin on day 1 of support, malignancy, and primary lung disease as relevant risk factors for in-hospital mortality. Age, coronary artery disease (CAD), the presence of another primary lung disease, malignancy, and immunosuppression were risk factors for mortality during follow-up.  **Conclusion:** In patients receiving V-V ECMO, advanced age is associated with more comorbidities, impaired short- and long-term outcomes, and worse neurological outcomes.  Risk Factors for Mortality  **Factors Influencing In-Hospital Mortality**  ① Age is a significant risk factor for in-hospital mortality. This negative impact may be due to the presence of comorbidities and a diminished biological capacity for recovery, making it difficult to cope with ECMO-related complications.  Advanced age is not only an independent risk factor for in-hospital mortality, but it is also associated with worse neurological function status and poorer quality of life during follow-up.  ② Other risk factors include: long-term hemodialysis, acute kidney injury (AKI) requiring dialysis, bilirubin level on the first day after ECMO implantation, malignancy, and another primary lung disease (e.g., pulmonary fibrosis, cystic fibrosis, vasculitis).  **Risk Factors for Mortality During Follow-up**  Age, associated comorbidities (coronary artery disease, another primary lung disease, and malignancy), and immunosuppressive therapy. | 1. Physiological Status - Nervous System - Consciousness and Arousal Level  2. Physiological Status - General Condition - Vital Signs  3. Physiological Status - Circulatory System - Use of Cardiovascular Drugs |
| Themes from Semi-structured Patient Interviews | Impact on sexual life and intimate relationships, social avoidance and stigma, demand for psychological crisis intervention |  | 1. Living Status - Occupation/Work Status, Impact on Sexual Life and Intimate Relationships - Impact on Sexual Life and Intimate Relationships (Optional)  2. Psychological Status - Existing Problems - Social Avoidance and Stigma (Optional)  3. Psychological Status - Coping Strategies/Interventions - Psychological Crisis Intervention Hotline 12356 (Optional) |

1、Cho SM, Hwang J, Chiarini G, Amer M, Antonini MV, Barrett N, Belohlavek J, Brodie D, Dalton HJ, Diaz R, Elhazmi A, Tahsili-Fahadan P, Fanning J, Fraser J, Hoskote A, Jung JS, Lotz C, MacLaren G, Peek G, Polito A, Pudil J, Raman L, Ramanathan K, Dos Reis Miranda D, Rob D, Salazar Rojas L, Taccone FS, Whitman G, Zaaqoq AM, Lorusso R. Neurological monitoring and management for adult extracorporeal membrane oxygenation patients: Extracorporeal Life Support Organization consensus guidelines. Crit Care. 2024 Sep 6;28(1):296. doi: 10.1186/s13054-024-05082-z. Erratum in: Crit Care. 2024 Oct 7;28(1):327. doi: 10.1186/s13054-024-05107-7.

2、Chen Jinmeng, Lu Kun. Analysis of Factors Affecting the Quality of Life and Complications of Critically Ill Survivors after ECMO Treatment. Heilongjiang Journal of Traditional Chinese Medicine, 2024, 53(01): 123-125.

3、Wang Fengzhen, Zhang Yuhao, Wu Shujing, et al. A qualitative study on the post-discharge life experiences of patients treated with extracorporeal membrane oxygenation. Evidence-Based Nursing, 2024, 10(05): 942-946.

4、Chen Yuanyuan, Yao Ruishan, Wan Jia, et al. Meta-integration of qualitative research on the life experiences and needs of patients undergoing extracorporeal membrane oxygenation treatment. Journal of Nursing, 2024, 39(22): 43-47 + 51.

5、Thalanany MM, Mugford M, Hibbert C, Cooper NJ, Truesdale A, Robinson S, Tiruvoipati R, Elbourne DR, Peek GJ, Clemens F, Hardy P, Wilson A; CESAR Trial Group. Methods of data collection and analysis for the economic evaluation alongside a national, multi-centre trial in the UK: conventional ventilation or ECMO for Severe Adult Respiratory Failure (CESAR). BMC Health Serv Res. 2008 Apr 30;8:94. doi: 10.1186/1472-6963-8-94. PMID: 18447931; PMCID: PMC2387150.

6、Serpa Neto A, Higgins AM, Bailey MJ, Anderson S, Bernard S, Fulcher BJ, Jones A, Linke NJ, Board JV, Brodie D, Buhr H, Burrell AJC, Cooper DJ, Fan E, Fraser JF, Gattas DJ, Hopper IK, Huckson S, Litton E, McGuinness SP, Nair P, Orford N, Parke RL, Pellegrino VA, Pilcher DV, Dicker C, Reddi BAJ, Stub D, Trapani TV, Udy AA, Hodgson CL; EXCEL Study Investigators on behalf of the International ECMO Network (ECMONet). Long-Term Functional Outcomes in the First 12 Months After VA-ECMO in Adult Patients: A Prospective, Multicenter Study. Circ Heart Fail. 2025 Jun;18(6):e012476. doi: 10.1161/CIRCHEARTFAILURE.124.012476. Epub 2025 Apr 29. PMID: 40298907.

7、Matthieu S ,Elie Z ,Hadrien R , et al.The PRESERVE mortality risk score and analysis of long-term outcomes after extracorporeal membrane oxygenation for severe acute respiratory distress syndrome.[J].Intensive care medicine,2013,39(10):1704-13.

8、Fernando SM, Scott M, Talarico R, et al. Association of Extracorporeal Membrane Oxygenation With New Mental Health Diagnoses in Adult Survivors of Critical Illness. JAMA. 2022;328(18):1827–1836. doi:10.1001/jama.2022.17714

9、Risnes I, Wagner K, Nome T, Sundet K, Jensen J, Hynås IA, Ueland T, Pedersen T, Svennevig JL. Cerebral outcome in adult patients treated with extracorporeal membrane oxygenation. Ann Thorac Surg. 2006 Apr;81(4):1401-6. doi: 10.1016/j.athoracsur.2005.10.008. PMID: 16564280.

10、Oh TK, Cho HW, Lee HT, Song IA. Chronic respiratory disease and survival outcomes after extracorporeal membrane oxygenation. Respir Res. 2021 Jul 5;22(1):195. doi: 10.1186/s12931-021-01796-8. PMID: 34225713; PMCID: PMC8256197.

11、Tramm R, Ilic D, Sheldrake J, Pellegrino V, Hodgson C. Recovery, Risks, and Adverse Health Outcomes in Year 1 After Extracorporeal Membrane Oxygenation. Am J Crit Care. 2017 Jul;26(4):311-319. doi: 10.4037/ajcc2017707. PMID: 28668917.

12、Shao C, Wang L, Yang F, Wang J, Wang H, Hou X. Quality of Life and Mid-Term Survival in Patients Receiving Extracorporeal Membrane Oxygenation After Cardiac Surgery. ASAIO J. 2022 Mar 1;68(3):349-355. doi: 10.1097/MAT.0000000000001473. PMID: 35213884.

13、Tiedebohl JM, DeFabio ME, Bell T, Buchko BL, Woods AB. ECMO survivors' quality of life and needs after discharge: A descriptive, comparative cross-sectional pilot study. Intensive Crit Care Nurs. 2020 Aug;59:102829. doi: 10.1016/j.iccn.2020.102829. Epub 2020 Mar 12. PMID: 32173238.

14、Hodgson CL, Higgins AM, Bailey MJ, Anderson S, Bernard S, Fulcher BJ, Koe D, Linke NJ, Board JV, Brodie D, Buhr H, Burrell AJC, Cooper DJ, Fan E, Fraser JF, Gattas DJ, Hopper IK, Huckson S, Litton E, McGuinness SP, Nair P, Orford N, Parke RL, Pellegrino VA, Pilcher DV, Sheldrake J, Reddi BAJ, Stub D, Trapani TV, Udy AA, Serpa Neto A; EXCEL Study Investigators on behalf of the International ECMO Network and the Australian and New Zealand Intensive Care Society Clinical Trials Group. Incidence of death or disability at 6 months after extracorporeal membrane oxygenation in Australia: a prospective, multicentre, registry-embedded cohort study. Lancet Respir Med. 2022 Nov;10(11):1038-1048. doi: 10.1016/S2213-2600(22)00248-X. Epub 2022 Sep 26. PMID: 36174613.

15、Kurniawati ER, Rutjens VGH, Vranken NPA, Delnoij TSR, Lorusso R, van der Horst ICC, Maessen JG, Weerwind PW. Quality of life following adult veno-venous extracorporeal membrane oxygenation for acute respiratory distress syndrome: a systematic review. Qual Life Res. 2021 Aug;30(8):2123-2135. doi: 10.1007/s11136-021-02834-0. Epub 2021 Apr 7. PMID: 33826058; PMCID: PMC8024673.

16、Grasselli G, Scaravilli V, Tubiolo D, Russo R, Crimella F, Bichi F, Morlacchi LC, Scotti E, Patrini L, Gattinoni L, Pesenti A, Chiumello D. Quality of Life and Lung Function in Survivors of Extracorporeal Membrane Oxygenation for Acute Respiratory Distress Syndrome. Anesthesiology. 2019 Apr;130(4):572-580. doi: 10.1097/ALN.0000000000002624. PMID: 30875355.

17、Kanji HD, Chouldechova A, Harris-Fox S, Ronco JJ, O'dea E, Harvey C, Shuster C, Thiara S, Peek GJ. Quality of life and functional status of patients treated with venovenous extracorporeal membrane oxygenation at 6 months. J Crit Care. 2021 Dec;66:26-30. doi: 10.1016/j.jcrc.2021.07.010. Epub 2021 Aug 17. PMID: 34416505.

18、Ozgur MM, Altinay E, Ogus H, Acar RD, Atagun Guney P, Kirali K. Functional and Social Recovery and Outcomes After Extracorporeal Membrane Oxygenation Support in COVID-19 Patients. ASAIO J. 2025 May 1;71(5):396-402. doi: 10.1097/MAT.0000000000002337. Epub 2024 Oct 15. PMID: 39405379.

19、Guenther SPW, Cheaban R, Hoepner L, Weinrautner N, Kirschning T, Al-Khalil R, Bruenger F, Serrano MR, Barndt I, Wiemer M, Niedermeyer J, Rudloff M, Helms S, Schramm R, Gummert JF. Functional Status and Quality of Life 6 Months After Extracorporeal Membrane Oxygenation Therapy for COVID-19-Related Pulmonary Failure. ASAIO J. 2023 Oct 1;69(10):942-949. doi: 10.1097/MAT.0000000000001993. Epub 2023 May 31. PMID: 37256794.

20、Spangenberg T, Schewel J, Dreher A, Meincke F, Bahlmann E, van der Schalk H, Kreidel F, Frerker C, Stoeck M, Bein B, Kuck KH, Ghanem A. Health related quality of life after extracorporeal cardiopulmonary resuscitation in refractory cardiac arrest. Resuscitation. 2018 Jun;127:73-78. doi: 10.1016/j.resuscitation.2018.03.036. Epub 2018 Apr 4. PMID: 29626610.

21、Chen KH, Lee PS, Tsai FC, Weng LC, Yeh SL, Huang HC, Lin SS. Health-related outcomes of extracorporeal membrane oxygenation in adults: A cross-sectional study. Heart Lung. 2022 Mar-Apr;52:76-85. doi: 10.1016/j.hrtlng.2021.11.008. Epub 2021 Dec 12. PMID: 34911020.

22、Oude Lansink-Hartgring A, Miranda DDR, Mandigers L, Delnoij T, Lorusso R, Maas JJ, Elzo Kraemer CV, Vlaar APJ, Raasveld SJ, Donker DW, Scholten E, Balzereit A, van den Brule J, Kuijpers M, Vermeulen KM, van den Bergh WM; Dutch ECLS Study group. Health-related quality of life, one-year costs and economic evaluation in extracorporeal membrane oxygenation in critically ill adults. J Crit Care. 2023 Feb;73:154215. doi: 10.1016/j.jcrc.2022.154215. Epub 2022 Nov 17. PMID: 36402123.

23、Chen KH, Tsai FC, Tsai CS, Yeh SL, Weng LC, Yeh LC. Problems and health needs of adult extracorporeal membrane oxygenation patients following hospital discharge: A qualitative study. Heart Lung. 2016 Mar-Apr;45(2):147-53. doi: 10.1016/j.hrtlng.2015.12.005. Epub 2016 Feb 4. PMID: 26853922.

24、Wang F, Zhang Y, Wu S, Xie H, Lin D, Wen X, Duan Z, Lu Y, Liu Z, Hu S, Liu J. Post-discharge experiences of patients with extracorporeal membrane oxygenation support: A qualitative study. Perfusion. 2024 Jan;39(1):189-200. doi: 10.1177/02676591221135165. Epub 2022 Oct 25. PMID: 36282873.

25、Hsieh FT, Huang GS, Ko WJ, Lou MF. Health status and quality of life of survivors of extra corporeal membrane oxygenation: a cross-sectional study. J Adv Nurs. 2016 Jul;72(7):1626-37. doi: 10.1111/jan.12943. Epub 2016 Feb 23. PMID: 26909658.

26、Ayers B, Bjelic M, Kumar N, Wood K, Barrus B, Prasad S, Gosev I. Long-term renal function after venoarterial extracorporeal membrane oxygenation. J Card Surg. 2021 Mar;36(3):815-820. doi: 10.1111/jocs.15277. Epub 2021 Jan 8. PMID: 33416196.

27、Lansink-Hartgring AO, van der Bij W, Verschuuren EA, Erasmus ME, de Vries AJ, Vermeulen KM, van den Bergh WM. Extracorporeal Life Support as a Bridge to Lung Transplantation: A Single-Center Experience With an Emphasis on Health-Related Quality of Life. Respir Care. 2017 May;62(5):588-594. doi: 10.4187/respcare.05300. Epub 2017 Mar 21. PMID: 28325778.

28、O'Brien SG, Carton EG, Fealy GM. Long-Term Health-Related Quality of Life After Venovenous Extracorporeal Membrane Oxygenation. ASAIO J. 2020 May;66(5):580-585. doi: 10.1097/MAT.0000000000001042. PMID: 31425257.

29、Harley O, Reynolds C, Nair P, Buscher H. Long-Term Survival, Posttraumatic Stress, and Quality of Life post Extracorporeal Membrane Oxygenation. ASAIO J. 2020 Aug;66(8):909-914. doi: 10.1097/MAT.0000000000001095. PMID: 32740351.

30、Knudson KA, Gustafson CM, Sadler LS, Whittemore R, Redeker NS, Andrews LK, Mangi A, Funk M. Long-term health-related quality of life of adult patients treated with extracorporeal membrane oxygenation (ECMO): An integrative review. Heart Lung. 2019 Nov-Dec;48(6):538-552. doi: 10.1016/j.hrtlng.2019.08.016. PMID: 31711573.

31、Dardik G, Ning Y, Kurlansky P, Almodovar Cruz G, Vinogradsky A, Fried J, Topkara VK, Takeda K. Long-term outcomes of patients bridged to recovery with venoarterial extracorporeal life support. Perfusion. 2024 Nov;39(8):1629-1635. doi: 10.1177/02676591231206524. Epub 2023 Oct 20. PMID: 37861303.

32、Turgeon J, Venkatamaran V, Englesakis M, Fan E. Long-term outcomes of patients supported with extracorporeal membrane oxygenation for acute respiratory distress syndrome: a systematic review and meta-analysis. Intensive Care Med. 2024 Mar;50(3):350-370. doi: 10.1007/s00134-023-07301-7. Epub 2024 Jan 10. PMID: 38197932.

33、von Bahr V, Kalzén H, Frenckner B, Hultman J, Frisén KG, Lidegran MK, Diaz S, Malfertheiner MV, Millar JE, Dobrosavljevic T, Eksborg S, Holzgraefe B. Long-term pulmonary function and quality of life in adults after extracorporeal membrane oxygenation for respiratory failure. Perfusion. 2019 Apr;34(1_suppl):49-57. doi: 10.1177/0267659119830244. PMID: 30966900.

34、Wilcox ME, Jaramillo-Rocha V, Hodgson C, Taglione MS, Ferguson ND, Fan E. Long-Term Quality of Life After Extracorporeal Membrane Oxygenation in ARDS Survivors: Systematic Review and Meta-Analysis. J Intensive Care Med. 2020 Mar;35(3):233-243. doi: 10.1177/0885066617737035. Epub 2017 Oct 19. PMID: 29050526.

35、Norkiene I, Jovaisa T, Scupakova N, Janusauskas V, Rucinskas K, Serpytis P, Laurusonis K, Samalavicius R. Long-term quality of life in patients treated with extracorporeal membrane oxygenation for postcardiotomy cardiogenic shock. Perfusion. 2019 May;34(4):285-289. doi: 10.1177/0267659118815291. Epub 2018 Dec 19. PMID: 30565505.

36、Rossong H, Debreuil S, Yan W, Hiebert BM, Singal RK, Arora RC, Yamashita MH. Long-term survival and quality of life after extracorporeal membrane oxygenation. J Thorac Cardiovasc Surg. 2023 Aug;166(2):555-566.e2. doi: 10.1016/j.jtcvs.2021.10.077. Epub 2022 Feb 18. PMID: 35346489.

37、Xiaoting Zeng, Fuxun Yang, Xiaoxiu Luo et al. Long-term quality of life in adult extracorporeal membrane oxygenation survivors: A single-center, cross-sectional study, 12 March 2024, PREPRINT (Version 1) available at Research Square [https://doi.org/10.21203/rs.3.rs-3990827/v1]

38、Hanuna M, Herz G, Stanzl AL, Li Y, Mueller CS, Kamla CE, Scherer C, Wassilowsky D, Juchem G, Orban M, Peterss S, Hagl C, Joskowiak D. Mid-Term Outcome after Extracorporeal Life Support in Postcardiotomy Cardiogenic Shock: Recovery and Quality of Life. J Clin Med. 2024 Apr 12;13(8):2254. doi: 10.3390/jcm13082254. PMID: 38673527; PMCID: PMC11050874.

39、Provaznik Z, Philipp A, Müller T, Kostiantyn K, Lunz D, Schmid C, Floerchinger B. Outcome after veno-venous extracorporeal membrane oxygenation in elderly compared to younger patients: A 14-year retrospective observational study. Artif Organs. 2023 May;47(5):882-890. doi: 10.1111/aor.14454. Epub 2022 Nov 14. PMID: 36325937.
